# Human bone marrow harbors cells with neural crest-associated characteristics like human adipose and dermis tissues

**DOI:** 10.1371/journal.pone.0177962

**Published:** 2017-07-06

**Authors:** Cécile Coste, Virginie Neirinckx, Anil Sharma, Gulistan Agirman, Bernard Rogister, Jacques Foguenne, François Lallemend, André Gothot, Sabine Wislet

**Affiliations:** 1 GIGA Neurosciences, University of Liège, Liège, Belgium; 2 Department of Neuroscience, Karolinska Institute, Stockholm, Sweden; 3 GIGA Development, Stem Cells and Regenerative Medicine, University of Liège, Liège, Belgium; 4 Department of Neurology, University of Liège, Liège, Belgium; 5 Department of Laboratory Hematology and Immunology, CHU Liège, Liège, Belgium; 6 Hematology Research Unit, GIGA-I3, University of Liège, Liège, Belgium; Instituto Butantan, BRAZIL

## Abstract

Adult neural crest stem-derived cells (NCSC) are of extraordinary high plasticity and promising candidates for use in regenerative medicine. Several locations such as skin, adipose tissue, dental pulp or bone marrow have been described in rodent, as sources of NCSC. However, very little information is available concerning their correspondence in human tissues, and more precisely for human bone marrow. The main objective of this study was therefore to characterize NCSC from adult human bone marrow. In this purpose, we compared human bone marrow stromal cells to human adipose tissue and dermis, already described for containing NCSC. We performed comparative analyses in terms of gene and protein expression as well as functional characterizations. It appeared that human bone marrow, similarly to adipose tissue and dermis, contains *NESTIN*^+^ / *SOX9*^+^ / *TWIST*^+^ / *SLUG*^+^ / *P75*^*NTR*+^ */ BRN3A*^+^/ *MSI1*^+^/ *SNAIL1*^+^ cells and were able to differentiate into melanocytes, Schwann cells and neurons. Moreover, when injected into chicken embryos, all those cells were able to migrate and follow endogenous neural crest migration pathways. Altogether, the phenotypic characterization and migration abilities strongly suggest the presence of neural crest-derived cells in human adult bone marrow.

## Introduction

The neural crest (NC) is an embryonic ephemeral structure present in vertebrates and located at the margin of the neural tube [1,2]. Those neural crest-derived stem cells (NCSC) have the unique property to migrate from the dorsal neuroepithelium and invade multiple tissues. The initiation of cell migration involved an epithelial-to-mesenchymal transition (EMT) which triggers multiple molecular and cellular machineries [3]. After completing EMT, NCSC delaminate and start migrating to their final destination [4]. A multitude of neural and non-neural mature cell types originate from this NC structure [5,6]. Indeed, it gives rise to the peripheral nervous system (PNS) neurons and glia (in sympathetic, parasympathetic, enteric, and partly sensory ganglia) [7], the pigmented cells of the skin (melanocytes) [8] and some neuro-endocrine cells (in the carotid body, adrenal medulla, and thyroid gland) [5,6], to diverse mesenchymal cell types like craniofacial bones, cartilages, tendons, connective cells, and adipose tissue [5,6].

In 2000, Jiang and collaborators [9] developed a two-component genetic system based on Cre/Lox recombination to indelibly label the entire mouse NC population at the time of its formation, and to detect its progeny at any time thereafter. In such a model, the fate of NCSC was mapped *in vivo* by mating ROSA26 Cre reporter (R26R) mice, expressing beta-galactosidase upon Cre-mediated recombination, with mice expressing Cre recombinase under the control of the *Wnt1* promoter. Using this transgenic model (or a similar one), the presence of multipotent NC-derived cells was confirmed in adult hair follicles [10], adult skin [11–16], adult bone marrow [17, 18 retracted in 68], the dental pulp [19–21], the cornea [22], the olfactory epithelium [23] and more recently in the adipose tissue [24–26].

The presence of multipotent post-migratory NCSC, in various adult mammal tissues, opens exciting new avenues for research in translational medicine. Indeed, adult NCSC are of extraordinary high plasticity and promising candidates for a use in cell therapy protocols. Unfortunately, very little information is available concerning the presence of NCSC in human adult bone marrow. We therefore decided to analyze human bone marrow, classically used as a source of mesenchymal stem cells (MSC), to determine whether a subset of those cells could be derived from the NC. In 2006, Pierret and collaborators hypothesized that all adult stem cells (including bone marrow-derived stem cells) originate from neural crest stem cells [27]. So far, stem cells exhibiting NC characteristics were identified in human dermis [28–32], hair follicle [30,31], dental pulp [33–36], periodontal ligament [37,38], cornea [39], inferior turbinate [40], olfactory or oral mucosa [41,42] and adipose tissue [43]. We therefore decided to compare and characterize human bone marrow cells to human adipose tissue and dermis. We compared gene and protein expression among those three cell types, and conducted functional characterizations. Finally, we injected those cells into chicken embryos and observed their migration profiles. Altogether, our results strongly suggest the presence of NCSC in human bone marrow as already observed for dermis and adipose tissue.

## Materials and methods

### Ethical statement

Rodents were bred at the University of Liège Central Animal facility. Experiments on animals were performed following rules set by the University of Liege ethics committee (Commission d’éthique animal ULg, Belgium, ethical permit 1038). Human cells obtained after iliac crest puncture were collected in accordance with ethical committee rules (ethical permit B707201214335, written consent). Human stromal stem cells from adipose tissue and dermis were isolated from residual tissue obtained from surgery. The use of residual material do not require ethical permit, however, patients were advised (written information) that all residual material from surgery could be used for research purpose. If they don’t agree with such a procedure, they have to fill out a form, otherwise, it is consider as a Yes tacit. Local Ethical committee approved both consent procedures (Comité d'Ethique Hospitalo-Facultaire Universitaire de Liège).

### Animal care

*Wnt1-Cre/R26R*-LacZ double transgenic mice were used to isolate NCSC and MSC clones from adult bone marrow stroma [17, 18 retracted in 68]. Transgenic C57BL/6J mice came from Jackson laboratory (The Jackson Laboratory, Bar Harbor, Maine, USA, http://www.jax.org).

### Human bone marrow stromal cell isolation

Human bone marrow stromal cells were obtained using iliac crest puncture from 23 to 29 year-old healthy women. Cells were initially isolated and cultivated according to our previously described protocol [59,60]. Human bone marrow samples were obtained from healthy volunteer adults. Mononuclear cells were isolated by centrifugation over Ficoll Paque^™^ Plus (GE Healthcare, Diegem, Belgium) and washed in PBS (Lonza, Verviers, Belgium). Cells were seeded at a density of 5 x 10^5^ cells/cm^2^ in MSC growth medium (Lonza) and incubated into humidified incubator at 37°C and 5% CO_2_.

### Human adipose tissue and dermis-derived stromal stem cell isolation

Human stromal stem cells from adipose tissue and dermis were isolated from residual tissue obtained after abdominoplasties or breast reductions on 17, 21, 23, 27 and 34 year-old healthy women. Cells were isolated and cultivated as previously described [61]. Adipose tissue stem cell preparation: immediately after surgery, adipose tissue was washed 3 times in PBS-glucose, then minced and incubated with 1% collagenase type I (C0130, Sigma-Aldrich, St-Louis, MO, USA) during 1 hour at 37°C and 5% CO_2_; 5 mg/ml of DNase (2 000 Kunit/ml) (AMPD1 Sigma-Aldrich) was added in order to decrease viscosity. Mature adipocytes and undigested fragments were separated from the pellet containing stromal vascular fraction (SVF) by centrifugation at 500 g, during 15 minutes. The SVF pellet was re-suspended in MesenCult culture medium (MesenCult, Stem Cells Technologies, Grenoble, France) supplemented with penicillin-streptomycin in 25 cm^2^ T-flask (Corning, New York, NY, USA) into a humidified incubator at 37°C and 5% CO_2_. Skin-derived stem cell preparation: after removal of epidermis and hypodermis, dermis was washed 3 times in PBS-glucose. 3 mm^2^-pieces were cut from the entire dermis explant and placed into 24-well plates. Dermis pieces were covered by culture medium (MesenCult culture medium with penicillin-streptomycin) and incubated into humidified incubator at 37°C and 5% CO_2_. Explants were cultivated for one week, allowing NCSC migration from explants, then removed.

### Stromal stem cell culture

Bone marrow, adipose tissue and skin-derived stem cells were all cultivated after isolation in MesenCult culture medium. After reaching confluence, cells were resuspended using 0.05% Trypsin-EDTA (Life Technologies, Carlsbald, CA, USA), and then sub-cultured (100 000 cells/25 cm^2^ (Corning) at 37°C, in a humid atmosphere containing 5% CO_2_. All experiments were performed on cells at passage 2 to 8.

### IPS-derived neural stem cells

Those cells were used as negative control for chicken injection and migration experiments. Those cells were a gift from Anna Falks laboratory at the Karolinska institutet. Those NSC from human iPSC cells were cultured in DMEM/F12 medium supplied with EGF and FGF2.

### Sphere-forming protocol

For promoting of sphere formation, 15 000 cells/ml were cultured in 25 cm^2^ T-flasks coated with polyHEMA (P3932, Sigma-Aldrich) during 12 days, in Dulbecco’s modified eagle medium/F12 (DMEM/F12) (Gibco, Paisley, United Kingdom) supplemented with B27 without vitamin A (1:50; Gibco), 20 ng/ml epidermal growth factor (EGF) (Peprotech, Princeton, NJ, USA) and 10 ng/ml basic fibroblast growth factor bFGF (Peprotech). EGF and bFGF were added every 48 hours.

### CFU assay

Colony forming unit (CFU) potential was assessed for bone marrow, adipose tissue and skin-derived stem cells. 50 cells/cm^2^ were cultivated in 9 cm^2^ dishes (Thermo Scientific, Waltham, MA, USA) during 14 days at 37°C, in a humid atmosphere containing 5% CO_2_; Mesencult culture medium was changed twice a week. Number of colonies is counted after 14 days.

### Flow cytometry phenotyping

Characterization of cell surface markers was performed on each of the three cell types by flow cytometry. Antibodies used for stromal cell characterization at 20 μl antibody for 1 x 10^6^ cells in 100 μl sample are listed as follow: anti-CD80 (clone L307) and anti-CD31 (clone WM59) conjugated to fluorescein isothiocyanate (FITC), anti-CD73 (clone AD2), anti-CD90 (clone 5E10) and anti-CD34 (clone 581) conjugated to phycoerythrin (PE) and anti-CD14 (clone M5E2) conjugated to allophycocyanin (APC) and anti-CD19 (clone SJ25C1) conjugated to phycoerythrin-cyanine 7 (PC7), anti-CD45 (clone HI30) conjugated to V500 (all from BD-Biosciences, Erembodegem, Belgium), FITC-anti-HLA-DR (clone B8.12.2) and PE-anti-CD105 (clone TEA3/17.11) (Beckman Coulter, Pasadena, CA, USA). Labeling and analysis protocol was previously described by Briquet, [59]. Briefly, cells were trypsinised and 500 000 cells were re-suspended into 100 μl of phosphate-buffered saline (PBS) (Lonza, Basel, Switzerland). After a 20-minutes incubation with the antibody cocktail at 4°C, cells were then washed 3 times using Hank’s balanced salt solution (HBSS) (Gibco) and centrifuged at 20°C at 600 g, for 5 minutes. Supernatant was removed and cell pellet was fixed in 300 μl of PBS supplemented with 1% paraformaldehyde (PFA) (158127, Sigma-Aldrich). Cells were then filtrated through a 70 μm-filter and sorted using a NAVIOS flow cytometer (Beckman Coulter).

### In-vitro multipotency assessment

The three cell types were differentiated into adipocytes, osteocytes, chondrocytes, Schwann cells, melanocytes and neurons. Adipogenic, osteogenic and chondrogenic differentiation assays were performed as described by Pittenger *et al*., [62]. Adipocyte differentiation was induced by culturing 20 000 cells per well in 24-well plate (Corning) for 3 weeks, in DMEM (Lonza) supplemented with 5 μg/ml insulin (I3536, Sigma-Aldrich), 60 μM indomethacin (I7378, Sigma-Aldrich), 1 μM dexamethasone (D4902, Sigma-Aldrich), 10% fetal bovine serum (FBS) (Lonza) and penicillin-streptomycin (Gibco). Lipid vacuoles were detected using Oil-Red-O staining. Briefly, cells were incubated for 10 minutes into 1.5 mg/ml of Oil-Red-O (O0625, Sigma-Aldrich) dissolved in isopropanol. After several washes with milliQ water, nuclei were colored using hematoxylin staining (H9627, Fluka analytical: Sigma-Aldrich) for 5 minutes. Cells were washed several times with milliQ water and then mounted using Aquamount Mountant (BDH, Poole, England). Osteoblasts differentiation was induced by culturing 3 000 cells per well in 24-well plate (Corning) for three weeks in DMEM with 10 mM β-glycero phosphate (G9422, Sigma-Aldrich), 0.1 μM dexamethasone, 60 μM ascorbate-2-phosphate (A8960, Sigma-Aldrich), 10% FBS and penicillin-streptomycin. Calcium deposits were detected using alizarin red (A5533, Sigma-Aldrich; 20 mg/ml, pH 4.2). Cells were colored during 8 minutes, washed with milliQ water and mounted using Q Path Safemount (Labonord, Templemars, France). Chondrogenic differentiation was induced by culturing 500 000 cells in micropellets in polypropylene tubes (Corning) for three weeks in DMEM supplemented with 10 ng/ml TGF-β3 (Peprotech), 50 μM ascorbate 2-phosphate, 0.5 μg/ml insulin, 10% FBS, and penicillin-streptomycin. Pellets were paraffin-embedded and sliced at 4.5 μm using a microtome. Slices were then dewaxed and hydrated prior to alcian blue staining: 5 minutes in alcian blue solution then rinse during 3 minutes in water from the tap and 1 minute in milliQ water. After the first step, 10 minutes in periodic acid then rinse during 3 minutes in water from the tap and 1 minute in milliQ water. Afterward, 15 minutes in periodic acid Schiff’s solution then rinse during 3 minutes in water from the tap and 1 minute in milliQ water. Finally, 20 seconds in hematoxylin solution modified according to Gill III and again 3 minutes in water from the tap followed by dehydration process (Ethanol 70%, 96%, 100%, xylene). Slides were then mounted in Aquamount Mountant. Schwann cell differentiation was induced by culturing 3 000 cells per well in 24-well plates for two weeks in DMEM/F12 supplemented with N2 supplement diluted 100x (Gibco), 10 ng/ml ciliary neurotrophic factor (CNTF) (R&D system, Minneapolis, MS, USA), 10 ng/ml bFGF (Peprotech), 1 mM dbcAMP (D0260, Sigma-Aldrich), 20 ng/ml neuregulin-1β (R&D system) and penicillin-streptomycin [63]. Schwann cells were stained using anti-S100 (1:200; Dako, Glostrup, Denmark), anti-MBP (1:250 Millipore) and anti-P0 antibodies (1:100 Millipore) (see below). Melanocyte differentiation was induced by culturing 3 000 cells per well in 24-well plates coated with 10 ng/ml fibronectin (F2006, Sigma-Aldrich) for 3 to 5 weeks, in DMEM low-glucose supplemented with 20% MCDB 201 (M6770, Sigma-Aldrich), 50 ng/ml Wnt3A (Peprotech), 50 ng/ml stem cell factor (SCF) (Peprotech), 100 nM endothelin-3 (Peprotech), 20 pM cholera toxin (C8052, Sigma-Aldrich), 4 ng/ml bFGF (Peprotech), 100 μM L-ascorbic acid (A4403, Sigma-Aldrich), 0.05 μM dexamethasone, 1x ITS (Gibco), 1 mg/ml linoleic acid-albumin from bovine serum (L9530, Sigma-Aldrich) [64,65]. Melanocytes were stained using anti-TYRP1 (tyrosinase-related protein 1) antibody (1:100; Abcam, Cambridge, United Kingdom) (see below). Neuronal differentiation was induced by culturing during two weeks 3 000 cells per well in 24-well plates previously coated with poly-D-lysine (Sigma-Aldrich). Cells were cultivated in DMEM/F12 supplemented with 1.5 mM adenosine 3’,5’-cyclic monophosphate sodium (AMPc) (A6885, Sigma-Aldrich), 10 ng/ml nerve growth factor (NGF) (Peprotech), 10 ng/ml glial cell line-derived neurotrophic factor (GDNF) (Peprotech), 10 ng/ml brain-derived neurotrophic factor (BDNF) (Peprotech), and B27 with vitamin A (1:50) (Gibco) [66]. Neurons were stained using rabbit anti-NeuN antibody (1:500; Millipore) and rat anti-Neurofilament antibody (1:500; Millipore). All cultures were maintained a 100% humidified atmosphere at 37°C, 5% CO_2_ and medium was changed twice a week. Before staining, cells were fixed with 4% PFA in PBS during 10 minutes at room temperature (4°C overnight for chondrocytes).

### Cell injections into chick embryos

Experiments performed on chicken embryos were carried out in accordance with Karolinska Institutet ethical committee rules. Chick embryos were obtained from *Gallus gallus domesticus* fertilized eggs (E S F—Produkter i Estuna AB) and stored at room temperature. Before injection, eggs were incubated during 72 hours at 38.5°C in a humidified incubator (60% humidity), they were selected at Hamburger-Hamilton stage 18 (HHSt18), based on their morphology. We injected 0.1% (w/v in PBS) Fast Green (Amresco, VWR, Sweden) under the chorioallantoic membrane to aid visualization of the underlying embryo. Using an electrochemically sharpened tungsten wire we opened the vitelline membrane and introduced a small lesion in the thoracolumbar region directly dorsolateral to the NT, in-between two somites and thus directly in the neural crest migration stream [12,67]. About 300 adherent cells in suspension or one sphere (corresponding approximately 300 cells) are placed directly in the lesion. The egg was sealed and incubated 38.5°C in 60% humidity. Three days later (HHSt30), embryos were removed from the shell, decapitated and fixed in PBS containing 2% PFA and 15% (w/v) sucrose in PBS for 2 hours at 4°C with gentle agitation, before incubation overnight at 4°C in 30% (w/v) sucrose in PBS. Embryos were frozen in Tissue O.C.T. Q-Path (Labonord) and 14 μm embryo slices were cut using cryostat microm HM 560 (LCAM—van Leeuwenhoek Centre for Advanced Microscopy, Amsterdam, Netherland).

### Immunostainings

#### Immunocytochemistry

Cell cultures were fixed with 4% PFA for 10 minutes at room temperature, and then incubated for 1 hour with 10% normal donkey serum in PBS-T (PBS supplemented with 0.3% Triton X-100 for intracellular antigens). Antibodies used were: mouse anti-NESTIN (1:100; Novus Biologicals, Littleton, CO, USA), rabbit anti-P75^NTR^ (1:200; Millipore), mouse anti-SOX10 (1:200; R&D system), rabbit anti-S100β (1:200; Dako), rat anti-MBP (1:250 Millipore), chicken anti-P0 (1:100 Millipore), rabbit anti-TYRP1 (1:100; Abcam), rabbit anti-NeuN (1:500; Millipore), rat anti-Neurofilament (1:500; Millipore), rabbit anti-SOX9 (1:500; Millipore) and rabbit anti-TWIST (1:500; Millipore). Cells were incubated with primary antibodies diluted in PBS or PBS-T for 2 hours at room temperature or overnight at 4°C. After three PBS washes, cells were incubated with Rhodamine Red X-conjugated secondary antibody (1:500; Jackson Immunoresearch Laboratories, West Grove, PA, USA) for 1 hour at room temperature. Nuclei were then stained using Hoechst 33342 (Molecular Probes, Life Technologies) and preparations were mounted in Q Path Safemount. The same protocol was used for immunocytochemistry on spheres but before fixation with PFA spheres in suspension were put on slides using cytospin centrifuge (Thermo Scientific) during 5 minutes à 40 rcf.

#### Immunohistochemistry

Slides of chick embryo sections were incubated for 1 hour with 10% normal donkey serum in PBS-T. Antibodies used were: rabbit anti-Tuj1 (1:2500; Covance) and mouse anti-HUMAN NUCLEI (1:500; Millipore). Primary antibodies diluted in PBS-0.3%Triton were then incubated overnight at 4°C. After 3 PBS washes, cells were incubated with Rhodamine Red X-conjugated secondary antibody (1:500; Jackson Immunoresearch Laboratories) and Fluorescein (FITC)-conjugated secondary antibody (1:500; Jackson Immunoresearch Laboratories) for 1 hour at room temperature. Nuclei were then stained using Hoechst 33342 (Molecular Probes) and slides were mounted in Q Path Safemount.

### Image acquisition and analysis

Image acquisition and analysis were either performed using a Zeiss AxioImager Z1 epifluorescence microscope (Zeiss, Zaventem, Belgium) coupled with FluoUp software; or using NIKON A1R hybrid resonant confocal microscope couple with NIS-Element software. The digitized images were adjusted for brightness and contrast using the NIH program ImageJ (Wayne Rasband, National Institute of Mental Health, Bethesda, MD, USA).

### Chemotaxis assay

In order to investigate migration phenomenon observed in chick embryo we developed chemotaxis assay using human cells from adipose tissue, bone marrow and skin and chemokines like stromal cell-derived factor 1 (SDF-1), SCF and Neurotrophin-3 (NT3). 12h before starting the experiment, human cells were cultivated in serum-free DMEM. In a first time ATSC, BMSC and SKSC were labeled with Cell Tracker Green (CTG) (Life Technologies) in serum-free DMEM. 25 000 cells were then placed on 5.2 mm-diameter filters (each containing 25 000 10 μm-pores) (ChemoTx, NeuroProbe, Gaithersburg, MD, USA). Below the filter, in a bottom chamber, we placed 30 μl of DMEM containing chemokines. The plate was incubated for 48h at 37°C. After incubation, non-migrating cells were removed from the top of the filter. Finally using fluorescence microscopy, we then used ImageJ software to quantify the percentage of filter area occupied by ATSC, BMSC or SKSC in response to different concentration of chemokine cocktail. Control condition contains on the bottom chamber DMEM alone and cells on the top compartment.

### Reverse transcription and quantitative polymerase chain reaction (RT-qPCR)

RNA from adherent cells and spheres was extracted using TRIzol reagent (Life Techonologies, Carlsbad CA, USA). RNA amount was quantified using NanoDrop spectrophotometer (Thermo Scientific). After DNase treatment (Promega, Madinson, WI, USA), cDNA synthesis was carried out using Moloney-murine leukemia virus (MMLV) Reverse transcriptase (Promega) and random hexamer primers (Promega), following the manufacturer's instructions. cDNA product was then use for real-time PCR in a LightCycler 480 (Roche, Belgium) using SYBR Green Master Mix (Eurogentec, Liege, Belgium). Gene expression levels were normalized using glyceraldehyde-3-phosphate dehydrogenase (GAPDH) housekeeping gene. Relative expression levels were obtained using ΔΔCt method. Amplifications were performed in triplicates, and on three biological replicates. Specific primers are listed in Table 1 (IDT, Leuven, Belgium).

**Table 1:** List of primers used in quantitative RT-PCR.

Gene	Sequence	
*AGGRECAN*	F	GAATCAACTGCTGCAGACCA
	R	CCACTGGTAGTCTTGGGGAT
*ALPL*	F	CCACGTCTTCACATTTGGTG
	R	AGACTGCGCCTGGTAGTTGT
*BARX2*	F	GAGTCAGAGACGGAACAGCC
	R	AGTCCCAGAGACTGAGCCAA
*BRN3A*	F	CGGTGTCCCAGGGCAAGAGC
	R	GCGACGGCGACGAGATGTGG
*FOXD3*	F	AGTGAAGCCGCCTTACTCGTACAT
	R	AGGAAGCTGCCGTTGTCGAACAT
GAPDH	F	GGCATGGACTGTGGTCATGAG
	R	TGCACCACCAACTGCTTAGC
*MBP*	F	TTAGCTGAATTCGCGTGTGG
	R	GAGGAAGTGAATGAGCCGGTTA
*MITF*	F	CTCGAGCTCATGGACTTTCC
	R	CCAGTTCCGAGGTTGTTGTT
*MSI1*	F	CGAGCTCGACTCCAAAACAAT
	R	AGCTTTCTTGCATTCCACCA
*NESTIN*	F	CAGCTGGCGCACCTCAAGATG
	R	AGGGAAGTTGGGCTCAGGACTGG
*NGN1*	F	CCCGGTGCCCAGGACGAAGAGCAG
	R	GGCCCGGGCAGACAGGGGACACA
*P0*	F	CAGTGAGAAGAAGGCCAAGG
	R	TTTTTGAGGCTGGTTCTGCT
*P75* ^*NTR*^	F	GTGGGACAGAGTCTGGGTGT
	R	AAGGAGGGGAGGTGATAGGA
*PAX3*	F	GCCAATCAACTGATGGCTTT
	R	CGTTCGAAGGAATAGTGCTT
*PMP22*	F	GAAGAAGGGGTTACGCTGTT
	R	CCTGAGGAAGAGGTGCTACA
*RUNX2*	F	TTTGCACTGGGTCATGTGTT
	R	TGGCTGCATTGAAAAGACTG
*S100β*	F	ACTACTGCCTGCCACGAGTT
	R	CCGTTAAAACAGCCTTTGGA
*SLUG*	F	CATCTTTGGGGCGAGTGAGTCC
	R	CCCCCGTGTGAGTTCTAATGTGTC
*SNAIL*	F	CGGCGCCGTCGTCCTTCT
	R	GGCCTGGCACTGGTATCTCTTCAC
*SOX9*	F	TACGACTACACCGACCACCA
	R	TTAGGATCATCTCGGCCATC
*SOX10*	F	ATGAACGCCTTCATGGTGTGGG
	R	CGCTTGTCACTTTCGTTCAGCAG
*TWIST*	F	ATCAAACTGGCCTGCAAAAC
	R	TGCATTTTACCATGGGTCCT
*TYRP2*	F	GGTTCCTTTCTTCCCTCCAG
	R	AACCAAAGCCACCAGTGTTC

### Statistical analysis

Data were analyzed using GraphPad InStat software (GraphPad Software, Inc., La Jolla, CA, USA, www.graphpad.com). Data are reported as mean ± standard error of mean (SEM), with n corresponding to the number of donors for each cell type. Level of statistical significance was set at p < 0.05. Statistical analysis for RT-qPCR results was performed on square-root transformed data.

## Results

### Characteristics of NCSC and MSC isolated from mouse adult bone marrow

**Fig 1:**
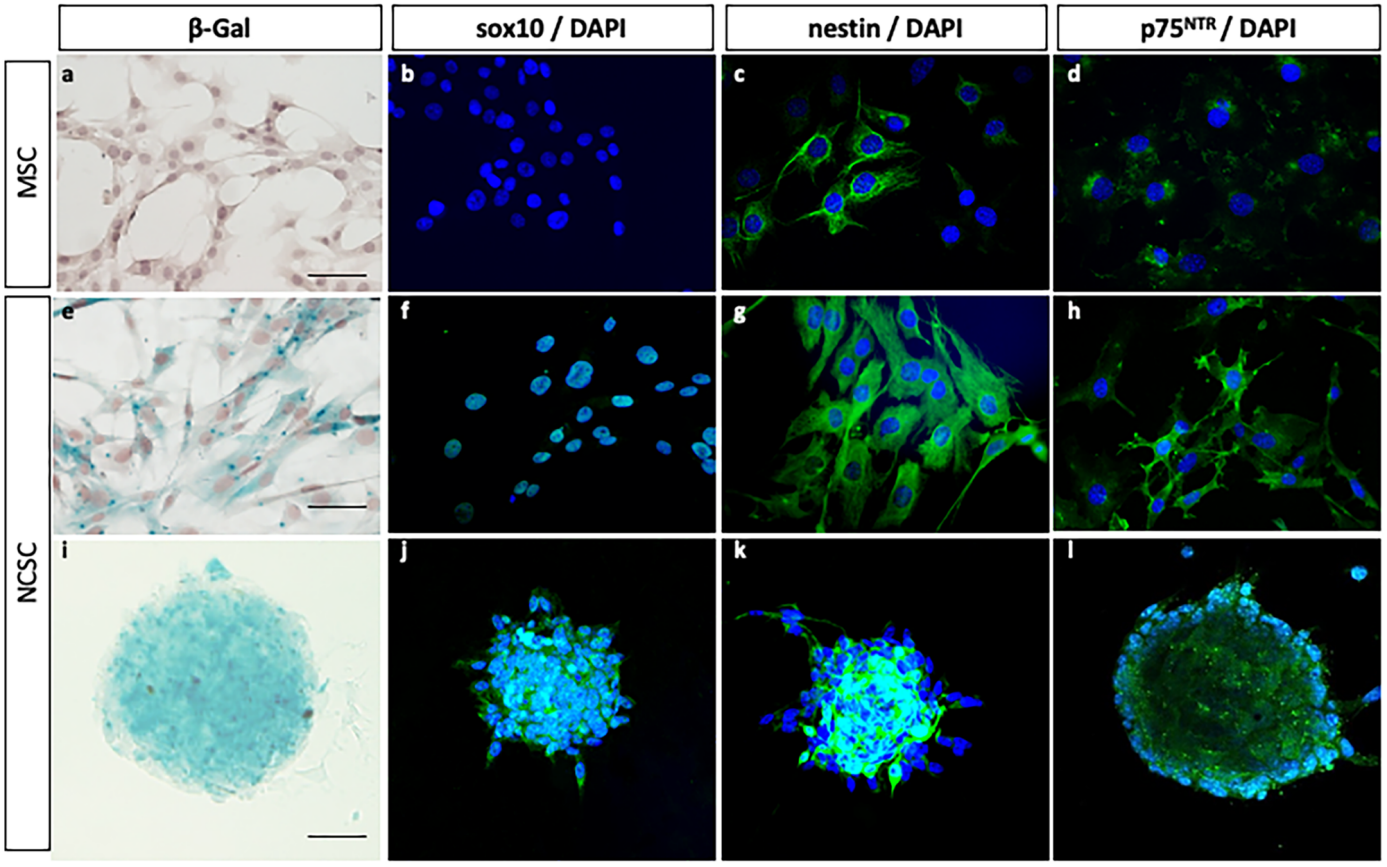
Characteristics of mouse NCSC and MSC from adult bone marrow. NCSC obtained from Wnt1-Cre/R26R-LacZ mice expressing *LacZ*. In adherent culture conditions, MSC did not express β-galactosidase (a) neither sox10 (b), but a small and constant proportion of cells slightly expressed nestin (c) and p75^NTR^ (d). NCSC expressed β-galactosidase (e), sox10 (f), Nestin (g) and p75^NTR^ (h). Finally, among these two populations, only NCSC were able to grow as spheres; spheres also express β-galactosidase (i), Sox10 (j), Nestin (k) and p75^NTR^ (l). (Scale bars = 40μm).

To isolate pure NCSC and MSC populations from bone marrow, we used a clonal selection approach [18 retracted in 68] in which bone marrow stromal cells from *Wnt1-Cre/R26R*-LacZ mice were individually seeded into 96-well plates (mean density of 0.7 cell per well). Each clone was then classified according to its embryonic origin, based on the beta-galactosidase expression. MSC were beta-galactosidase-negative, whereas NCSC were beta-galactosidase-positive (Fig 1a and 1e). After further characterization, we observed that NCSC were positive for transcription factor Sox10, the intermediate filament protein Nestin and the neurotrophin receptor p75^NTR^ (Fig 1f–1h). MSC were negative for sox10, weakly positive for p75^NTR^ and nestin (Fig 1b–1d). More interestingly, only NCSC were able to grow in suspension as spheres (Fig 1i–1l). Altogether, both NCSC specific marker expression and sphere-forming ability could be used as a tool to specifically identify NCSC in bone marrow. As genetic tracking markers are obviously non available in human, we decided to use those specific characteristics to assess the presence of NCSC in the human bone marrow.

### Characterization of human stem cells according to the International Society for Cell Therapy standard protocol, for mesenchymal stem cells

**Fig 2:**
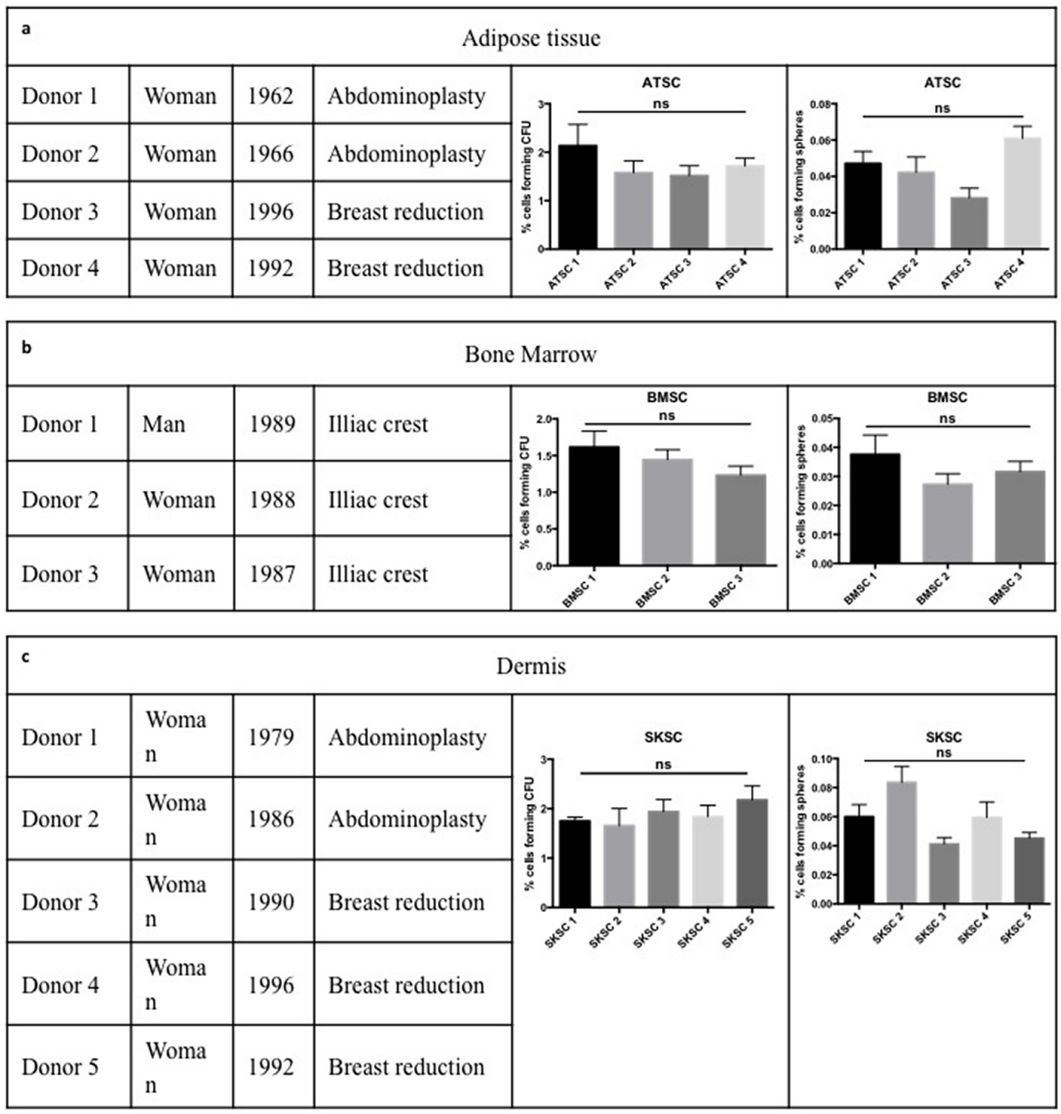
Cell origin and characterization. BMSC were obtained from iliac crest aspiration performed on three healthy donors (man and women) aged of more or less 20 years old (b). ATSC (a) and NCSC (c) were obtained using residual material form abdominoplasties or breast reductions performed on healthy women from 18 to 52 years old. We compared the ability to grow as sphere and the CFU potential between donors from the same cell type and we did not observed statistically significant differences between them. Statistical analysis: one way ANOVA followed by HSD post hoc test.

Since the embryonic origin of stromal stem cells is less evident to assess in human, we decided to use dermis (Skin derived stem cells, SKSC) and adipose tissue-derived stem cells (ATSC) as positive controls. Indeed, those two populations have already been described as heterogeneous populations containing neural crest derived cells [28–31,43]. SKSC and ATSC were isolated from healthy patients having breast reduction or abdominoplasty; bone marrow stromal cells (BMSC) were isolated from iliac crest aspiration, from healthy volunteers. BMSC donors were healthy men and women aged approximately 20 years old (Fig 2b). ATSC (Fig 2a) and SKSC (Fig 2c) were obtained from surgery residual material, from female donors aged from 18 to 52 years old. In order to check if donor gender and/or age could influence our results, we compared sphere forming ability and colony forming unit (CFU) potency between our different donors, in each cell type. No statistical significance was observed neither from sphere-forming nor CFU abilities in ATSC, BMSC and SKSC populations.

**Fig 3:**
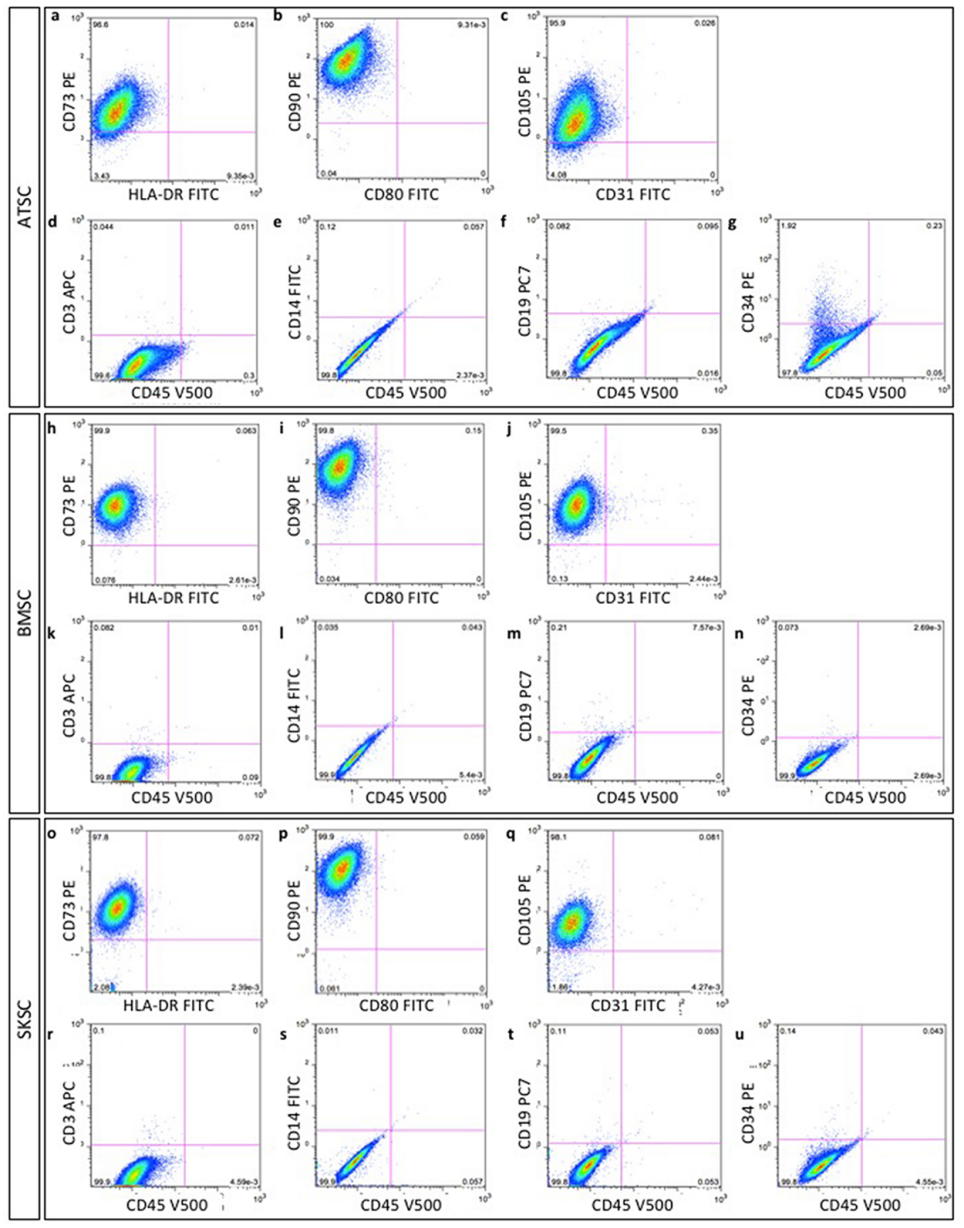
Characterization of human bone marrow, adipose tissue and dermis-derived stem cells, according to the International Society for Cell Therapy criteria. Mesenchymal and non-hematopoietic proteins expression were studied and quantified using flow cytometry phenotyping. Homogeneous populations of >95% of ATSC, BMSC and SKSC expressed mesenchymal markers: CD73 (a, h, o), CD90 (b, i, p) and CD105 (c, j, q) while the specific expression of hematopoietic markers was restricted to <5% of the cells: CD3 (d, k, r), CD14 (e, l, s), CD19 (f, m, t), CD34 (g, n, u), CD45 (d-g, k-n, r-u), HLA-DR (a, h, o), CD80 (b, i, p) and CD31 (c, j, q).

We cultivated ATSC, BMSC and SKSC in adherent culture conditions, for a maximum of 8 passages. According to the recommendations issued by International Society for Cellular Therapy guidelines (ISCT) [44], using flow cytometry phenotyping, we observed that more than 99% of cells were positive for CD73, CD90, CD105 (ATSC Fig 3a–3c; BMSC Fig 3h–3j and SKSC Fig 3o–3q), but negative for hematopoietic lineage markers such as CD3, CD14, CD19, CD34, CD45, HLA-DR, CD31 and CD80 (ATSC Fig 3a to 3g; BMSC Fig 3h to 3n and SKSC Fig 3o to 3u). As we could expect, it appeared that those guidelines efficiently discriminate hematopoietic from non-hematopoietic cells, but would not be useful to discriminate neural crest-derived cells from the entire population.

### Comparison of mouse and human cells with regard to NCSC-related characteristics

**Fig 4:**
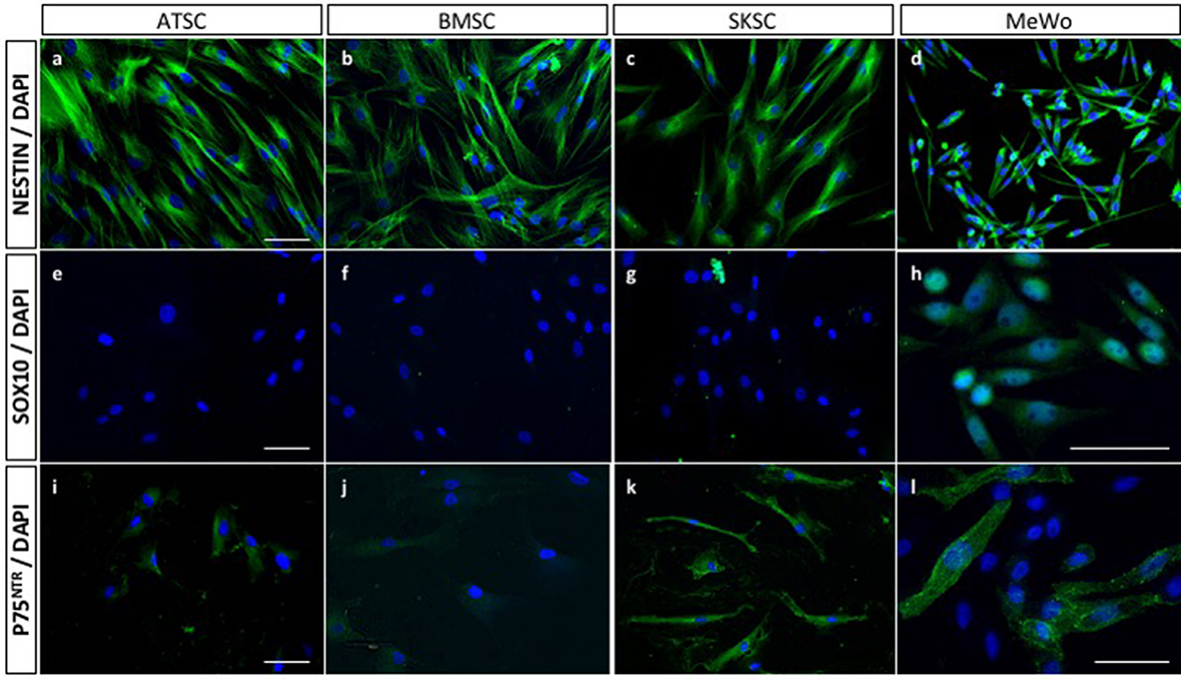
Mouse NCSC-related characteristics in human cells. Similarly to immunostainings performed in mouse (Fig 1), human ATSC, BMSC, SKSC expressed NESTIN (a-c), did not express SOX10 (e-g). ATSC and BMSC express weakly P75^NTR^ (i-j) in comparison to SKSC (k). We also used a cell line derived from human malignant melanoma cells (MeWo), which was a positive control for NC identity (d, h, l). (Scale bars = 20μm).

As previously described for mouse NCSC and MSC (Fig 1), we characterized the three human cell types (BMSC, ATSC and SKSC) using NESTIN, SOX10 and P75^NTR^ expression. Surprisingly, all three populations were homogeneously positive for NESTIN (Fig 4a to 4c), but negative for SOX10 (Fig 4e to 4g). The only difference we observed concerned P75^NTR^ expression, since SKSC were P75^NTR^-positive (Fig 4k), while BMSC and ATSC were weakly P75^NTR^ positive (Fig 4i to 4k). We also performed these immunostainings using a positive control for NC identity (MeWo cell line) and as expected NESTIN (Fig 4d), SOX10 (Fig 4h) and P75^NTR^ (Fig 4l) were expressed.

**Fig 5:**
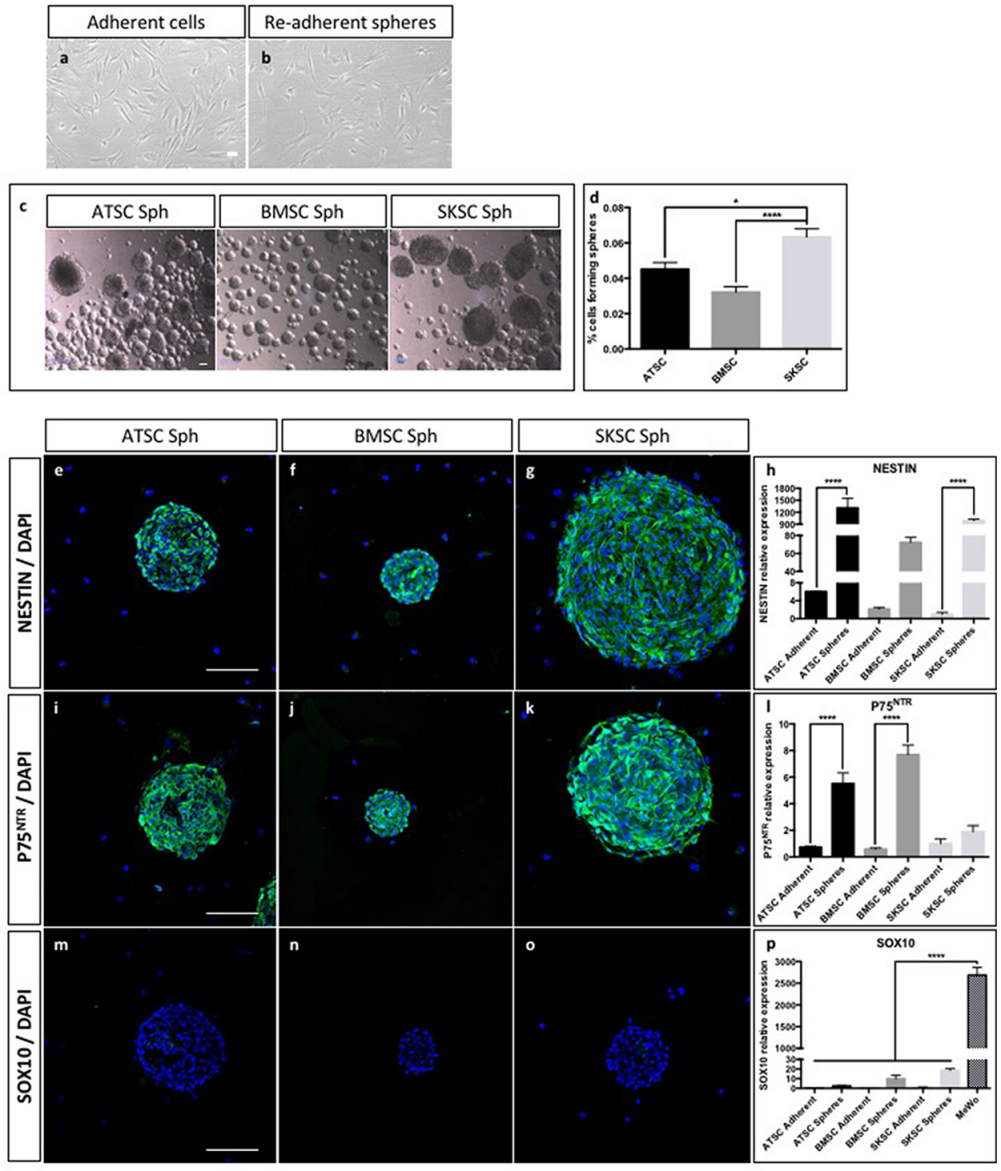
Sphere forming ability and properties of human cells. The majority of ATSC, BMSC and SKSC were able to grow as sphere, in specific culture medium. Using light and transmission microscope, we were not able to distinguish adherent cells from re-adherent sphere-derived cells (a and b). Spheres presented a diameter between 80 and 350 microns (c). We quantified the ability to each cell type to grow as spheres, after 12 days of culture compare to the initial number of plated cells (75 000 cells in 25 cm^2^ T-flask). SKSC presented a significantly higher ability to grow as spheres compared to BMSC and ATSC (respectively p<0.0001 and p<0.05 one way ANOVA followed by HSD post hoc test) (d). Similar immunostaining characterizations as performed in Fig 4 were performed on sphere-derived cells. In this case, human sphere-derived ATSC, BMSC and SKSC expressed NESTIN (e-h) and did not express SOX10 (m-p). However, unlike adherent cells, sphere-derived ATSC, BMSC and SKSC expressed P75^NTR^ (i-l). Quantitative RT-PCR were performed on adherent and sphere-derived cells and confirmed immunological observation made at mRNA level (h, l, p). Data were normalized to SKSC adherent cells expression level which was set as 1. Statistical analysis: one way ANOVA followed by HSD post hoc test. **** means p<0.0001. (Scale bars = 50μm).

We then decided to analyze their ability to grow as spheres, and tested a sphere-forming protocol on those three human cell types. As a reminder, we previously described that only NCSC isolated from mouse adult bone marrow were able to grow as sphere (Fig 1) compared to adult bone marrow derived MSC. This property could therefore be used as a tool to discriminate mouse NCSC from mouse MSC. Using light and transmission microscope we were not able to differentiate adherent cells from adherent cells obtained after re-adherence from spheres (Fig 5a and 5b). As observed on Fig 5c, all cell types were able to grow as spheres. Those spheres reached a diameter from 80 to 350 μm. We also assessed the sphere-forming rate (Fig 5d) and it appeared that SKSC presented a significantly higher ability to grow as spheres compared to BMSC (p<0.0001) and ATSC (p<0.05). The proportion of cells growing as spheres was based on the total number of obtained spheres, after 12 days of culture, compare to the number of initially plated cells. We further characterized those spheres, which appeared to be NESTIN-positive (Fig 5e to 5h), p75^NTR^-positive (Fig 5i to 5l) and SOX10-negative (Fig 5m to 5p). These observations were confirmed at the mRNA level using quantitative RT-PCR. Indeed, *NESTIN* (Fig 5h) and *P75*^*NTR*^ (Fig 5l) were highly expressed in spheres compare to adherent cells (a difference was observed in ATSC for both genes (p<0.0001), in SKSC for *NESTIN* (p<0.0001) and in BMSC for *P75*^*NTR*^ (p<0.0001). As observed on Fig 5p, we detected a weak expression level for *SOX10* in the different culture conditions, whereas we never detected any signal by immunofluorescence. However, the expression level was really weak (p<0.0001) compared to the expression level observed in MeWo cells. This last was human malignant melanoma cells, and was the only cell type expressing SOX10 at the protein level (Fig 4h). Altogether, it appeared that mouse MSC and NCSC features were not fully transposable to their human counterparts, and further characterization was therefore required in order to attest the potential presence of NCSC in human adult bone marrow.

### Differentiation abilities of human bone marrow, adipose tissue and dermis-derived stem cells

**Fig 6:**
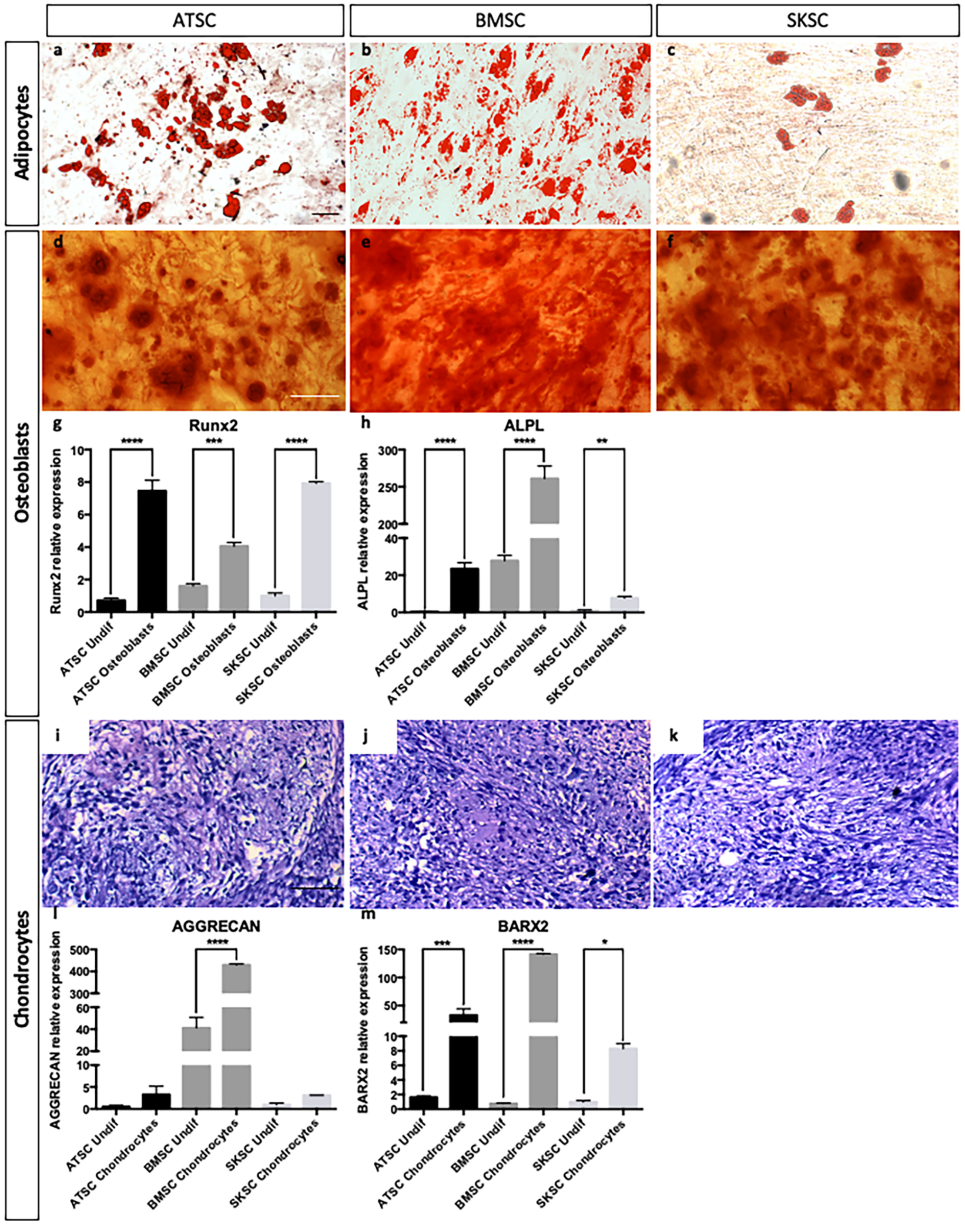
Differentiation abilities of human bone marrow, adipose tissue and dermis-derived stem cells. ATSC, BMSC and SKSC were able to differentiate into adipocytes (lipids vacuoles stained with Oil Red-O—a-c), osteoblast (calcium deposits stained with Alizarin Red—d-f), chondrocytes (chondrocyte matrix stained with Alcian Blue—i-k). Osteoblastic and chondrogenic differentiations were also assessed by quantitative RT-PCR based on *RUNX2* (g), *ALPL* (h), *AGGRECAN* (j) and *BARX2* (k) expression levels. Data were normalized to SKSC undifferentiated cells expression level set as 1. Statistical analysis: one way ANOVA followed by HSD post hoc test. * means p<0.05, *** means p<0.0005, **** means p<0.0001. (Scale bars = 50μm).

In order to determine if human BMSC, ATSC and SKSC are able to differentiate into classical mesenchymal derivatives such as adipocytes, osteocytes and chondrocytes, but also into neural crest-derived cells like Schwann cells, melanocytes, and neurons, we placed those cells in different specific culture conditions in order to induce their differentiation (as described in experimental procedures section). As expected, the three cell types were able to differentiate into adipocytes (Oil-Red-O labeling, Fig 6a–6c), osteoblasts (Alizarin-Red labeling, Fig 6d–6f) and chondrocytes (Alcian Blue, Fig 6i–6k). Concerning osteoblasts and chondrocytes, we also performed quantitative RT-PCR for *RUNX2*, *ALPL*, *AGGRECAN* and *BARX2* (Fig 6g, 6h, 6l and 6m) to validate their differentiated stage compare to undifferentiated cells.

**Fig 7:**
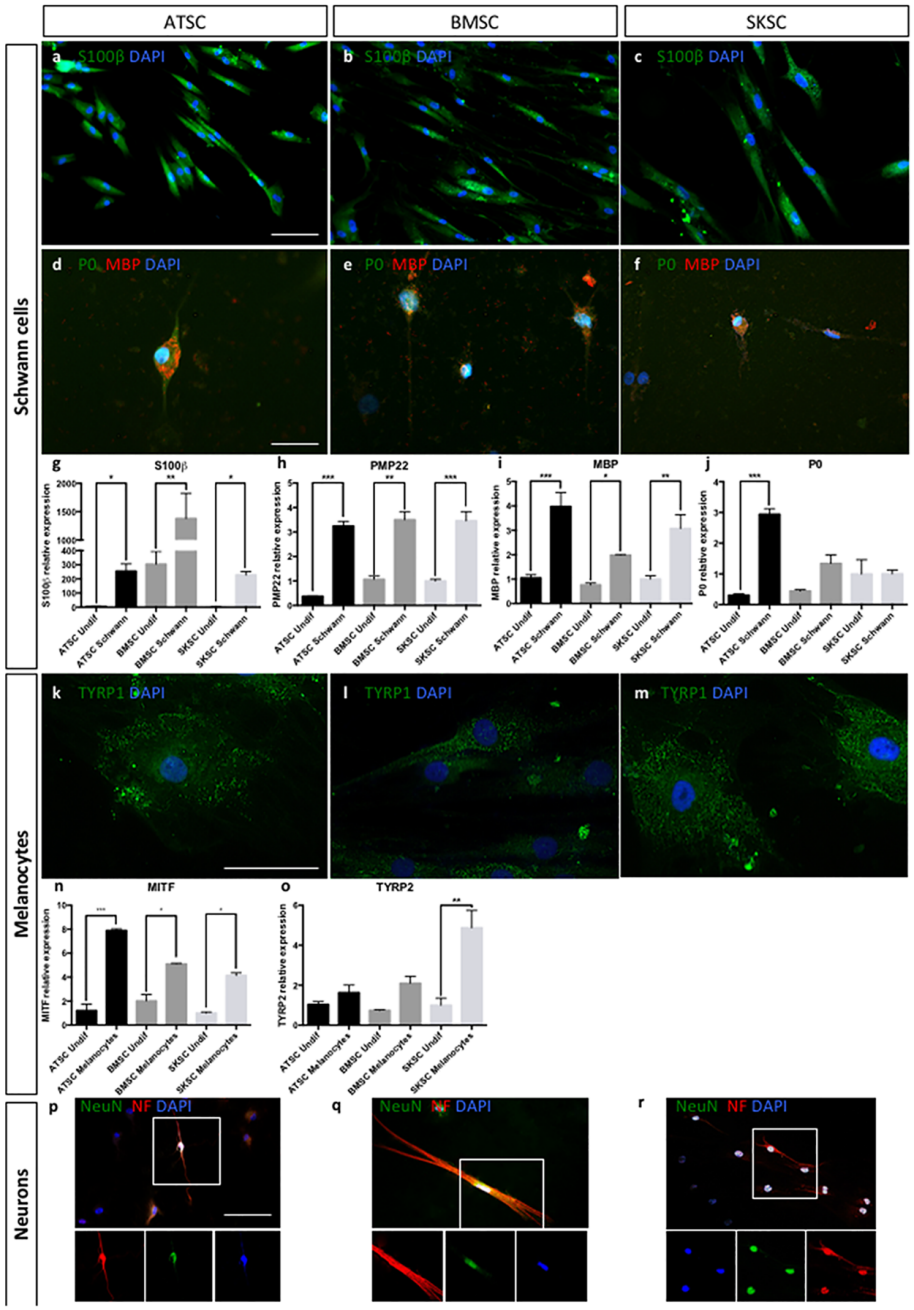
Differentiation abilities of human bone marrow, adipose tissue and dermis-derived cells. ATSC, BMSC and SKSC were able to differentiate into Schwan cells as the majority of the cells were stained with anti-S100β antibody (a-c) and a small amount of S100β^+^ cells was also stained with anti-MBP (red) and anti-P0 (green) antibodies (d-f). Quantitative RT-PCR for *S100β*, *PMP22*, *MBP* and *P0* confirmed the results obtained by immunofluorescences, at mRNA level (g-j). The three cell types were also able to differentiate into melanocytes stained with anti-TYRP1 antibody (k-m), which was confirmed at mRNA level based on quantitative RT-PCR for *MITF* (n) and *TYRP2* (o). Finally, neuronal differentiation was assessed using anti-NeuN (green) and anti-Neurofilament (green) antibodies (p-r). Data were normalized using SKSC undifferentiated cells expression level set as 1. Statistical analysis: one way ANOVA followed by HSD post hoc test. * means p<0.05, ** means p<0.005, *** means p<0.0005. (Scale bars a, b, c, d, e, f, p, q and r = 50μm; Scale bars k, l,and m 20μm).

Schwann cell differentiation was attested by S100-β (Fig 7a to 7c), MBP (red) and P0 (green) labeling (Fig 7d to 7f), as well as by quantitative RT-PCR for *S100-β*, *PMP22*, *MBP* and *P0* genes (Fig 7g to 7j). Interestingly, S100-β was detected in a large amount of cells (observed at protein level, but also with the higher mRNA expression level compared to other genes) whereas only a few S100-β positive cells were also MBP and P0 positives. Indeed, these two markers were specific for Schwann cells at later differentiating stages. These results were confirmed at the mRNA level (Fig 7g to 7j). Melanocyte differentiation was attested by TYRP1 labeling (Fig 7k and 7l), as well as by quantitative RT-PCR for *MITF* and *TYRP2* (Fig 7n and 7o). Quantitative RT-PCR for *PMP22*, *P0* and *MBP* (Fig 7h to 7j) and *MITF* and *TYRP2* (Fig 7n and 7o) confirmed observations previously performed at the protein level, concerning their differentiating potential. Neuronal differentiation was attested by NeuN (green) and Neurofilament (red) expression (Fig 7p to 7r). Altogether, it appeared that those three human cell populations (ATSC, BMSC and SKSC) were able to differentiate into mature mesenchymal cell types (adipocytes, osteoblasts and chondrocytes) and neural crest-derived cells (Schwann cells, melanocytes and neurons).

### Transcriptomic and proteomic characterization using NCSC-related markers

**Fig 8:**
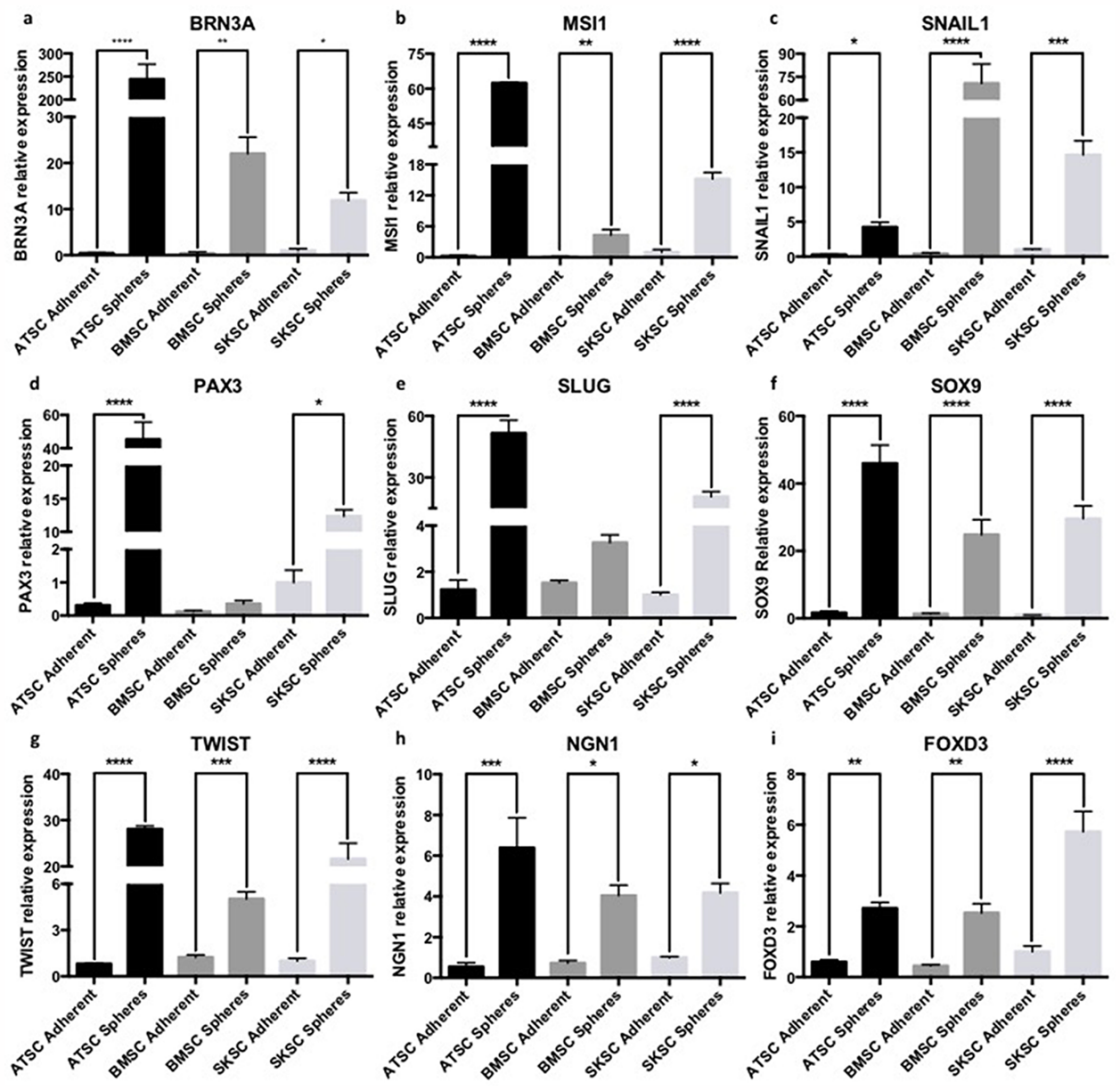
Quantitative RT-PCR characterization using stem cells and NCCs-related markers. The expression of neural crest and stem cells genes was studied here by quantitative RT-PCR. ATSC, BMSC and SKSC either in adherent or in sphere culture condition expressed NCSC markers such as *BRN3A*, *FOXD3*, *NGN1*, *PAX3*, *SLUG*, *SNAIL1*, *SOX9* and *TWIST* and neural differentiation marker such as *MSI1*. Data were normalized using SKSC adherent cell expression level set as 1. Statistical analysis: one way ANOVA followed by HSD post hoc test. * means p<0.05, ** means p<0.005, *** means p<0.0005, **** means p<0.0001.

**Fig 9:**
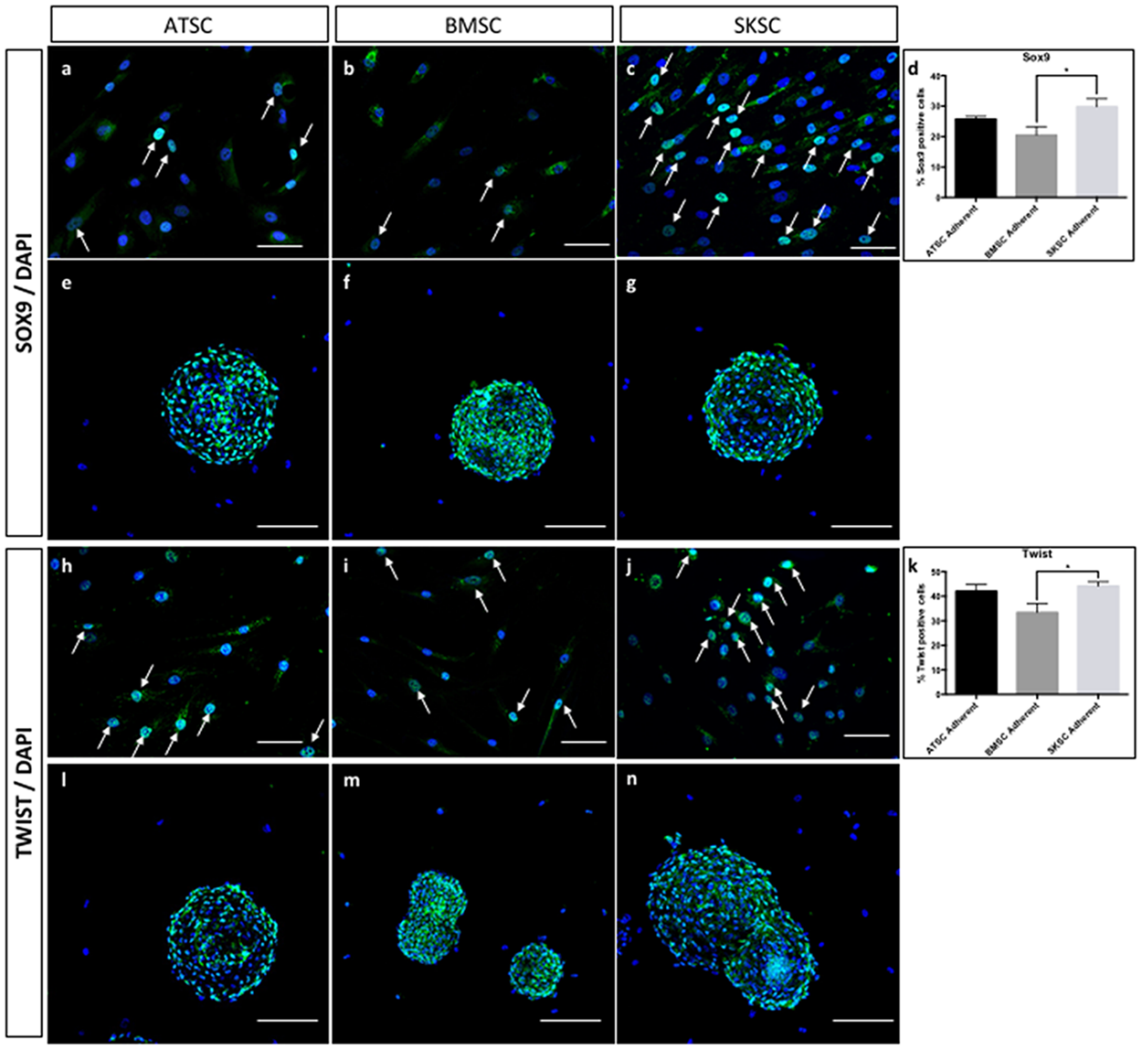
Expression of SOX9 and TWIST by ATSC, BMSC and SKSC. The SOX9 and TWIST expression which were previously analyzed at mRNA level, were now studied at protein level. It appeared that 25.7% (±0.9%) of adherent ATSC, 20.5% (±2.7%) of adherent BMSC and 29.8% (±2.6%) of adherent SKSC expressed SOX9. Similar results were obtained concerning TWIST: 42% (±2.7%) of adherent ATSC, 33,4% (±3.6%) of adherent BMSC and 44,2% (±1.7%) of adherent SKSC whereas both proteins were expressed by more than 80% of the cells, in spheres. Arrows indicated either SOX9 or TWIST positive cells in adherent culture condition. Statistical analysis: one way ANOVA followed by HSD post hoc test. * means p<0.05. White arrowheads indicate the positive cells. (Scale bars for a, b, c, h, i, and j = 40μm; Scale bars for e, f, g, l, m, n = 50μm).

The next step of this study was to characterize the expression profile of several markers that are classically expressed by NCSC. We first analyzed the expression of the following genes, using quantitative RT-PCR: *FOXD3*, *PAX3*, *SLUG*, *SNAIL1*, *SOX9* and *TWIST*. Those genes are involved as neural crest specifiers and/or as neural plate border specifiers. Indeed, *NGN1* is involved in neural differentiation; *BRN3A* is associated with PNS development; and finally *MSI1* is linked with neural precursors. As we can observe in Fig 8, most of these transcripts were overexpressed in spheres compared to adherent cells in the three cell types (ATSC, BMSC and SKSC). We confirmed those results at the protein level for SOX9 (Fig 9a to 9g) and TWIST (Fig 9h to 9n). Interestingly, in adherent cells, we observed heterogeneous populations for several markers: 25.7% (±0.9%) of adherent ATSC, 20.5% (±2.7%) of adherent BMSC and 29.8% (±2.6%) of adherent SKSC expressed SOX9 (Fig 9d) and 42% (±2.7%) of adherent ATSC, 33,4% (±3.6%) of adherent BMSC and 44,2% (±1.7%) expressed TWIST (Fig 9k). Furthermore, more than 80% of the cells, in spheres, expressed SOX9 and TWIST. Altogether, those results reinforce the hypothesis that neural crest-derived cells are present in human dermis, bone marrow and adipose tissue. They also suggest that spheres are an enriched population of neural crest cells (NCC).

### Characterization of bone marrow stem cell migrating abilities when injected into chicken embryos

**Fig 10:**
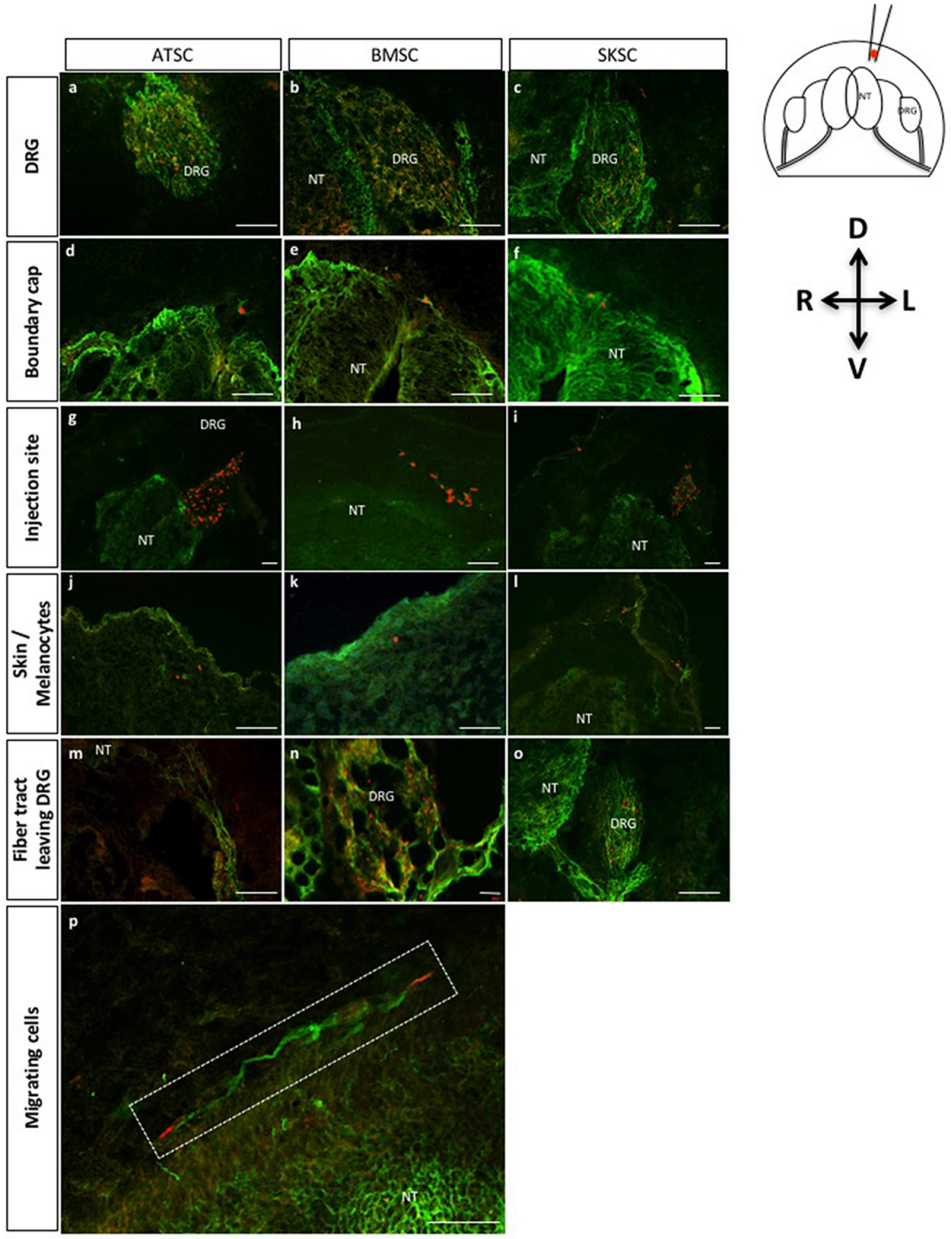
Characterization of adherent bone marrow, adipose tissue and dermis-derived stem cell migration abilities when injected into chick embryos: Localization of migrating cells. Fig 10 represents transversal (a-o) and longitudinal (p) sections of adherent cells injected into HHSt18 chick embryos. Human stem cells derived from adipose tissue, bone marrow and dermis were localized into chick DRG (a-c), boundary cap of the NT (d-f), injection site (g-i), skin or more precisely melanocyte region (j-l) and finally the fiber track leaving the DRG (m-o). Fig 10p presents longitudinal section with magnification on migrating cells along the neural tube. (Scale bars = 50μm, Green: TUJ1 labeling, Red: human nuclei labeling).

To finalize our characterization, we decided to test the migrating properties of those three cell types, once injected into chicken embryos. Indeed, it has been well described [12,45,46] that when injected into chicken embryos at a specific stage, NCSC are able to migrate and follow chicken NCSC migration pathways. More precisely, this experiment consisted in the injection of a small amount of cells into the dorso-lateral aspect of the thoraco-lumbar region of Hamburger and Hamilton stage 18 (HHst18) chicken embryos (corresponding to the early period of NC migration from the dorsal NT at the trunk level). We therefore injected around 300 adherent cells or one sphere into HHst18 chicken embryos. The embryos were sacrificed 72 hours post-injection. Injected cells and their derivatives were traced using a specific marker for human cells (i.e. anti-HUMAN NUCLEI marker). At this developmental stage, trunk NCSC could give rise to two main populations [47]: 1) sensory neurons and glial cells of the dorsal root ganglion (DRG) and associated nerves; 2) melanocytes of the skin. At this stage, the boundary cap cells are known to be NC-derived cells forming clusters (that will migrate later) at the surface of the NT [48]. Some injected cells were observed in the fiber tracts leaving DRG, which also include NCSC. In this context, the three cell types (adherent ATSC, BMSC and SKSC) were localized into the peripheral neural crest targets as described thereafter: most of the cells were migrating to the DRG (Fig 10a to 10c; S1a to S1c Fig), but some were also found in the area of the boundary cap (Fig 10d to 10f, S1d to S1f Fig), in the dermal layer of the skin (a few cells were observed at the surface of the embryo corresponding to melanocyte migration territory) (Fig 10j to 10l; S1j to S1l Fig) and finally in nerve fiber tracts leaving the DRG (Fig 10m to 10o, S1m to S1o Fig). Cells were also found in the injection site illustrating that all cells were not able to migrate (Fig 10g to 10I, S1g to S1l Fig), attesting the specificity of the migration. Finally, in Fig 10p (S1p Fig) longitudinal section presented at higher magnification migrating cells on the top of the NT. Similar observations were made after sphere injections.

**Fig 11:**
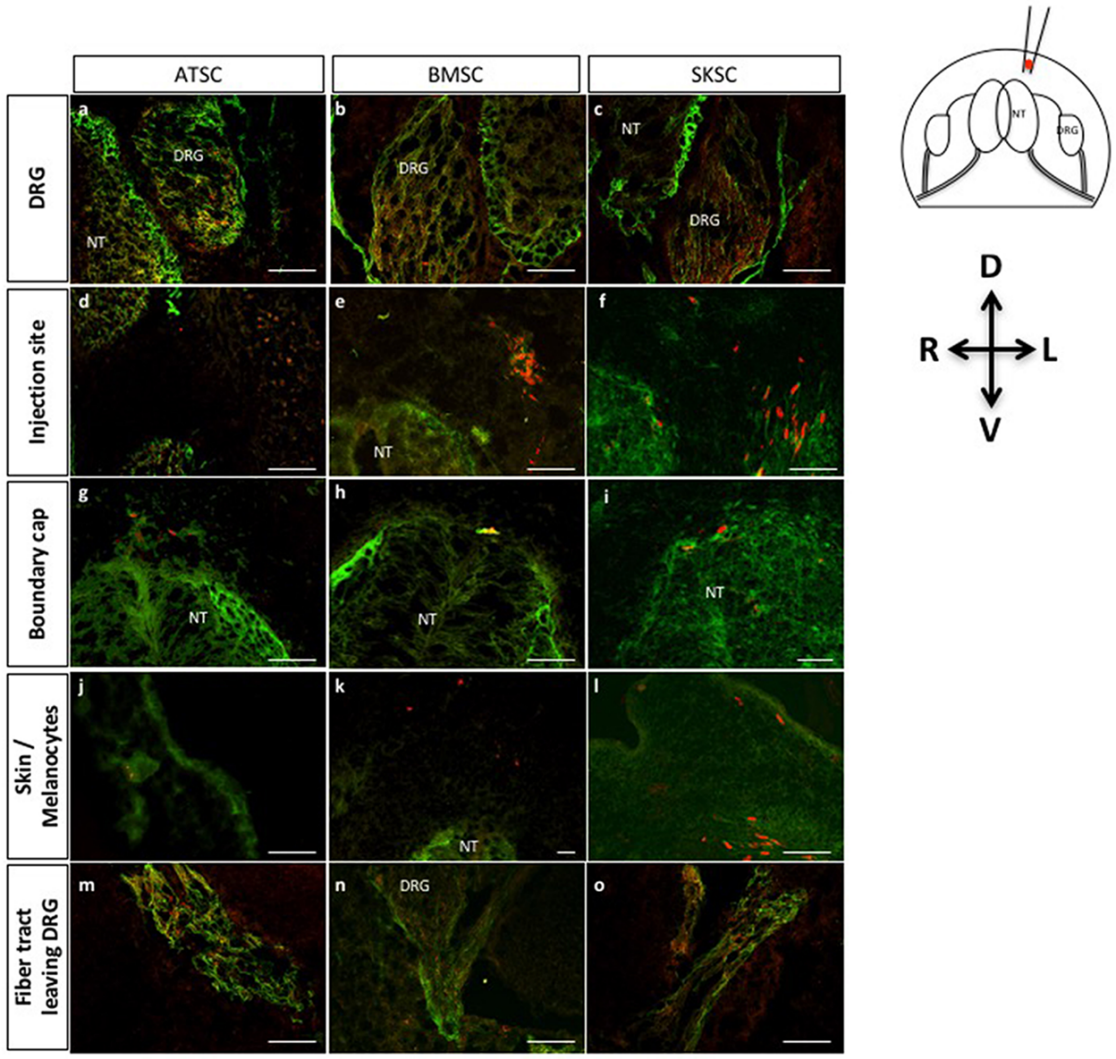
Characterization of migration abilities of bone marrow, adipose and dermis-derived spheres when injected in chick embryos: Localization of migrating cells. Fig 11 represents transversal sections of spheres injected into HHSt18 chick embryos. Cells originating from human adipose, bone marrow and dermis-derived spheres were localized into chick DRG (a-c), boundary cap of the NT (d-f), injection site (g-i), skin or more precisely melanocyte region (j-l) and finally the fiber track leaving the DRG (m-o). (Scale bars = 50μm, Green: TUJ1 labeling, Red: human nuclei labeling).

Here again, sphere-derived cells were detected in the DRG (Fig 11a to 11c, S2a to S2c Fig), in the area of the boundary cap (Fig 11d to 11f, S2d to S2f Fig), in the injection site (Fig 11g to 11i, S2g to S2i Fig), in migration territory of the melanocytes (Fig 11j to 11l, S2j to S2l Fig) and in the nerve fiber tracts leaving the DRG (Fig 11m to 11o, S2m to S2o Fig). Specificity of the migration was addressed by the injection of iPS-derived NSC (S3 Fig). In those conditions, we observed that iPS-derived NSC were also able to migrate to the DRG, but they also colonized the neural tube which was never observed with ATSC, BMSC or SKSC.

**Fig 12:**
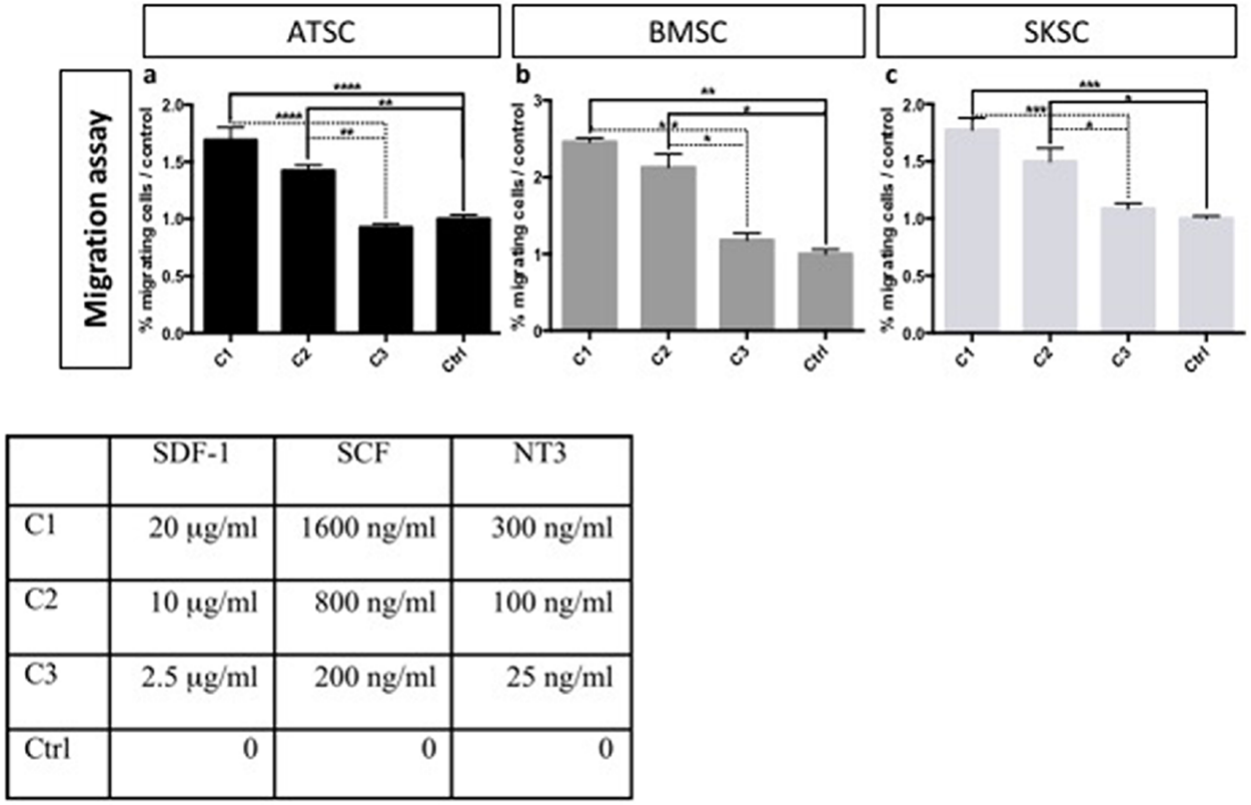
Chemotaxis assay on human adherent ATSC, BMSC and SKSC. Chemotaxis assay were build to confirmed migration abilities observed in chick embryos. We placed ATSC, BMSC or SKSC on top of a 10 μm-filter, with cocktail of SDF-1, SCF and NT3 in the bottom chamber. After 48 hours, migration rate was evaluated by the quantification of the filter area occupied by human cells. Passive migration was observed and quantified in control condition (only serum-free DMEM in the bottom chamber) and this value was used to normalize the results obtained with different cocktail concentrations (Table). Statistical analysis: (n = 3), one way ANOVA followed by HSD post hoc test. * means p<0.05, ** means p<0.005, *** means p<0.0005, **** means p<0.0001.

Altogether, we conclude that a subset of BMSC, ATSC and SKSC was able to migrate along neural crest migration pathways towards tissues classically colonized by neural crest derivatives. We also confirmed those migrating properties by performing an *in vitro* chemotaxis assay. Chemokine cocktail made of SDF-1, SCF and NT3 was used to attract adherent ATSC, BMSC and SKSC in order to mimic chicken embryo environment. We observed that human cells (from the three tissue origins) were responsive to this cocktail and migrate through the filter (Fig 12a to 12c).

## Discussion

The NC is a transient ephemeral structure containing cells that share the unique property, in vertebrates, to migrate, invade multiple tissues and give rise to diverse mature cell lineages. Most NC development knowledge derived from studies performed on chicken and zebrafish. It is obvious that human NC information is more difficult to obtain because of its transient nature and the limited availability of human fetal cells. In mouse, adult post-migratory NCSC were identified in several locations like gut, cornea, heart, bone marrow, adipose tissue and skin [49]. In human, the confirmation of the presence of neural crest cells in adult tissues is less obvious and mainly based on their expression profile, associated with their differentiating capacity and their ability to follow migration pathways when injected in chicken embryos. In those conditions, adult human NCSC have been identified in skin [28–32], hair follicle [30,31], in dental pulp [33–36], periodontal ligament [37,38], in cornea [39], inferior turbinate [40], olfactory or oral mucosa [41,42] and in adipose tissue [43].

In this study, we analyzed the putative presence of neural crest-derived cells in adult human bone marrow (BMSC). We compared human bone marrow cells with human adipose tissue (ATSC) and dermis (SKSC), two tissues already described as potential sources of neural crest-derived cells. Those three cell types proliferated in the same culture conditions. All cell types were CD73^+^, CD90^+^, CD105^+^ and NESTIN^+^. Moreover, all three populations were heterogeneous and showed some positivity for *BRN3A*, *FOXD3*, *MSI1*, *NGN1*, *PAX3*, *SLUG*, *SNAIL1*, *SOX2*, *SOX9*, and *TWIST*. Finally, sphere-derived cells showed a higher expression level of those genes, suggesting a potential enrichment in neural crest derived cells, similarly to our observations for mouse bone marrow derived NCSC. The Table 2 represents sphere forming ability and relative expression level of the three human populations studied here. Our results are in accordance with Abe and collaborators studies focusing on human putative NCSC isolated from human apical dental pulp [34] or even oral mucosa stroma [42]. Indeed, in these two studies it has been shown that: 1) 0.023% of human oral mucosa stromal cells are able to grow as spheres. 2) Neurospheres, obtained from dental pulp or oral mucosa, were expressing NESTIN, MSI1, P75^NTR^, SNAIL1, SLUG and SOX9.

**Table 2:** Recapitulative table of the ability of human cells from adipose tissue, bone marrow and dermis to grow as spheres and express NCSC markers.

		ATSC		BMSC		SKSC	
		Adherent cells	Spheres	Adherent cells	Spheres	Adherent cells	Spheres
Sphere forming ability		0.045% ±0.004		0.032% ±0.003		0.063% ±0.005	
Relative expression level	*BRN3A*	1	610	1	58	1	12
	*FOXD3*	1	5	1	5	1	6
	*MSI1*	1	310	1	25	1	15
	*NESTIN*	1	218	1	36	1	990
	*NGN1*	1	12	1	5,5	1	4
	*P75* ^*NTR*^	1	6,5	1	14	1	2
	*PAX3*	1	150	1	3	1	12
	*SLUG*	1	51	1	3	1	21
	*SNAIL1*	1	14	1	177	1	15
	*SOX9*	1	46	1	25	1	30
	*TWIST*	1	35	1	5	1	22

Even if it is well accepted that sox10 transcription factor is a classical NCSC marker for mouse embryonic and adult cells, in human adult cells, SOX10 expression is not systematically observed. Indeed, in Fournier *et al*., 2016, these authors described human gingival NCSC. Those cells are NESTIN, SNAIL1, TWIST, PAX3, SOX9 and FOXD3 positive and able to generate neurospheres [50]. Abe *et al*., 2016 described sphere-derived multipotent progenitor cells obtained from human oral mucosa, that were enriched in NCSC [42]. In that study, the authors characterized those cells as NESTIN, CD44, SLUG, SNAI1, MSX1, HES1, and SOX9 positive, but not SOX10. Hara *et al*., 2014 described potential NCSC isolated from human corneal endothelial progenitor cells that were P75^NTR^, SOX9, and AP-2β positive [39]. *Bueno et al*., 2013 described human adult periodontal ligament-derived cells that expressed stem cell markers (OCT3/4, NESTIN, SOX2, and MUSASHI-1) and a subset of NC markers (SLUG, P75^NTR^, TWIST, and SOX9) [37]. Hill *et al*., 2012 described multipotent NCSC from established adult human dermal cultures. Those NC cells were SOX2, SOX9, OCT4, NANOG, NESTIN, PAX3, SLUG, SNAI1, DERMO1 and P75^NTR^ positive [31]. Finally and maybe more interestingly, Krejci *et al*., 2010 demonstrated that NCSC from adult human hair follicles expressed SOX10 and NESTIN during emigration from follicles [51]. Cultivated cells were then positive for BMP4, SNAI1, SLUG, SOX9 and TWIST, suggesting that they lose their SOX10 expression after emigration. On the opposite, Pelaez *et al*., 2013 described human adult NCSC isolated from periodontal ligament [52], those Cx43^+^ periodontal ligament stem cells expressed pluripotency-associated transcription factors OCT4, NANOG, and SOX2, as well as NC-specific markers SOX10, P75^NTR^, and NESTIN. Similarly, Hauser *et al*., 2012 isolated NCSC from adult human inferior turbinate. In that study, the authors demonstrated that those cells were positive for the neural crest markers SOX9, SOX10, P75^NTR^, and SLUG [40].

The lack of SOX10 expression in our putative adult human bone marrow neural crest derived cells was surprising, as SOX10 transcription factor is commonly used as a NC marker. However, several facts could justify the absence of SOX10 expression. 1) SOX10 expression is mainly described in NC cells at the time of their emigration and is essential for their self-renewal and survival [49]. After reaching their final destination, SOX10 expression may disappear, depending on their environmental condition [53]. 2) Adult rodent bone marrow NCSC were systematically sox10^+^, while adult human bone marrow NCSC appeared to be SOX10 negative. This non-transposability of mouse results to human has already been described for NESTIN. Indeed, in mouse, it has been demonstrated that most NESTIN-positive cells from adult bone marrow are neural crest derived cells [18 retracted in 68, 54]. However, in human, all bone marrow stromal cells (including MSC and NCSC) are NESTIN^+^ [55,56]. 3) As in many cell lineages and tissues, SOX10 and SOX9 are functionally redundant [57]. It is therefore possible that in human adult bone marrow NCSC, SOX9 expression could substitute for SOX10 expression when cells have migrated and reached their final locations. Similarly, it has been demonstrated that, in some conditions, SOX10 expression could be down-regulated by SOX9 [58]. Indeed, those authors demonstrated that SOX9 was able to interact with SOX10 promoter and regulated its level of expression.

The phenotypic characterization of ATSC, BMSC and SKSC was followed by the assessment of their ability to differentiate in neural crest-derived mature cell types and to follow the neural crest migration track when injected into chicken embryos. In this last experiment, all cell types were able to migrate and follow endogenous neural crest migration pathways, as all cell types were localized in dorsal root ganglia, in skin, and in boundary cap, but none of them were observed in the neural tube or any specific location of neural cell types attesting of the specificity of the migration.

**Fig 13:**
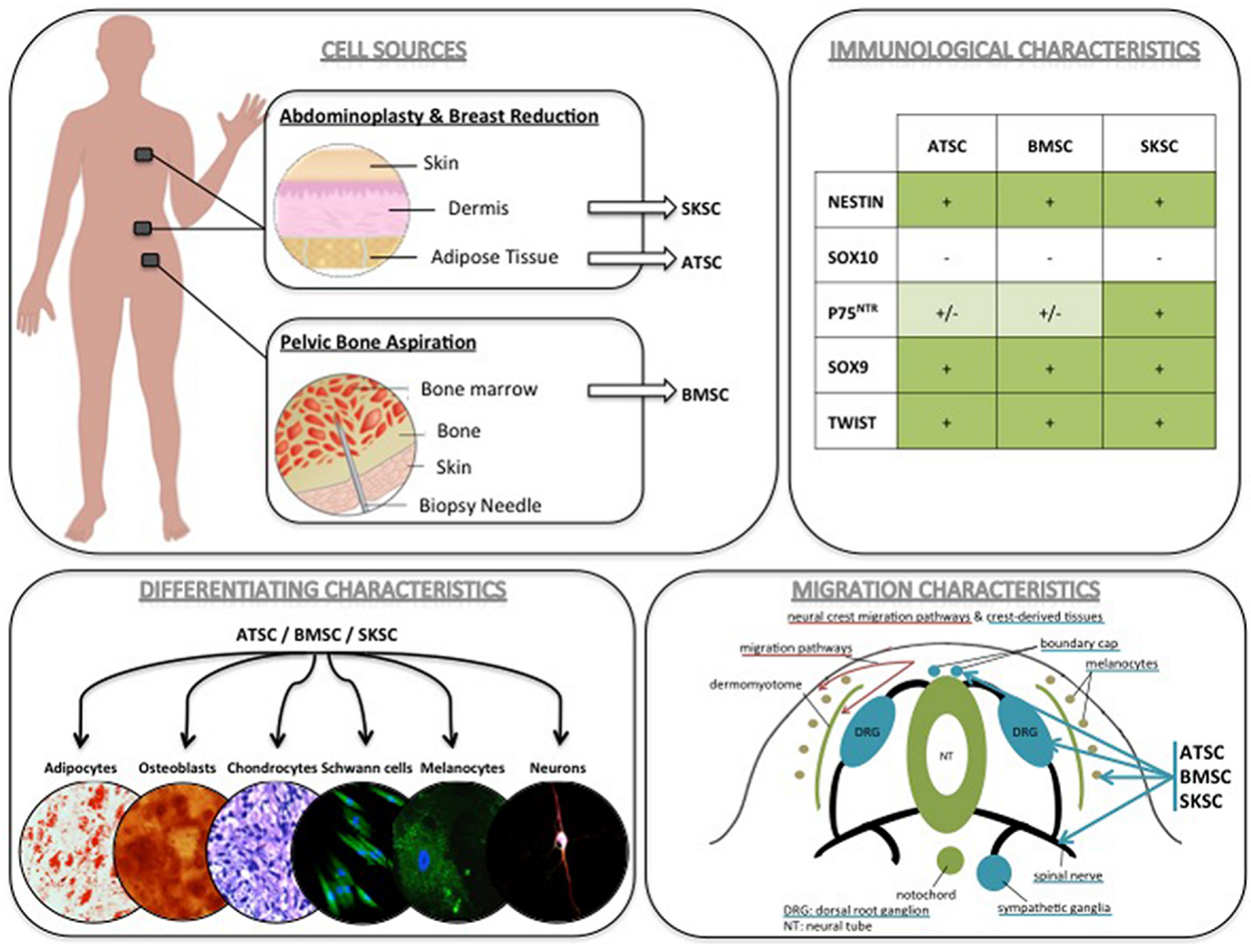
Summary: NCSC identification in adult human bone marrow, adipose tissue and dermis. The phenotypic characterization, multipotency and the migration abilities of ATSC, BMSC and SKSC strongly suggest the presence of neural crest-derived stem cells in human adult adipose tissue and bone marrow.

Altogether, and as summarized in Fig 13, the phenotypic characterization, the differentiating and the migrating abilities of ATSC, BMSC and SKSC strongly suggest the presence of neural crest-derived cells in human bone marrow, as it has already been described for skin and adipose tissue.

## Supporting information

S1 Fig Characterization of migration abilities of adherent bone marrow, adipose tissue and dermis-derived stem cells when injected in chick embryos: Localization of migrating cells. Fig 10 represents transversal (a-o) and longitudinal (p) sections of adherent cells injected into HHSt18 chick embryos. Human stem cells derived from adipose tissue, bone marrow and dermis were localized into chick DRG (a-c), boundary cap of the NT (d-f), injection site (g-i), skin or more precisely melanocyte region (j-l) and finally the fiber track leaving the DRG (m-o). Fig 10p presents longitudinal section with magnification on migrating cells along the neural tube. (Scale bars = 50μm, Green: TUJ1 labeling, Red: human nuclei labeling, Blue: DAPI labeling).
(TIF)

S2 Fig Characterization of migration abilities of bone marrow, adipose tissue and dermis-derived spheres when injected in chick embryos: Localization of migrating cells. Fig 11 represents transversal sections of spheres injected into HHSt18 chick embryos. Human stem cells derived from adipose tissue, bone marrow and dermis were localized into chick DRG (a-c), boundary cap of the NT (d-f), injection site (g-i), skin or more precisely melanocyte region (j-l) and finally the fiber track leaving the DRG (m-o). (Scale bars = 50μm, Green: TUJ1 labeling, Red: human nuclei labeling, Blue: DAPI labeling).
(TIF)

S3 Fig Comparision of migration abilities of bone marrow-derived spheres compared to iPS-derived NSC when injected in chick embryos: Localization of migrating cells. **A to H.** As observed on figure A and E, NSC migrated in the DRG (**D and H**) like BMSC, however, those cells also colonized different part of the embryos as the neural tube (**C and G**) where no BMSC cells were ever observed **(J and N)**. (Scale bars = 50μm, Green: TUJ1 labeling, Red: human nuclei labeling, Blue: DAPI). White arrowheads indicate the position of human NSC (Red: human nuclei) whithin the chick embryo.
(EPS)
