# Correction: Human bone marrow harbors cells with neural crest-associated characteristics like human adipose and dermis tissues

**DOI:** 10.1371/journal.pone.0256484

**Published:** 2021-09-28

**Authors:** Cécile Coste, Virginie Neirinckx, Anil Sharma, Gulistan Agirman, Bernard Rogister, Jacques Foguenne, François Lallemend, André Gothot, Sabine Wislet

Following the publication of this article [1] concerns were raised regarding results presented in Figs 1 and 6, S1 and S2 Figs. Specifically,

- The SOX10 panel in Fig 1F appears similar to the SOX10 results presented in Fig 2C of [2], the SOX10 results presented in Figure 1D of [3], and the SOX10 results presented in Fig 1E of [4].
- The Fig 6 panels I, J, and K partially overlap with one another, despite being used to represent different experimental conditions.
- The Boundary Cap BMSC panel of S1 Fig appears similar to the Boundary Cap BMSC panel of S2 Fig, despite being used to represent different experimental conditions.

The authors explained that this study follows up from the results previously described in their *Cellular and Molecular Life Sciences* and *PLOS ONE* articles [2, 3, 4], and that the SOX10 panel in Fig 1F represents the same experimental conditions as the SOX10 panels presented in these earlier publications.

**Fig 1:**
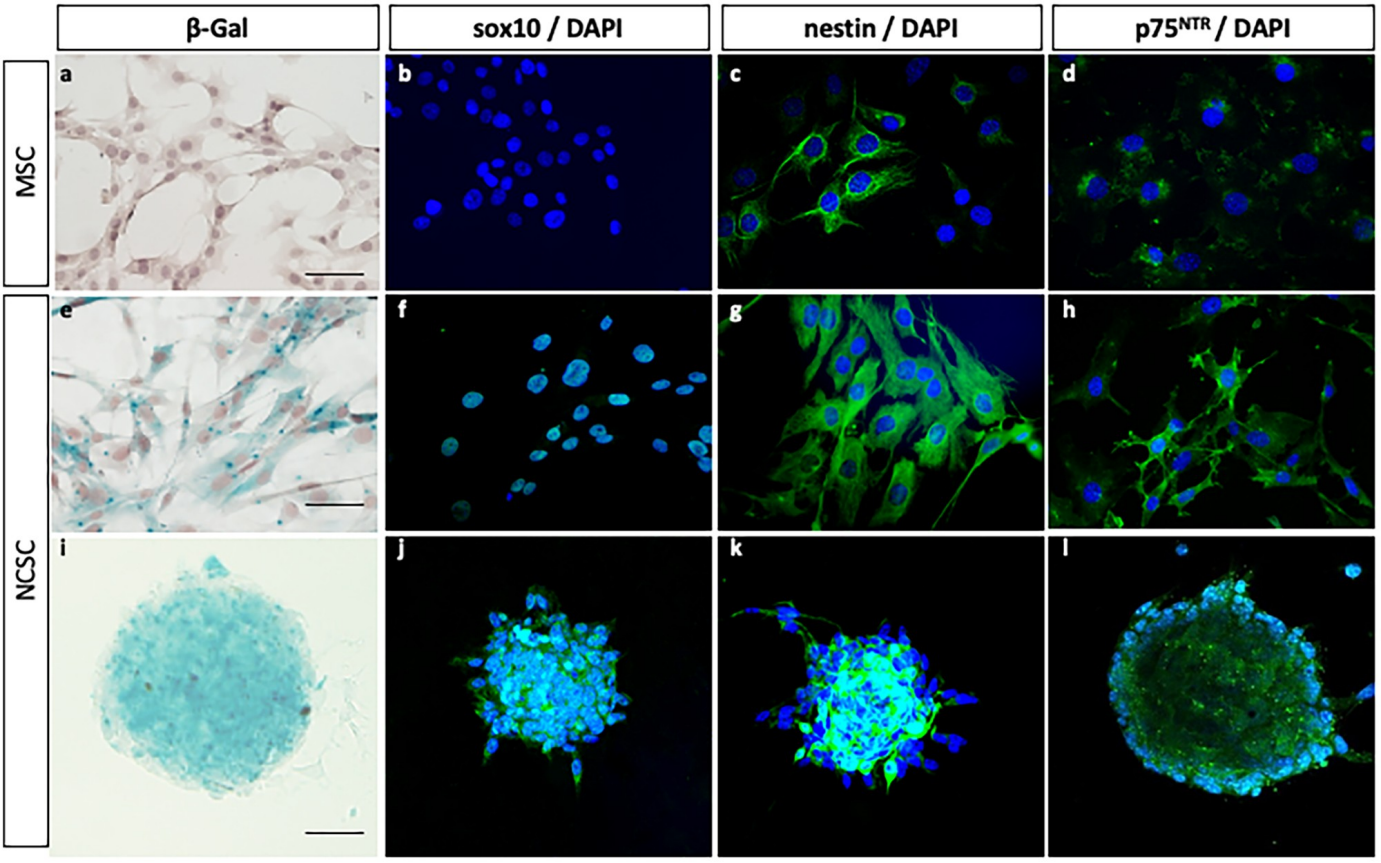
Characteristics of mouse NCSC and MSC from adult bone marrow. NCSC obtained from Wnt1-Cre/R26R-LacZ mice expressing *LacZ*. In adherent culture conditions, MSC did not express β-galactosidase (a) neither sox10 (b), but a small and constant proportion of cells slightly expressed nestin (c) and p75^NTR^ (d). NCSC expressed β-galactosidase (e), sox10 (f), Nestin (g) and p75^NTR^ (h). Finally, among these two populations, only NCSC were able to grow as spheres; spheres also express β-galactosidase (i), Sox10 (j), Nestin (k) and p75^NTR^ (l). (Scale bars = 40μm).

Furthermore, the authors explained that the Fig 6I, 6J and 6K panels were wrongly collected from the same human bone marrow sample, as opposed to the cell types mentioned in the figure legend. The authors clarify that the wrong images were inadvertently used during figure preparation and explain that the correct samples were used for the quantification and the preparation of the associated graphs. In the updated figure below, the authors provided the correct panels for the chondrocytic differentiation of bone marrow (panel I), adipose tissue (panel J) and dermis-derived stem cells (panel K).

**Fig 6:**
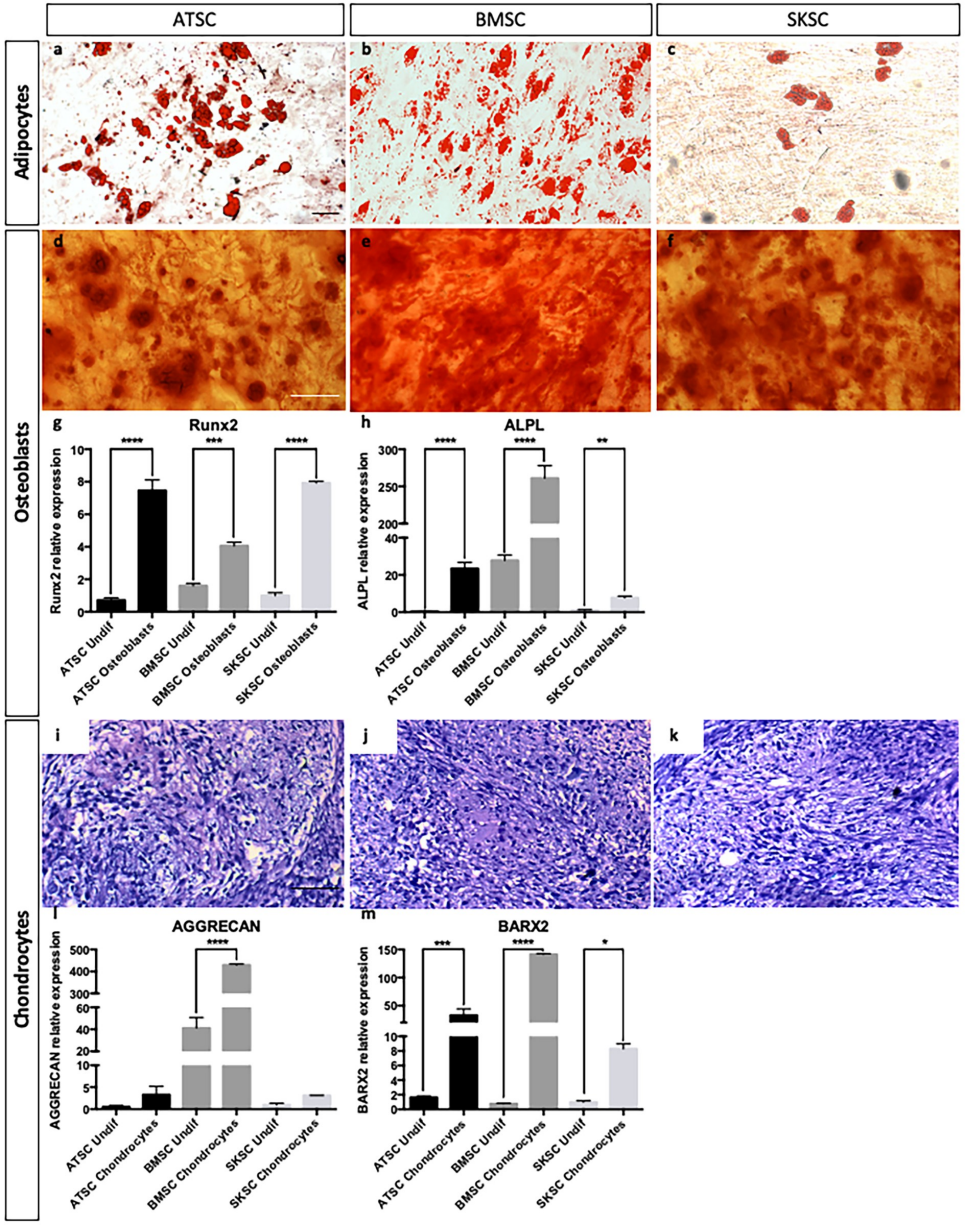
Differentiation abilities of human bone marrow, adipose tissue and dermis-derived stem cells. ATSC, BMSC and SKSC were able to differentiate into adipocytes (lipids vacuoles stained with Oil Red-O—a-c), osteoblast (calcium deposits stained with Alizarin Red—d-f), chondrocytes (chondrocyte matrix stained with Alcian Blue—i-k). Osteoblastic and chondrogenic differentiations were also assessed by quantitative RT-PCR based on *RUNX2* (g), *ALPL* (h), *AGGRECAN* (j) and *BARX2* (k) expression levels. Data were normalized to SKSC undifferentiated cells expression level set as 1. Statistical analysis: one way ANOVA followed by HSD post hoc test. * means p<0.05, *** means p<0.0005, **** means p<0.0001. (Scale bars = 50μm).

Finally, the authors explained that the Boundary Cap BMSC panels were inadvertently duplicated within S1 and S2 Figs. In addition, the authors pointed out that S1 and S2 Figs were inverted in the original version of the article. The figure originally listed as S1 Fig is updated as S2 Fig. The figure originally listed as S2 Fig has been corrected to replace the duplicate Boundary Cap BMSC panel and is listed as S1 Fig in this correction notice. The originally published, uncorrected version of S2 Fig, updated to S1 Fig in this correction notice, is provided in the S7 File below.

The Board of Ethics and Scientific Integrity of University of Liège investigated the overlap between the aforementioned panels and recommended the article be corrected. In addition, a member of *PLOS ONE’s* Editorial Board advised that the updated figures support the results and conclusions reported in the original article. As the original Fig 2C presented in [2] is not licensed for reproduction and distribution under the terms of the Creative Commons Attribution License (or Public Domain License for US gov), this article was republished on September 28, 2021 to remove Fig 1F and replace it with an alternative relevant immunological characterization image. Please download this article again to view the correct version.

## Supporting information

S1 Fig Characterization of adherent bone marrow, adipose tissue and dermis-derived stem cells migration ability when injected in chick embryos: Localization of migrating cells. Similarly to Fig 10, this figure represents transversal (a-o) and longitudinal (p) sections of adherent cells injected into HHSt18 chick embryos. Human stem cells derived from adipose tissue, bone marrow and dermis are localized into chick DRG (a-c), boundary cap of the NT (d-f), injection site (g-i), skin or more precisely melanocyte region (j-l) and finally the fiber track leaving the DRG (m-o). Fig. 10p presents longitudinal section with magnification on migrating cells along the neural tube. (Scale bars = 50μm, Green: TUJ1 labeling, Red: human nuclei labeling, Blue: DAPI labeling).
(TIF)

S2 Fig Characterization of spheres from bone marrow, adipose tissue and dermis-derived stem cells migration ability when injected in chick embryos: Localization of migrating cells. Similarly to Fig 11, this figure represents transversal sections of spheres injected into HHSt18 chick embryos. Human stem cells derived from adipose tissue, bone marrow and dermis are localized into chick DRG (a-c), injection site (d-f), boundary cap of the NT (g-i), skin or more precisely melanocyte region (j-l) and finally the fiber track leaving the DRG (m-o). (Scale bars = 50μm, Green: TUJ1 labeling, Red: human nuclei labeling, Blue: DAPI labeling).
(TIF)

S1 File Raw data underlying Fig 1. (ZIP)

S2 File Raw data underlying Fig 6A–6F and 6I–6K. (ZIP)

S3 File Raw data underlying Fig 6G, 6H, 6L and 6M. (ZIP)

S4 File Raw data underlying S1 Fig. (ZIP)

S5 File Raw data underlying S2 Fig. (ZIP)

S6 File Originally published, uncorrected article with copyrighted image removed. (PDF)

S7 File Originally published, uncorrected version of S2 Fig, updated to S1 Fig in this notice. (EPS)
