# Supplementary figures and images for: Human bone marrow harbors cells with neural crest-associated characteristics like human adipose and dermis tissues
Source: PLoS One. 2017 Jul 6;12(7):e0177962. doi: 10.1371/journal.pone.0177962 (PMC5500284; doi:10.1371/journal.pone.0177962)

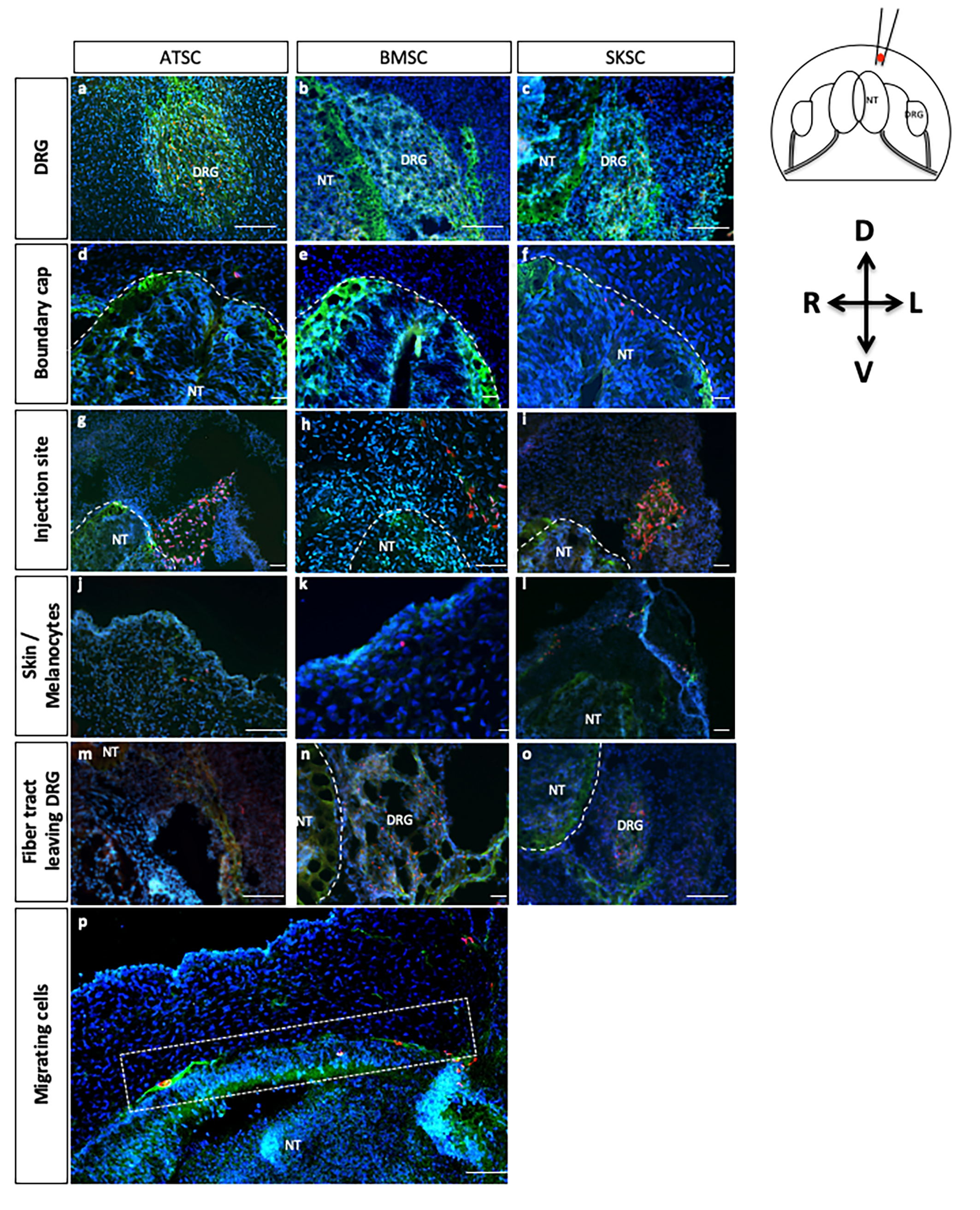

Supplement: S1 Fig — Fig 10 represents transversal (a-o) and longitudinal (p) sections of adherent cells injected into HHSt18 chick embryos. Human stem cells derived from adipose tissue, bone marrow and dermis were localized into chick DRG (a-c), boundary cap of the NT (d-f), injection site (g-i), skin or more precisely melanocyte region (j-l) and finally the fiber track leaving the DRG (m-o). Fig 10p presents longitudinal section with magnification on migrating cells along the neural tube. (Scale bars = 50μm, Green: TUJ1 labeling, Red: human nuclei labeling, Blue: DAPI labeling). (TIF) [file pone.0177962.s001.tif]

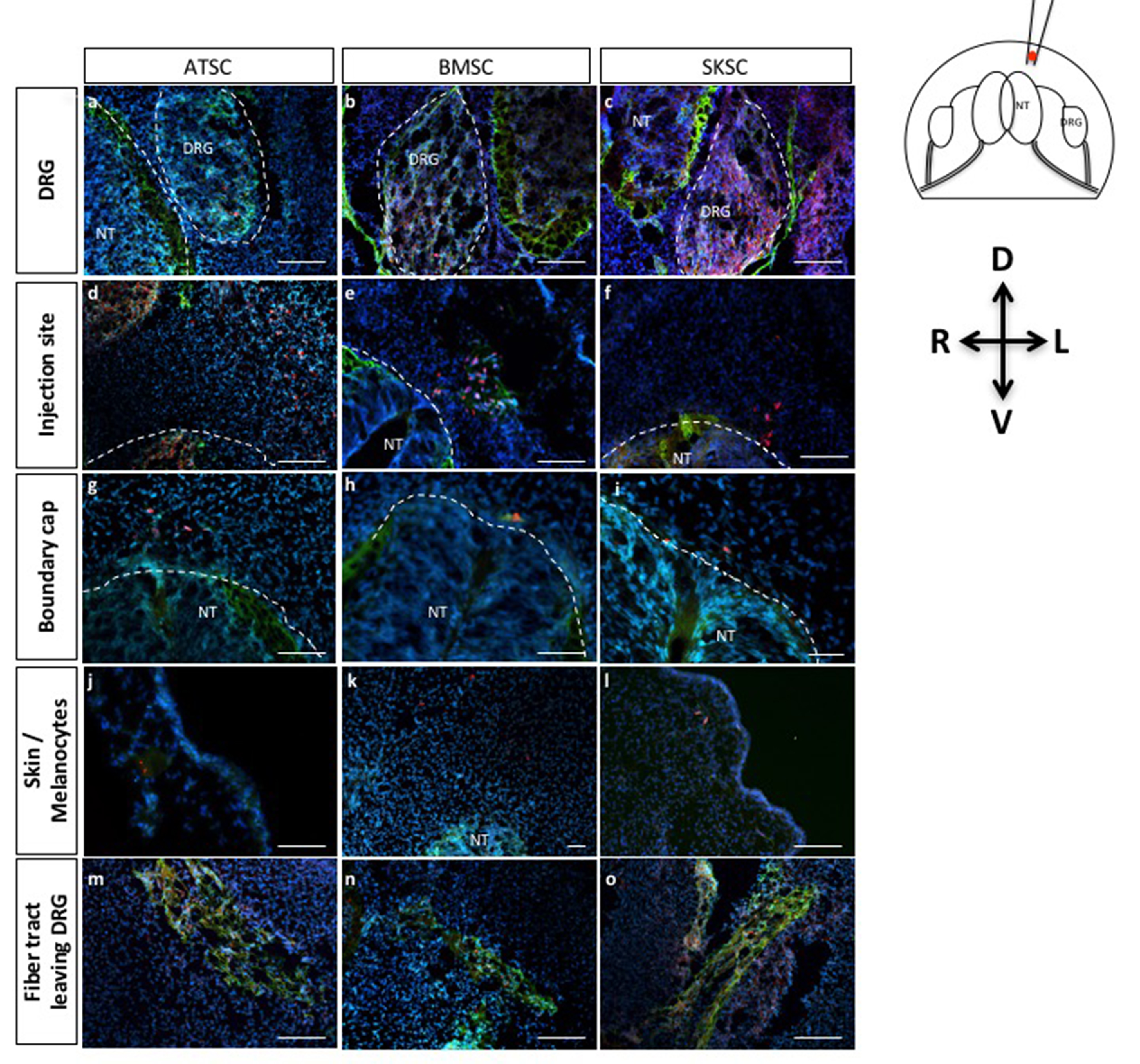

Supplement: S2 Fig — Fig 11 represents transversal sections of spheres injected into HHSt18 chick embryos. Human stem cells derived from adipose tissue, bone marrow and dermis were localized into chick DRG (a-c), boundary cap of the NT (d-f), injection site (g-i), skin or more precisely melanocyte region (j-l) and finally the fiber track leaving the DRG (m-o). (Scale bars = 50μm, Green: TUJ1 labeling, Red: human nuclei labeling, Blue: DAPI labeling). (TIF) [file pone.0177962.s002.tif]
