# Supplementary figures and images for: Correction: Human bone marrow harbors cells with neural crest-associated characteristics like human adipose and dermis tissues
Source: PLoS One. 2021 Sep 28;16(9):e0256484. doi: 10.1371/journal.pone.0256484 (PMC8478174; doi:10.1371/journal.pone.0256484)

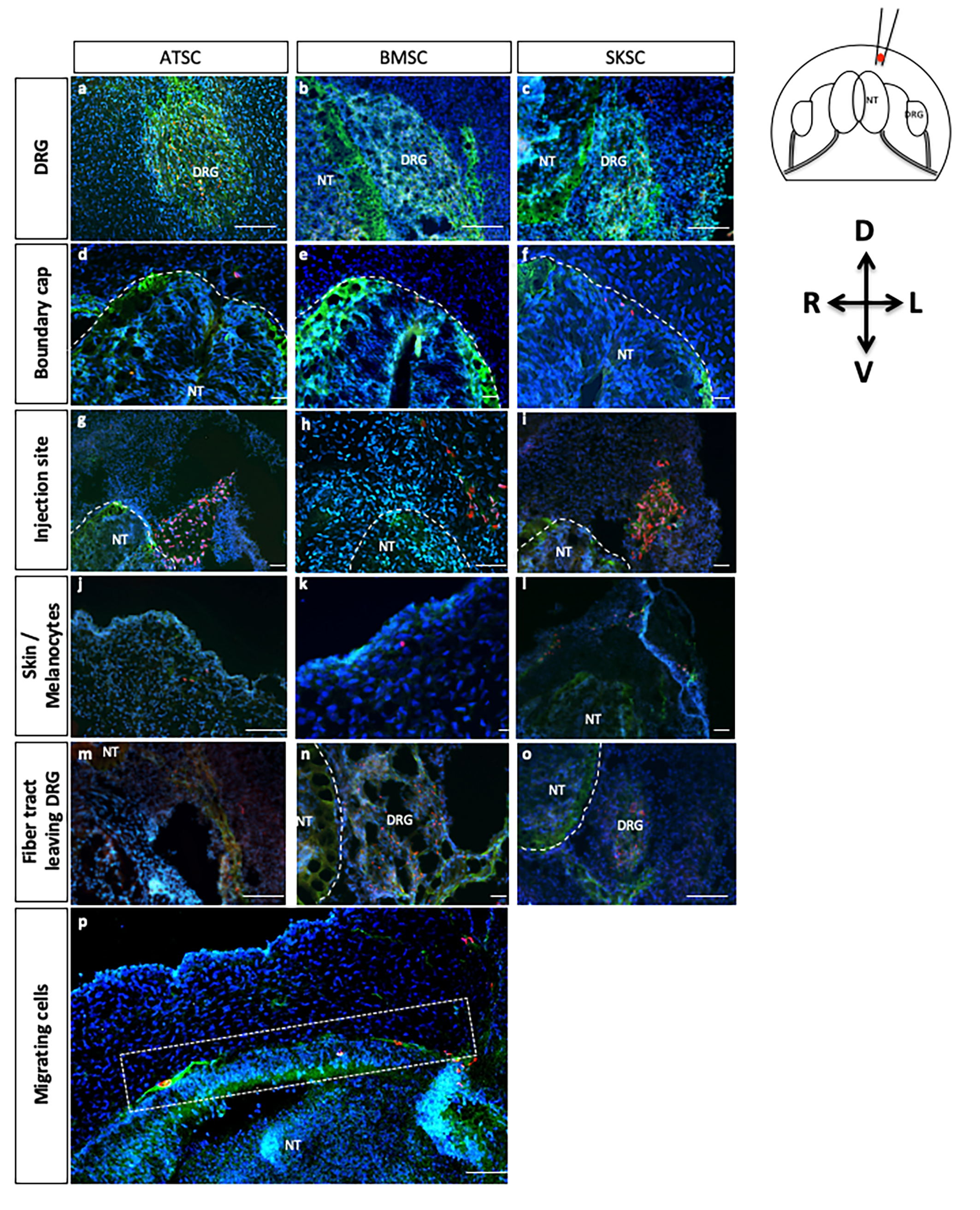

Supplement: S1 Fig — Similarly to Fig 10, this figure represents transversal (a-o) and longitudinal (p) sections of adherent cells injected into HHSt18 chick embryos. Human stem cells derived from adipose tissue, bone marrow and dermis are localized into chick DRG (a-c), boundary cap of the NT (d-f), injection site (g-i), skin or more precisely melanocyte region (j-l) and finally the fiber track leaving the DRG (m-o). Fig. 10p presents longitudinal section with magnification on migrating cells along the neural tube. (Scale bars = 50μm, Green: TUJ1 labeling, Red: human nuclei labeling, Blue: DAPI labeling). (TIF) [file pone.0256484.s001.tif]

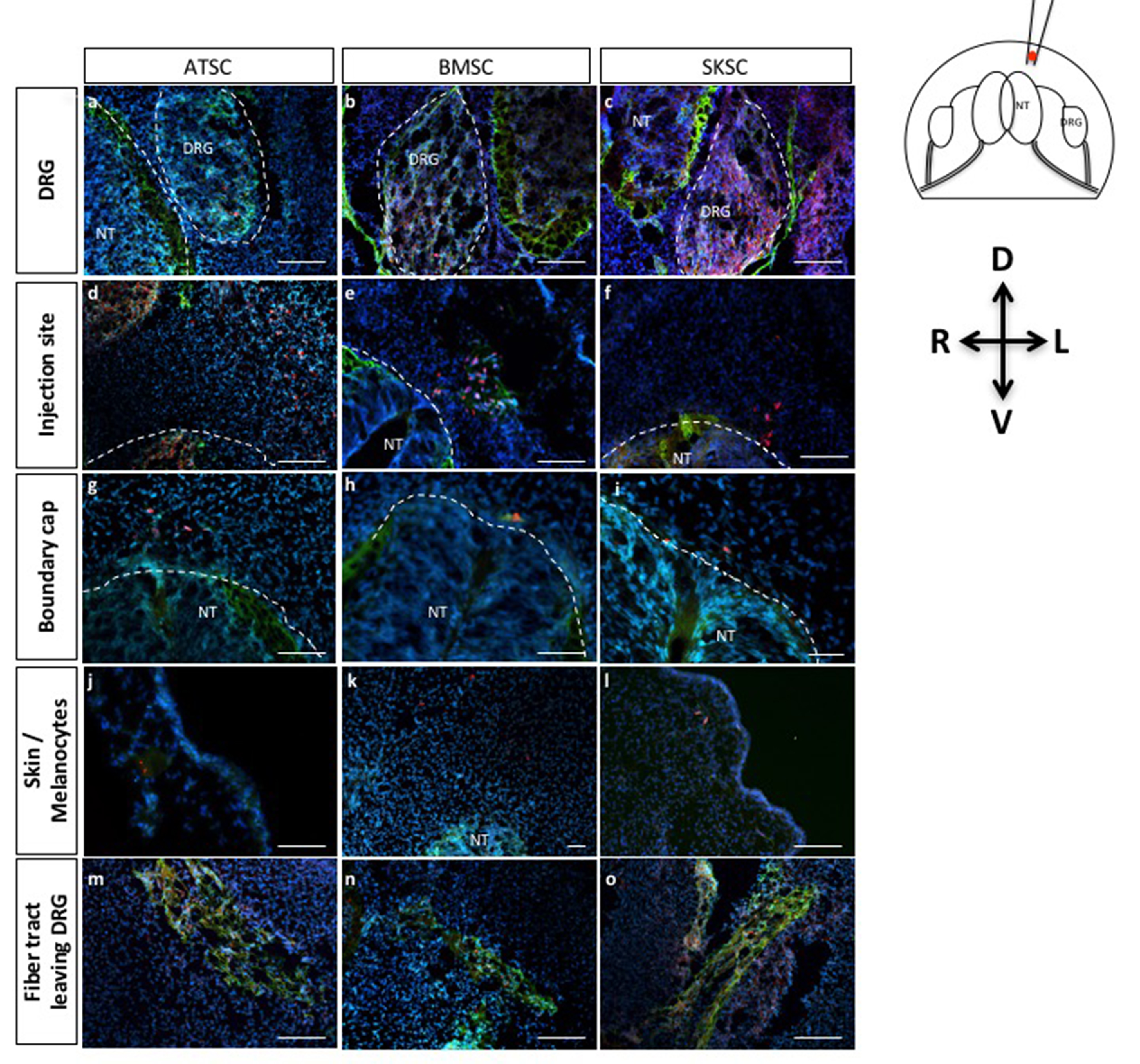

Supplement: S2 Fig — Similarly to Fig 11, this figure represents transversal sections of spheres injected into HHSt18 chick embryos. Human stem cells derived from adipose tissue, bone marrow and dermis are localized into chick DRG (a-c), injection site (d-f), boundary cap of the NT (g-i), skin or more precisely melanocyte region (j-l) and finally the fiber track leaving the DRG (m-o). (Scale bars = 50μm, Green: TUJ1 labeling, Red: human nuclei labeling, Blue: DAPI labeling). (TIF) [file pone.0256484.s002.tif]

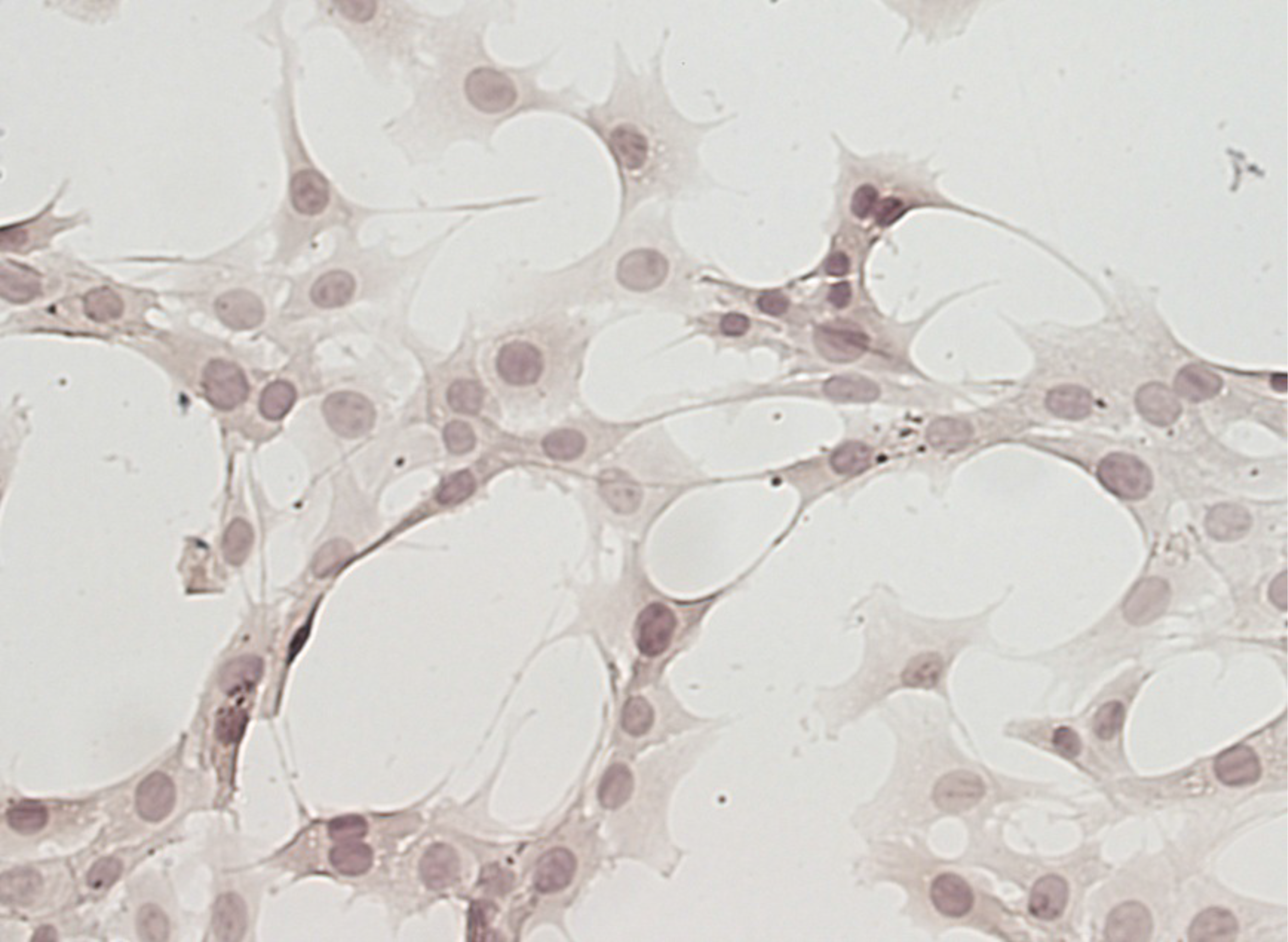

Supplement: S1 File — (ZIP) [file pone.0256484.s003.zip › S1 File/Figure 1A.tif]

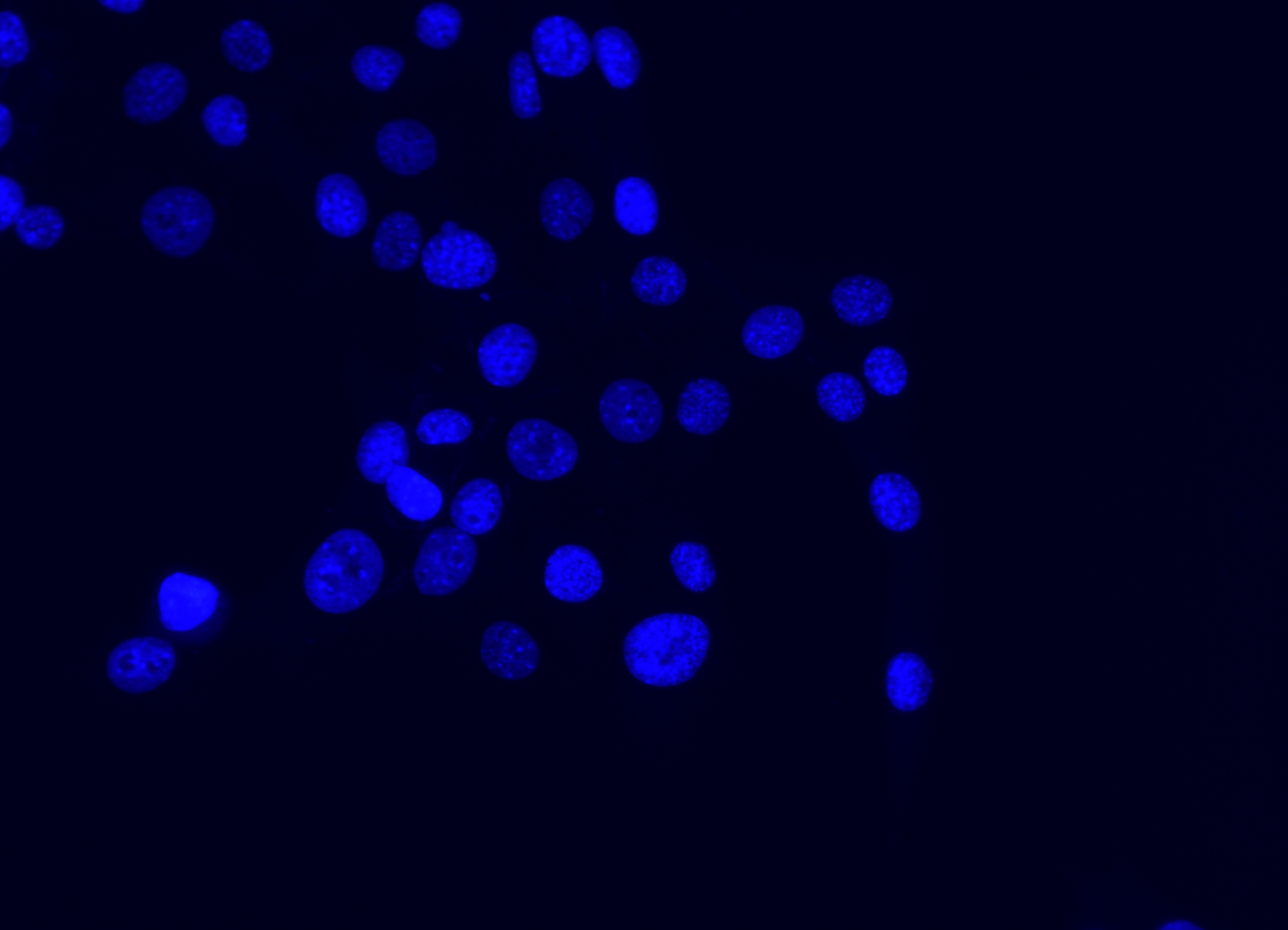

Supplement: S1 File — (ZIP) [file pone.0256484.s003.zip › S1 File/Figure 1B.tif]

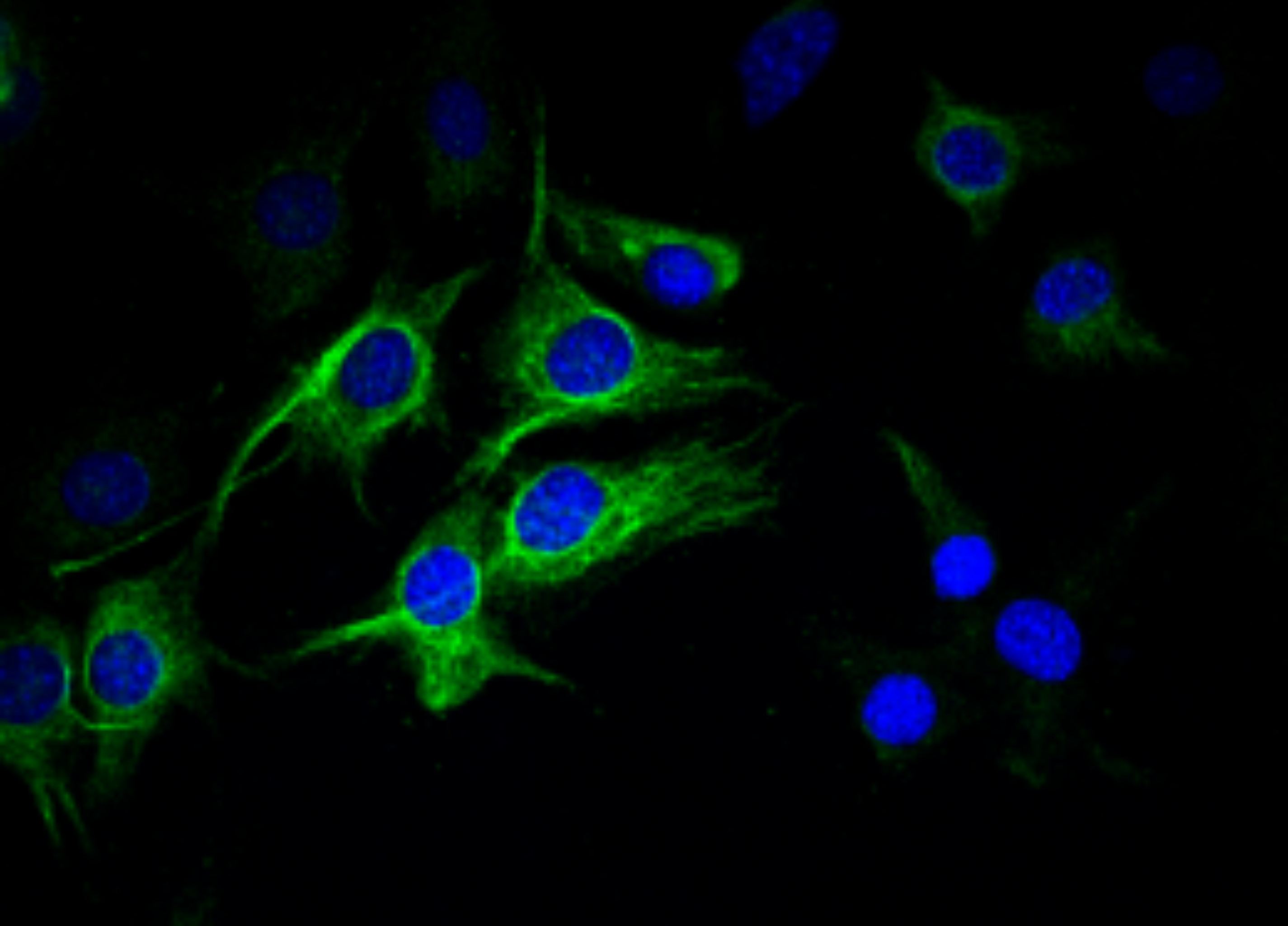

Supplement: S1 File — (ZIP) [file pone.0256484.s003.zip › S1 File/Figure 1C.tif]

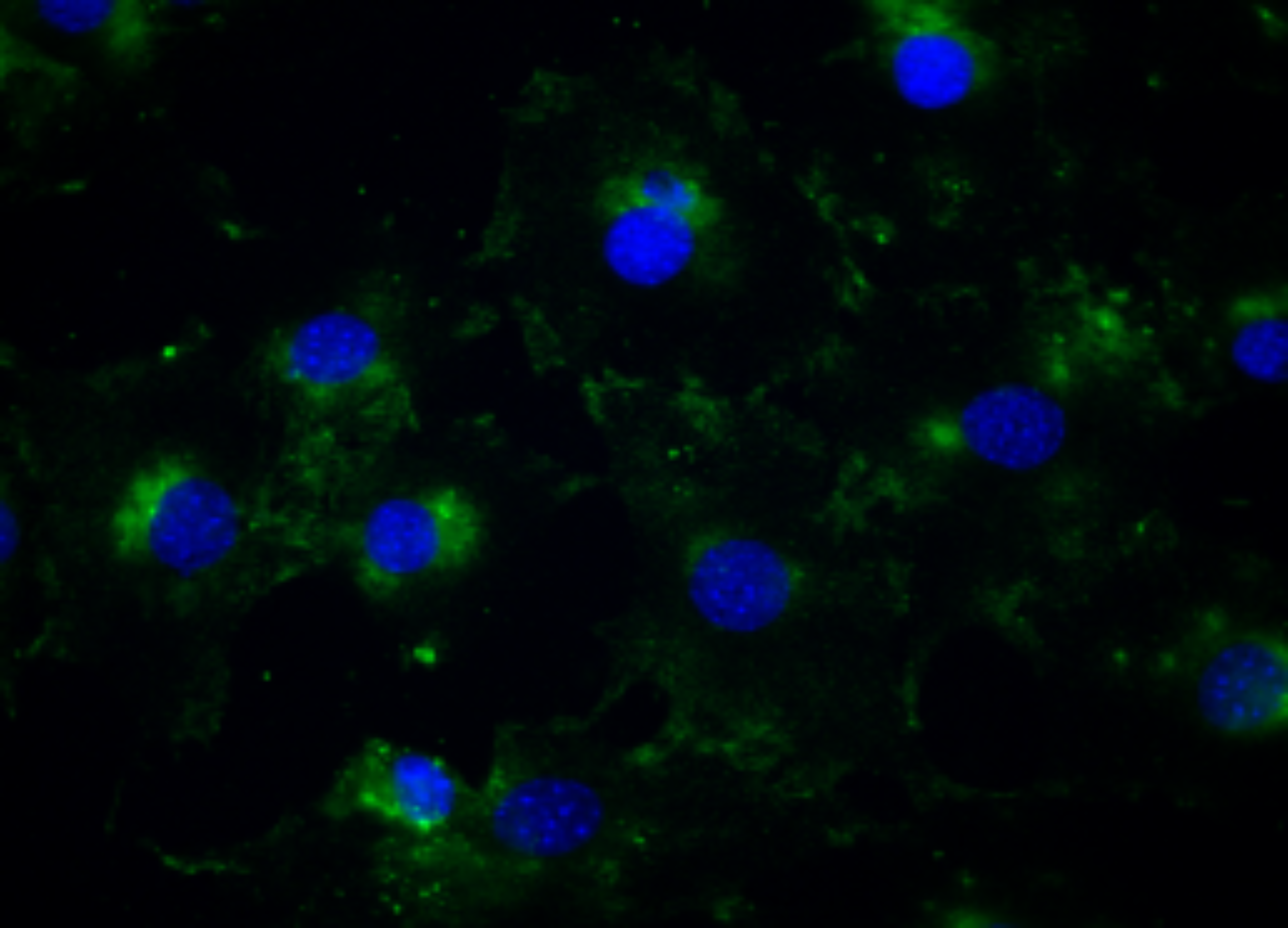

Supplement: S1 File — (ZIP) [file pone.0256484.s003.zip › S1 File/Figure 1D.tif]

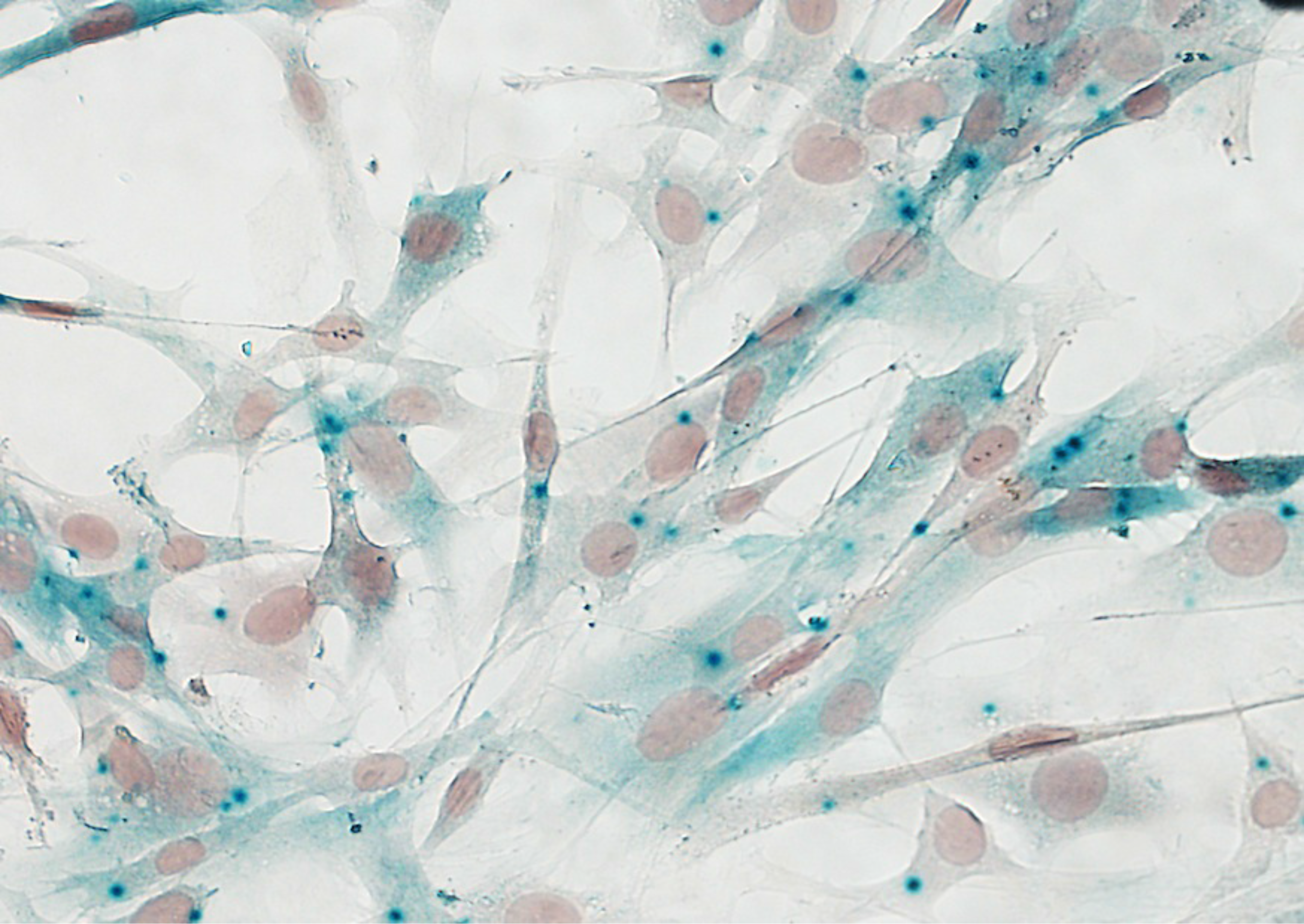

Supplement: S1 File — (ZIP) [file pone.0256484.s003.zip › S1 File/Figure 1E.tif]

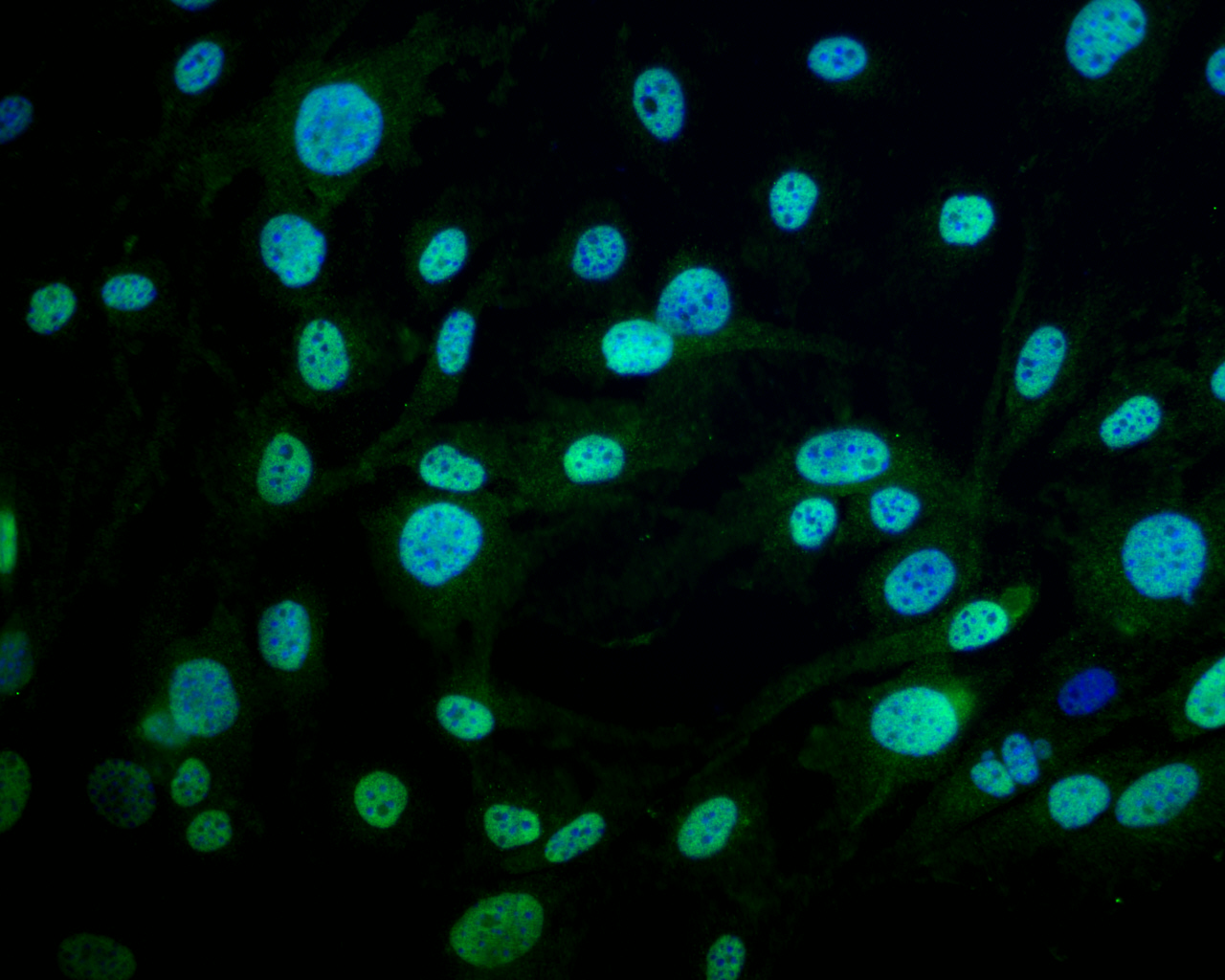

Supplement: S1 File — (ZIP) [file pone.0256484.s003.zip › S1 File/Figure 1F.tif]

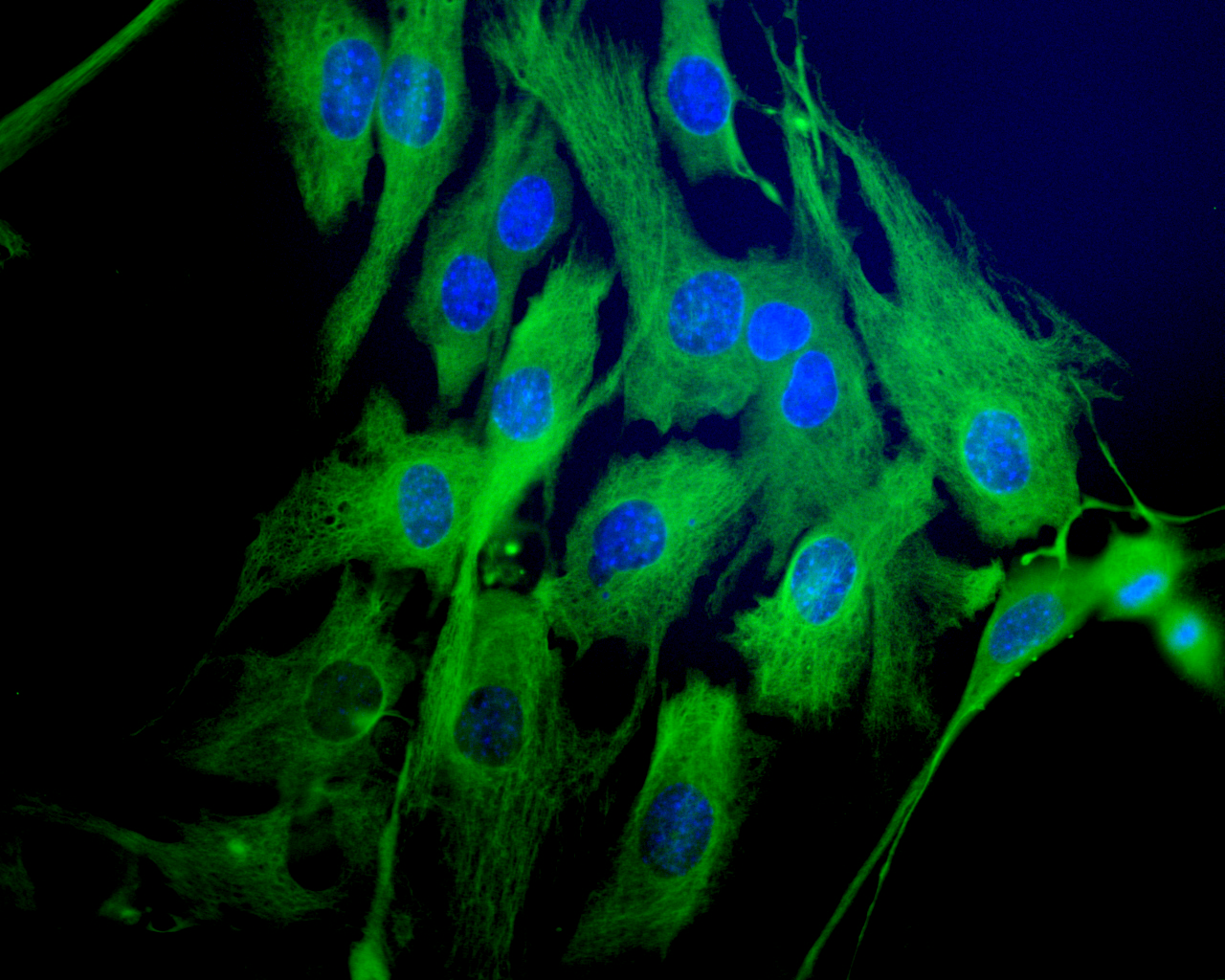

Supplement: S1 File — (ZIP) [file pone.0256484.s003.zip › S1 File/Figure 1G.tif]

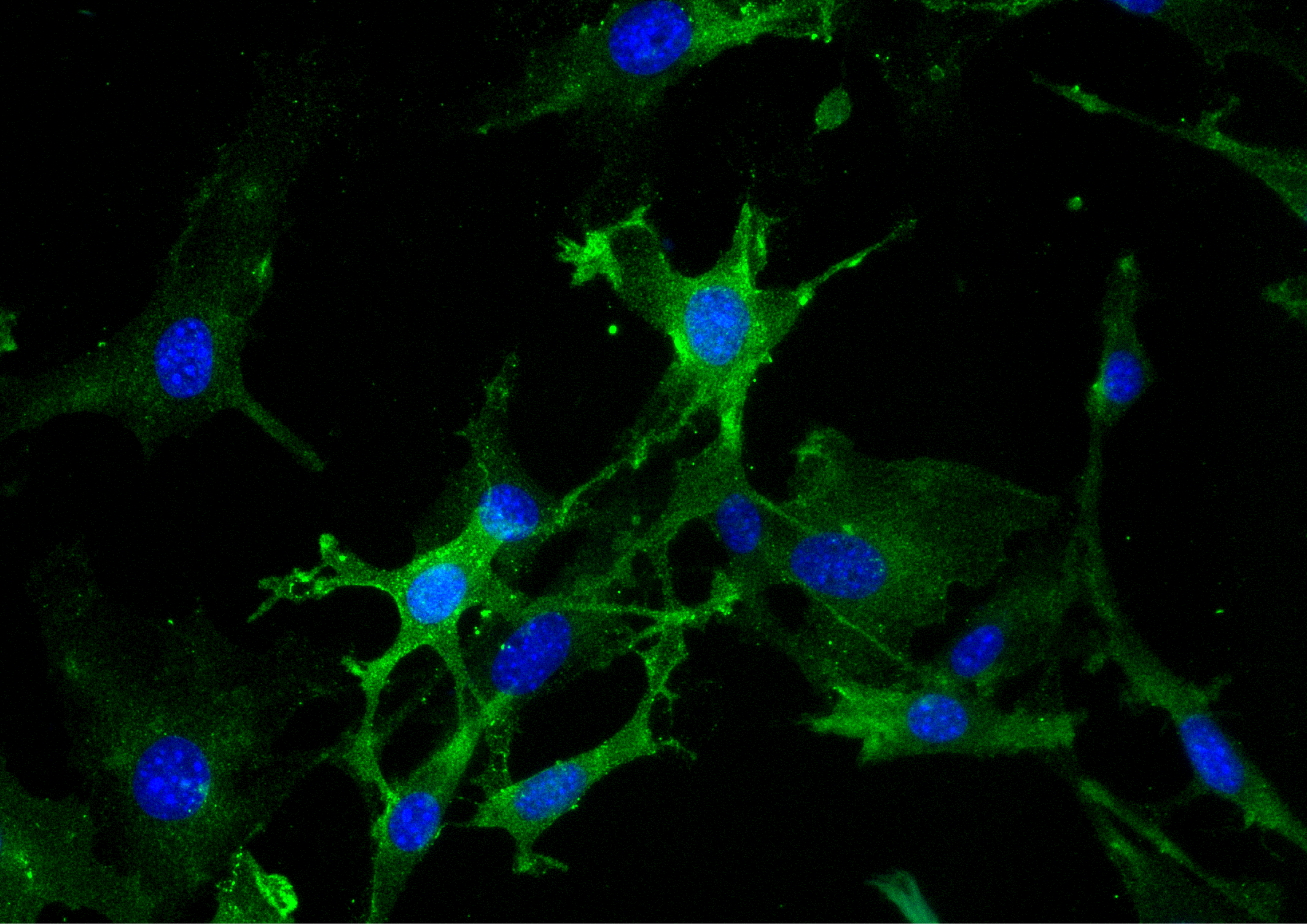

Supplement: S1 File — (ZIP) [file pone.0256484.s003.zip › S1 File/Figure 1H.tif]

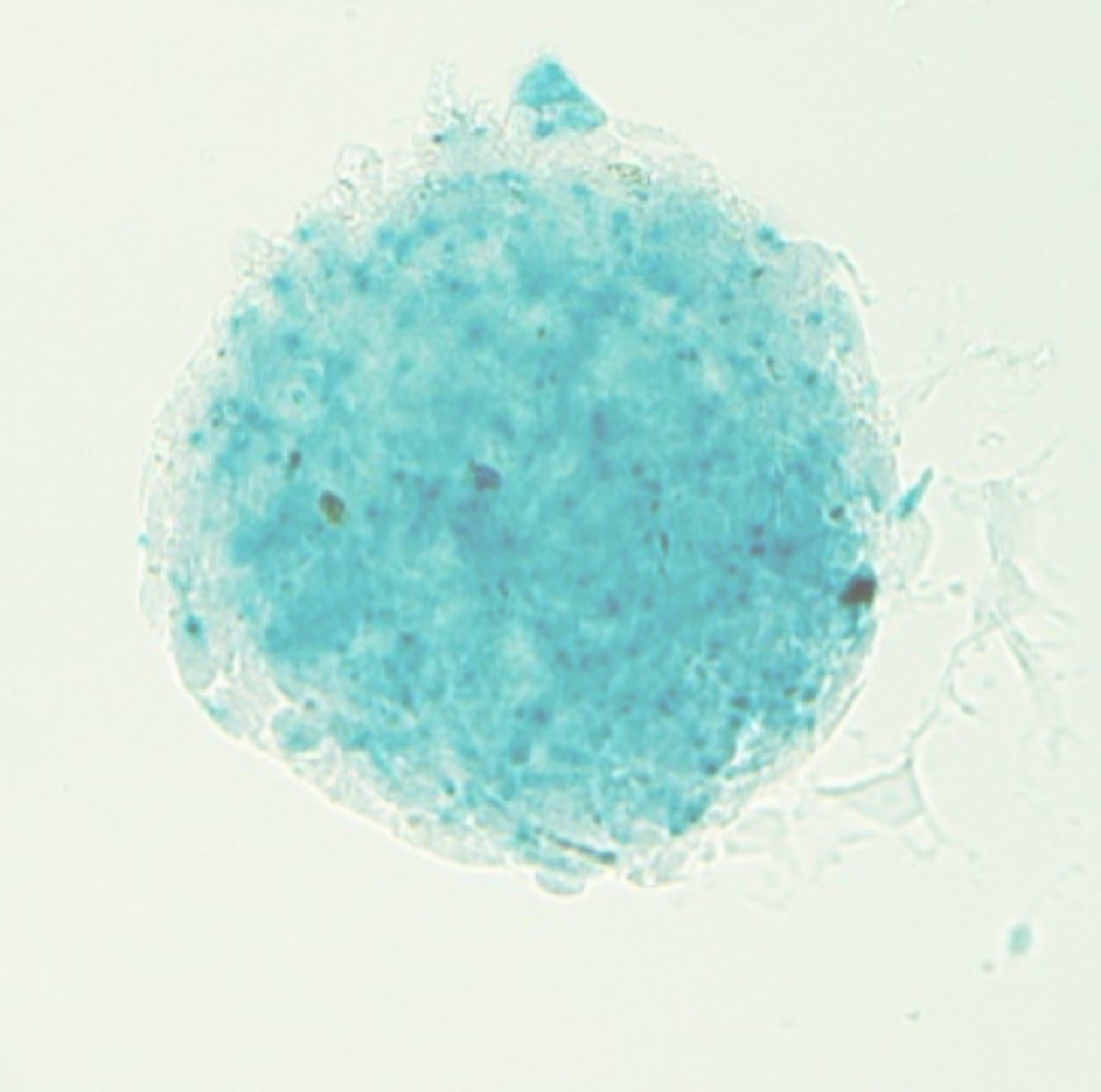

Supplement: S1 File — (ZIP) [file pone.0256484.s003.zip › S1 File/Figure 1I.tif]

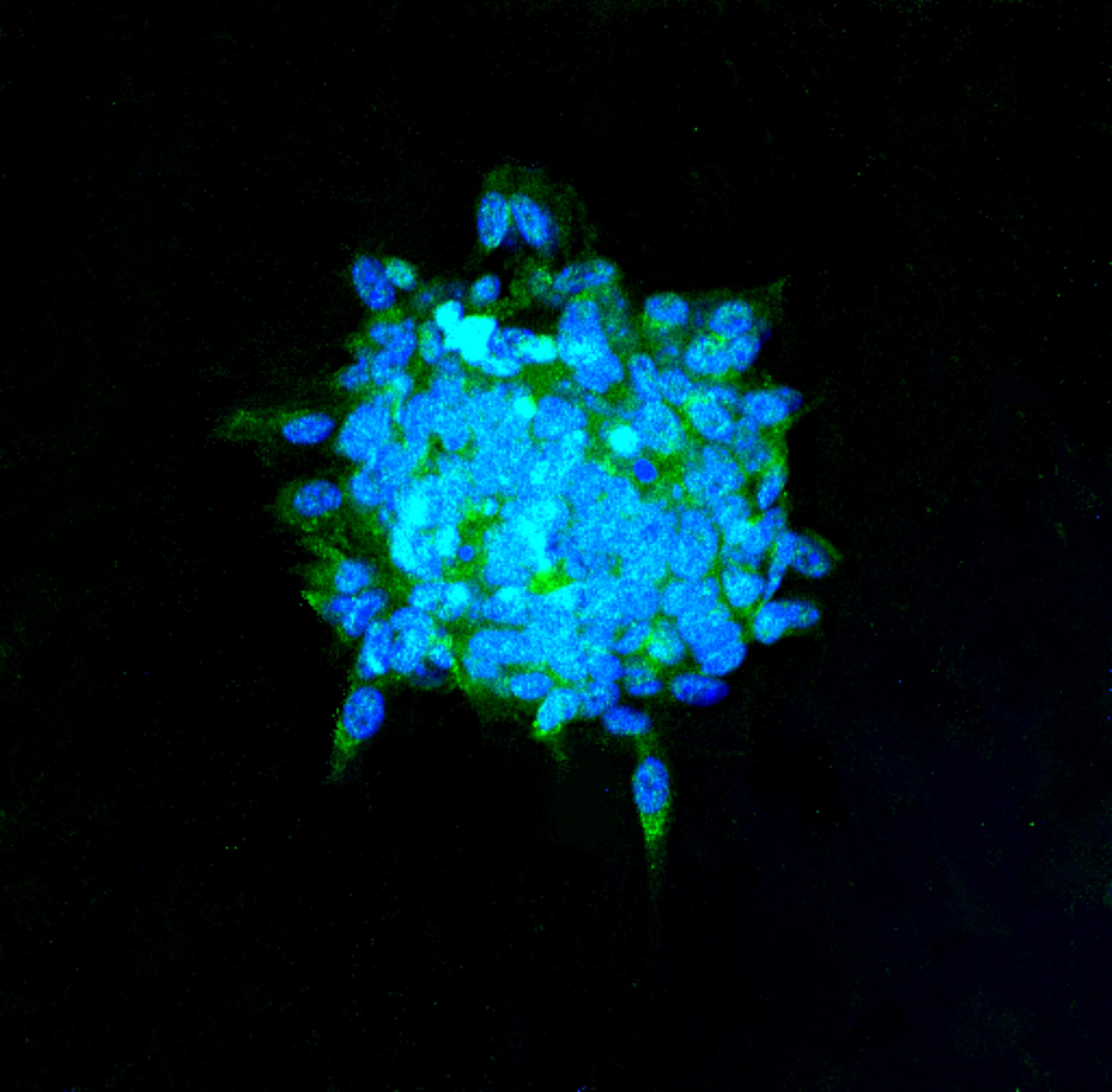

Supplement: S1 File — (ZIP) [file pone.0256484.s003.zip › S1 File/Figure 1J.tif]

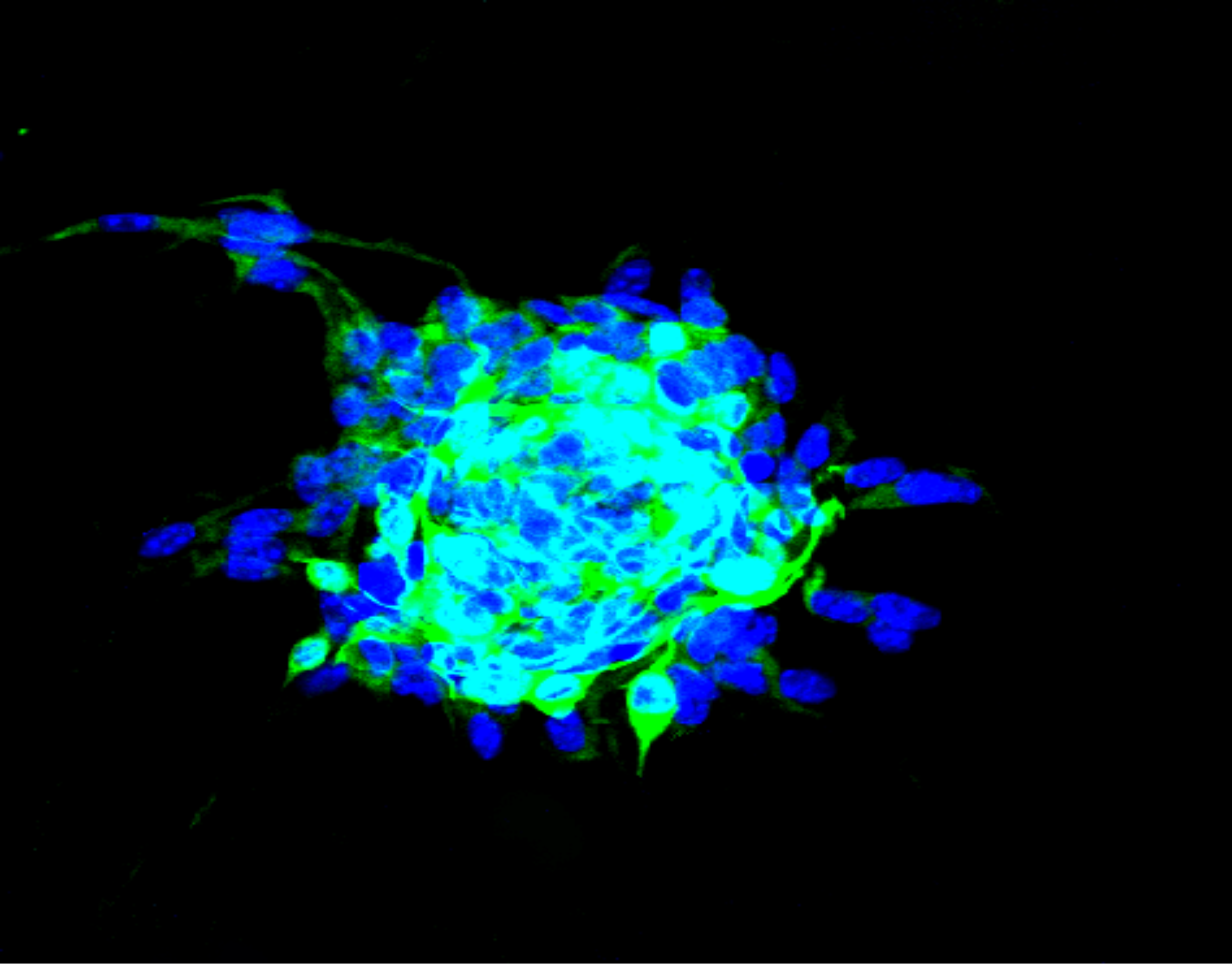

Supplement: S1 File — (ZIP) [file pone.0256484.s003.zip › S1 File/Figure 1K.tif]

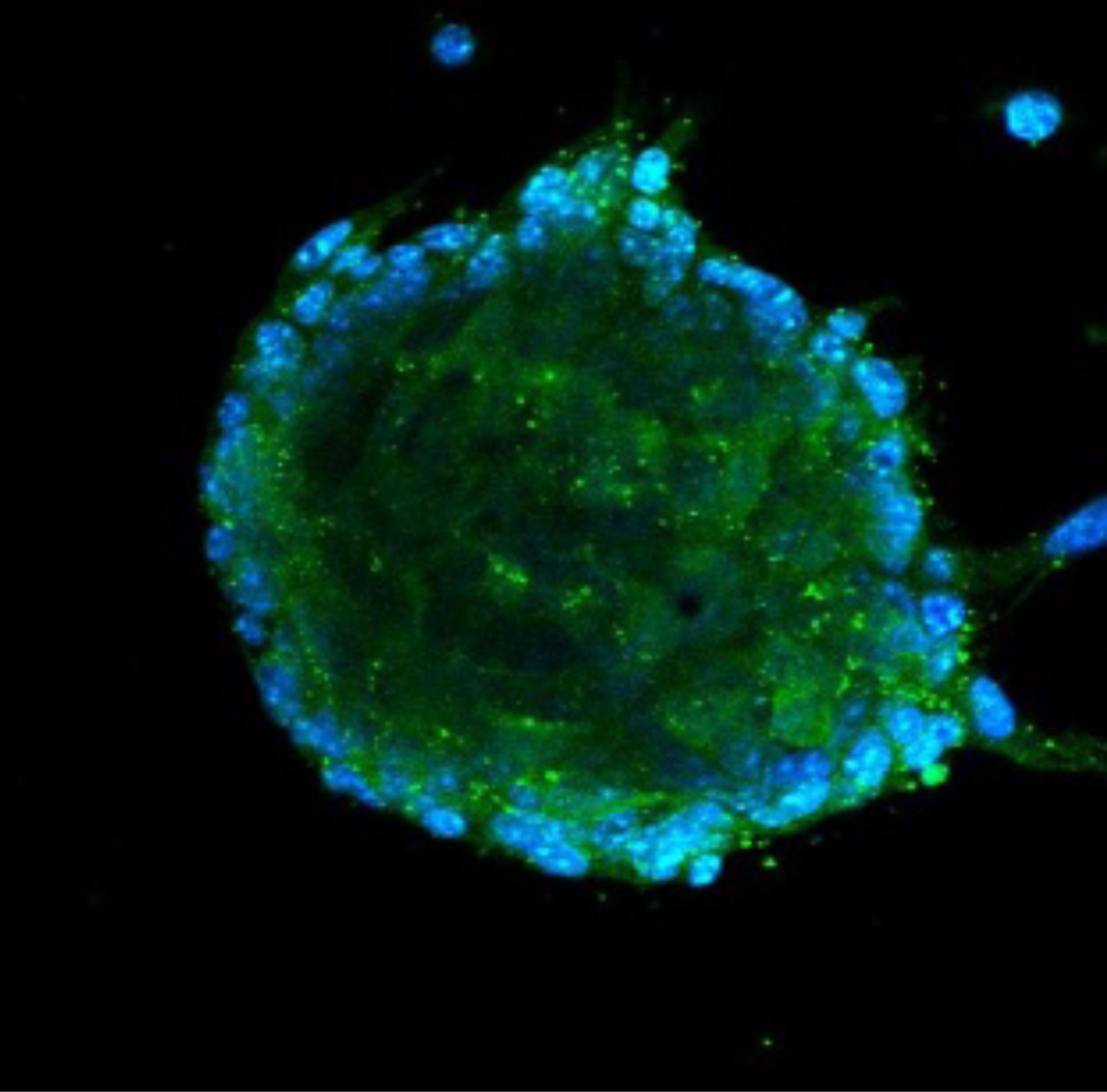

Supplement: S1 File — (ZIP) [file pone.0256484.s003.zip › S1 File/Figure 1L.tif]

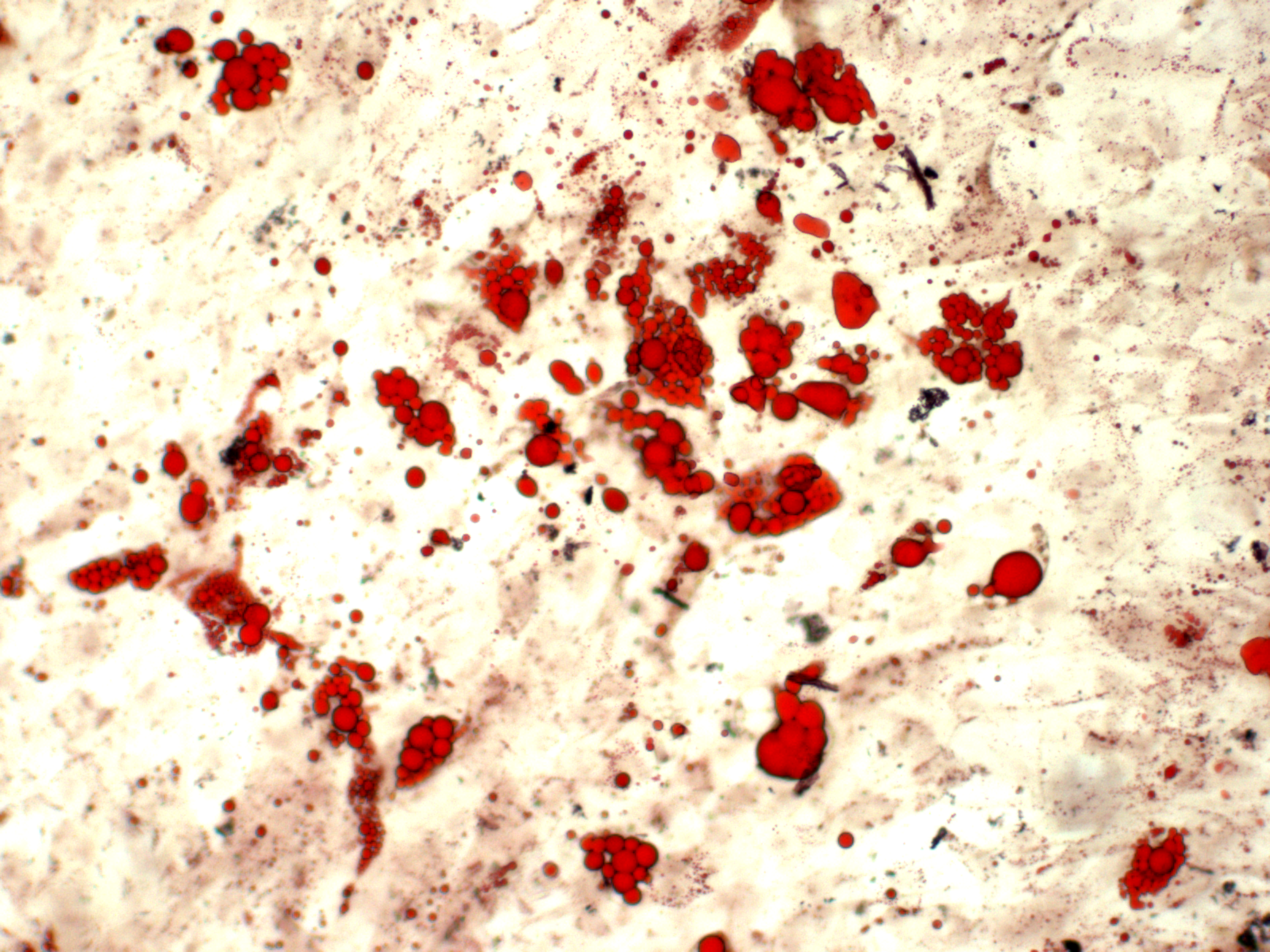

Supplement: S2 File — (ZIP) [file pone.0256484.s004.zip › S2 File/Figure 6A C COSTE.tif]

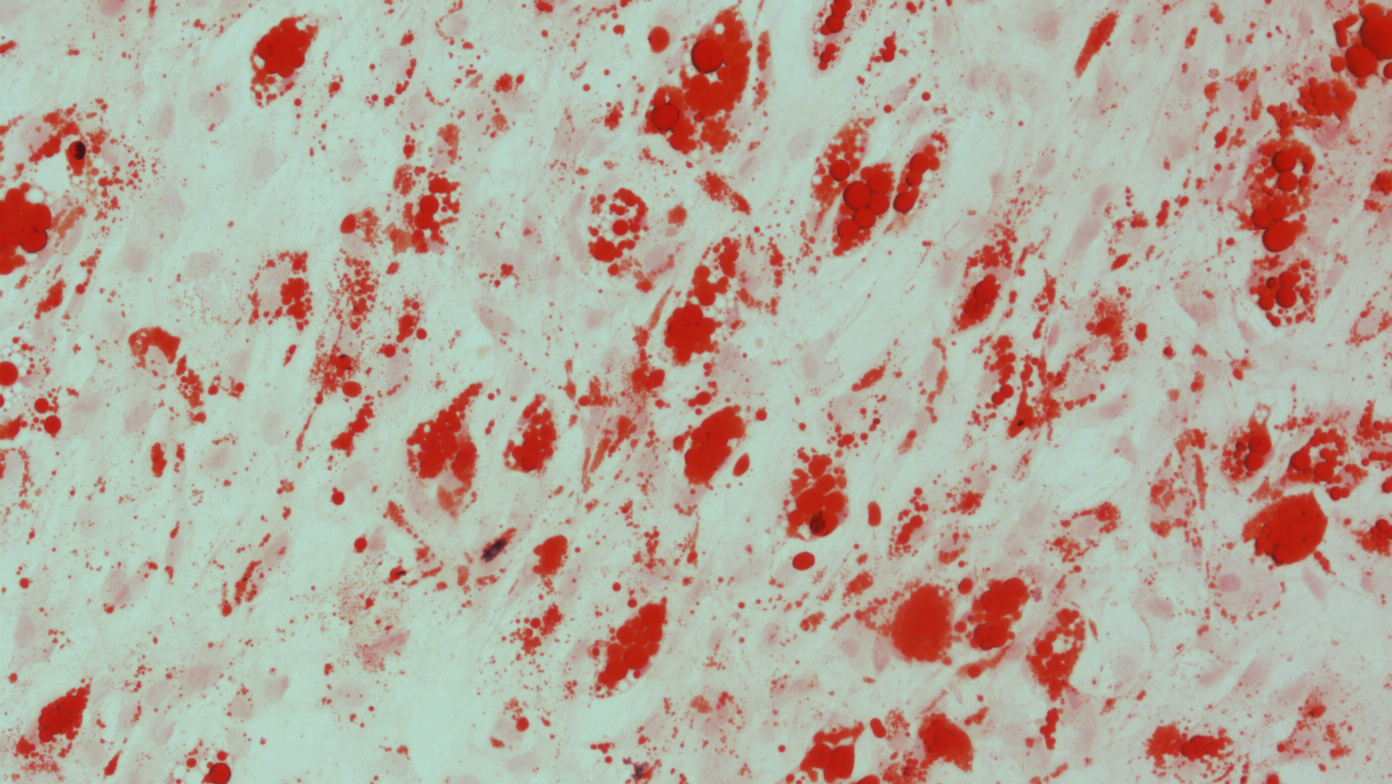

Supplement: S2 File — (ZIP) [file pone.0256484.s004.zip › S2 File/Figure 6B C COSTE.TIF]

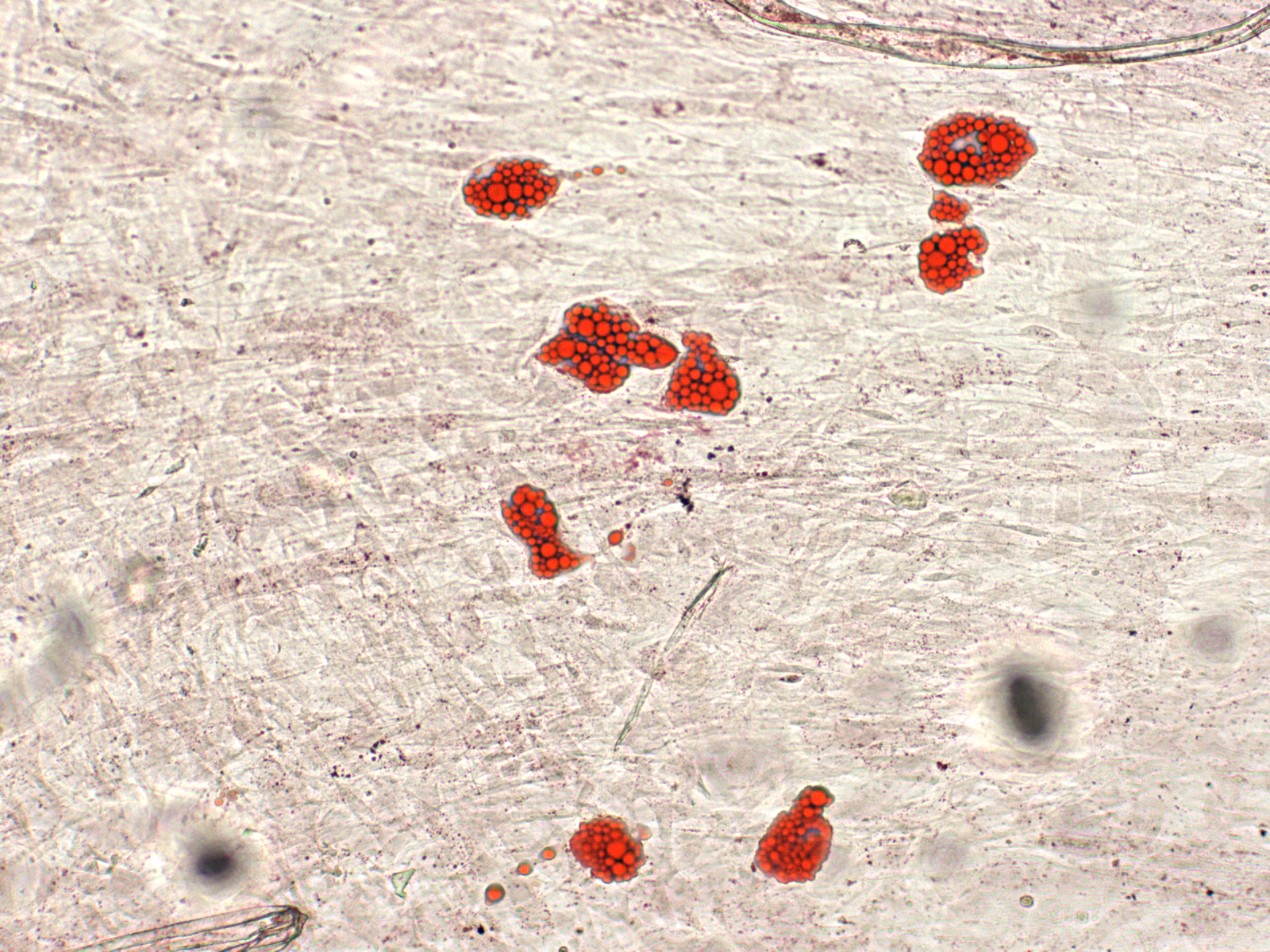

Supplement: S2 File — (ZIP) [file pone.0256484.s004.zip › S2 File/Figure 6C C Coste.tiff]

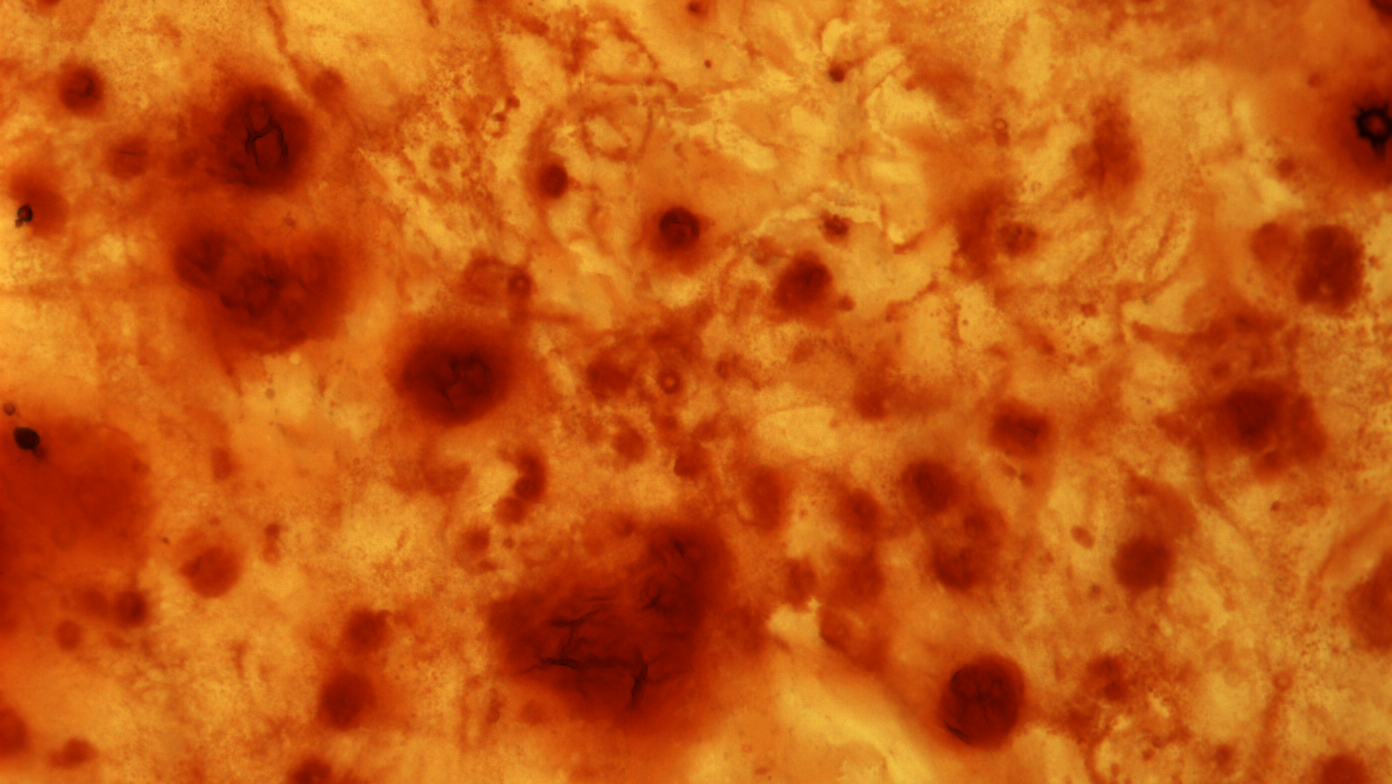

Supplement: S2 File — (ZIP) [file pone.0256484.s004.zip › S2 File/Figure 6D C COSTE.TIF]

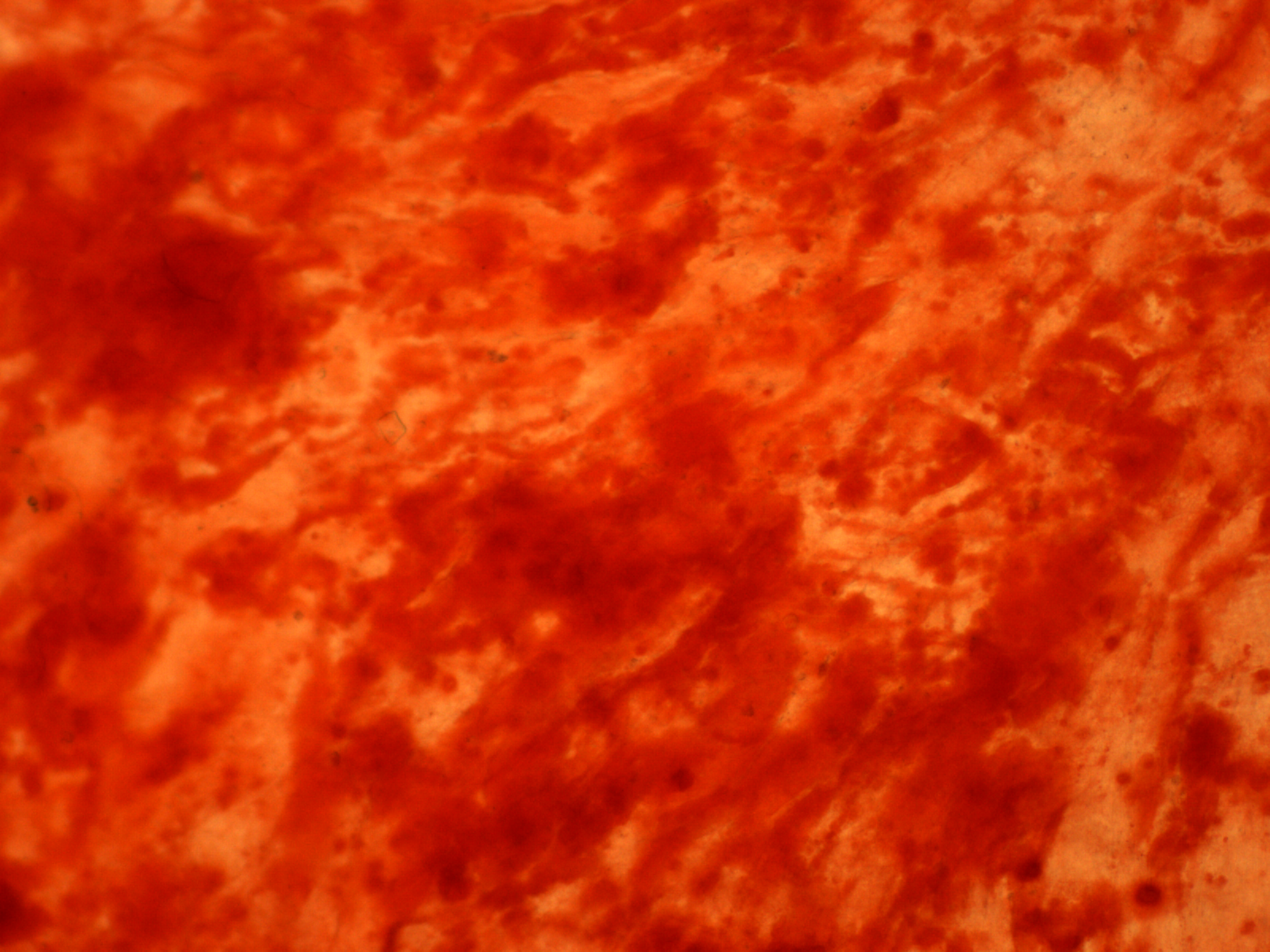

Supplement: S2 File — (ZIP) [file pone.0256484.s004.zip › S2 File/Figure 6E C Coste.tif]

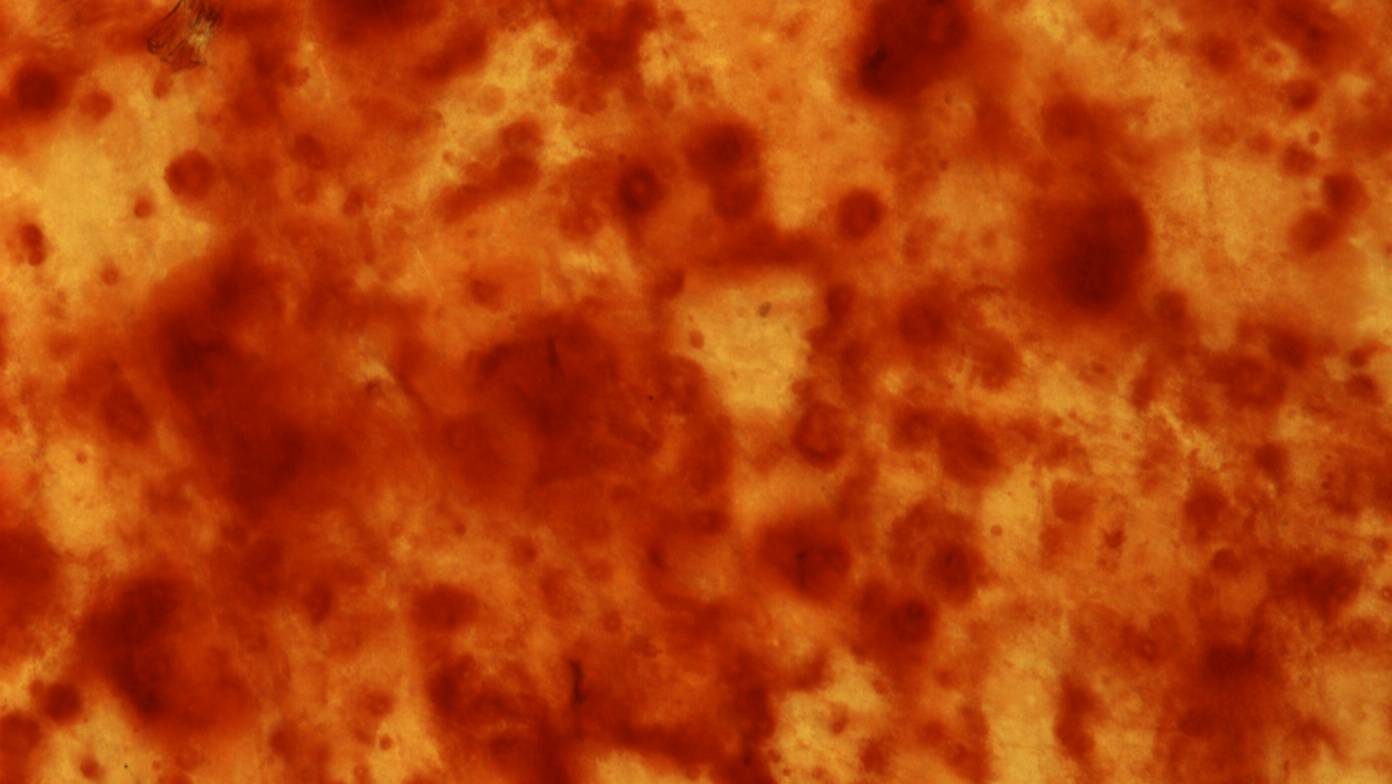

Supplement: S2 File — (ZIP) [file pone.0256484.s004.zip › S2 File/Figure 6F C Coste.TIF]

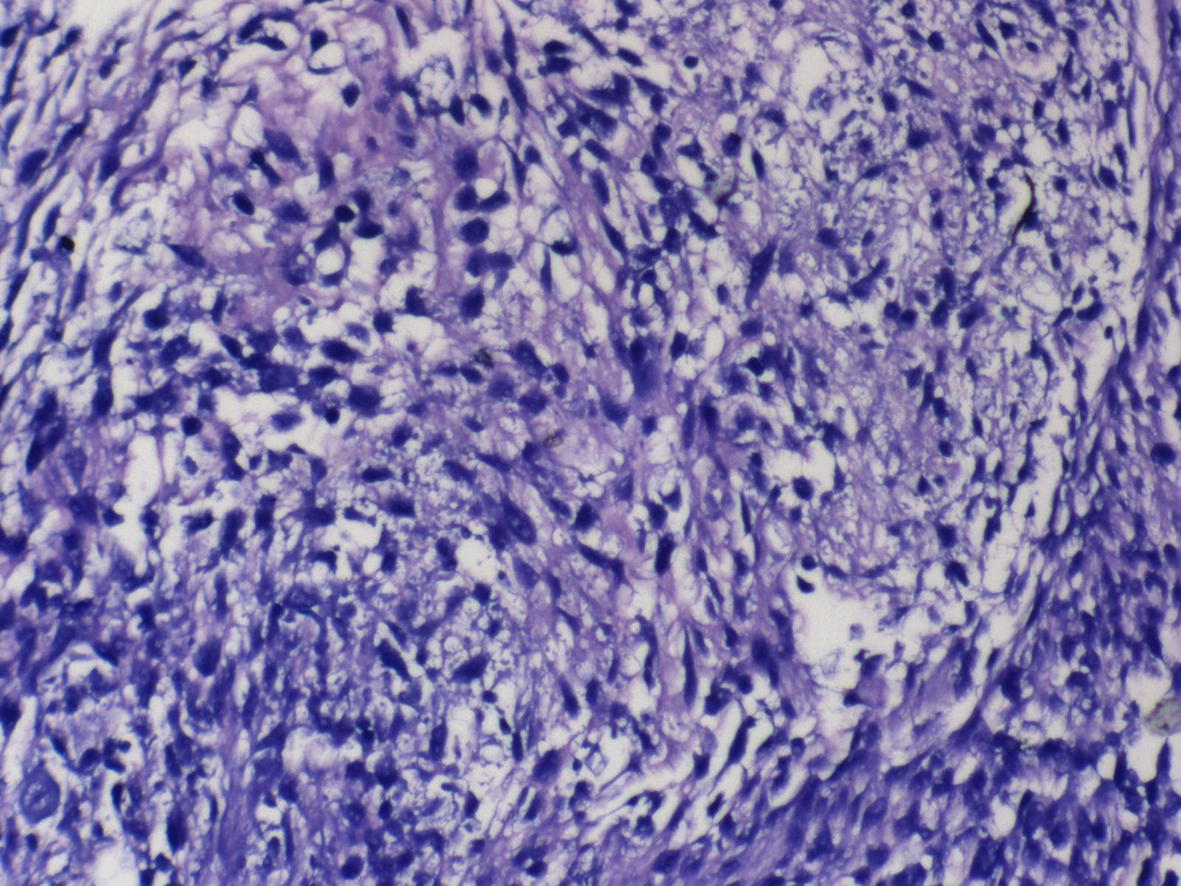

Supplement: S2 File — (ZIP) [file pone.0256484.s004.zip › S2 File/Figure 6I ATSC Chondro replacement.jpg]

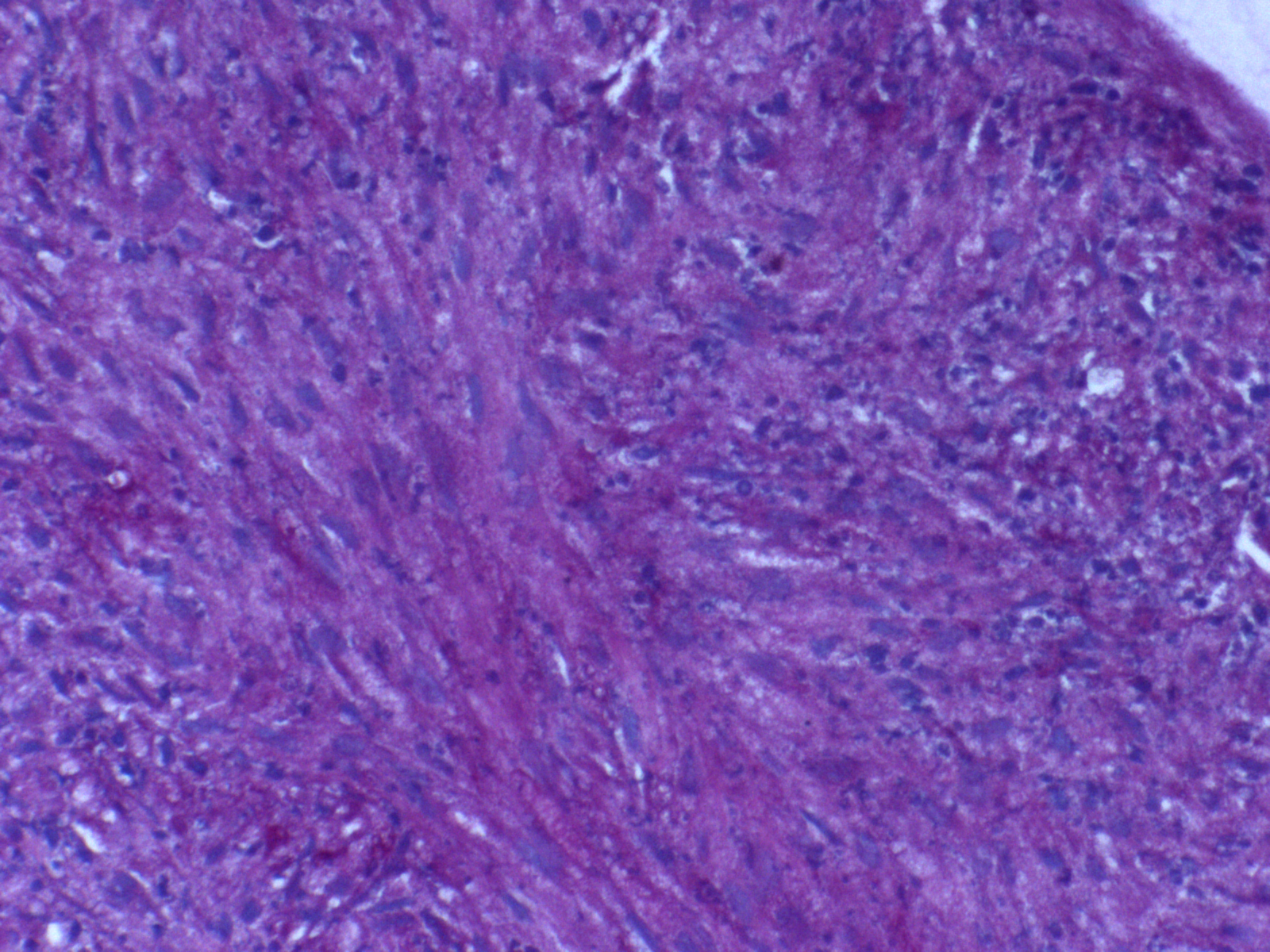

Supplement: S2 File — (ZIP) [file pone.0256484.s004.zip › S2 File/Figure 6I C Coste.tif]

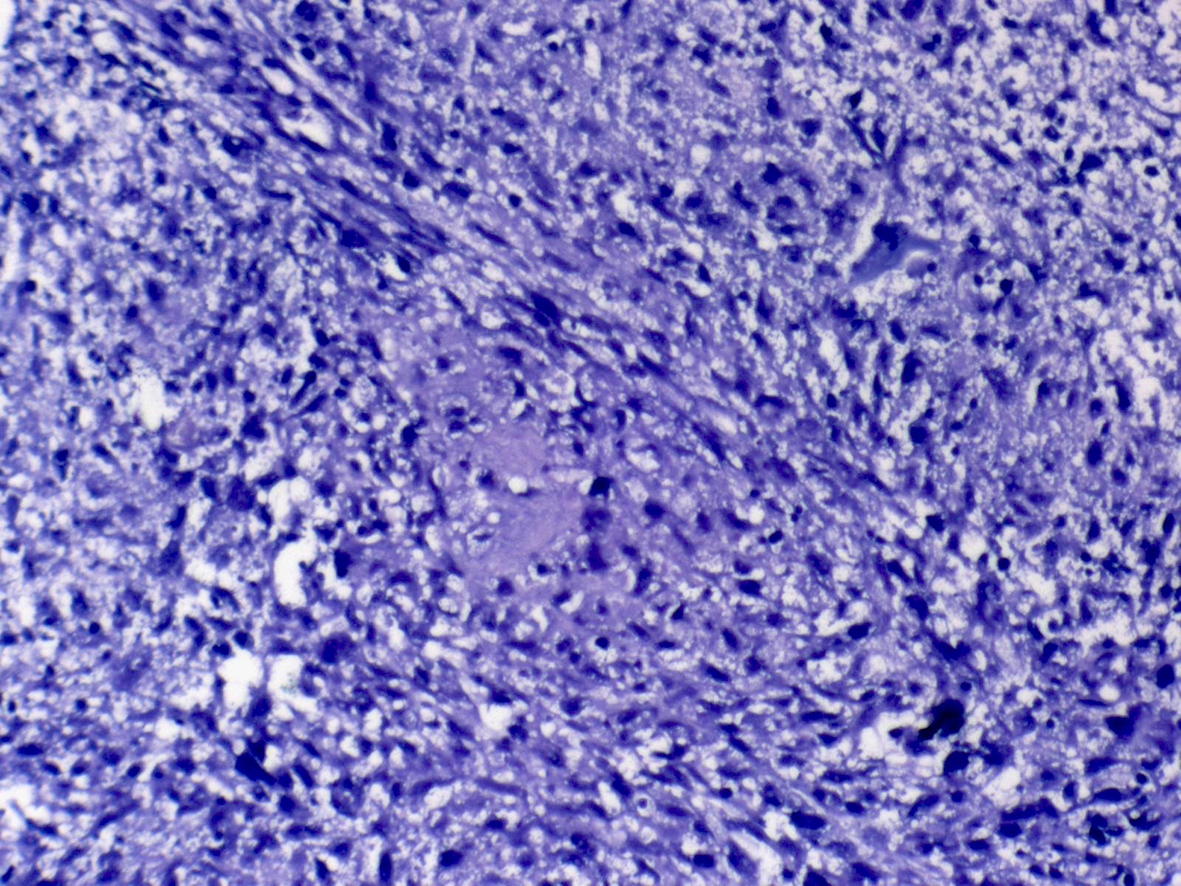

Supplement: S2 File — (ZIP) [file pone.0256484.s004.zip › S2 File/Figure 6J BMSC Chondro replacement.jpg]

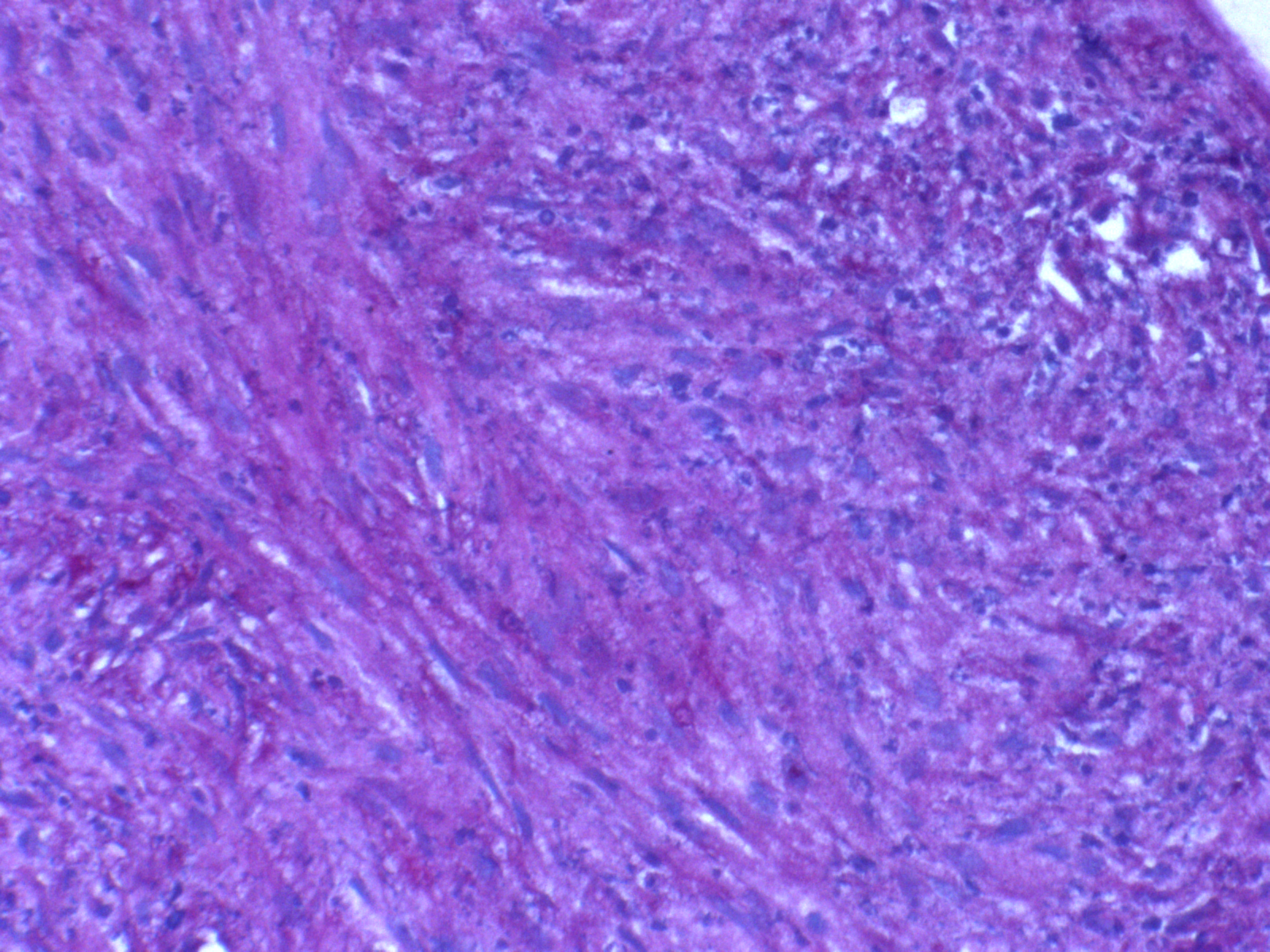

Supplement: S2 File — (ZIP) [file pone.0256484.s004.zip › S2 File/Figure 6J C Coste .tif]

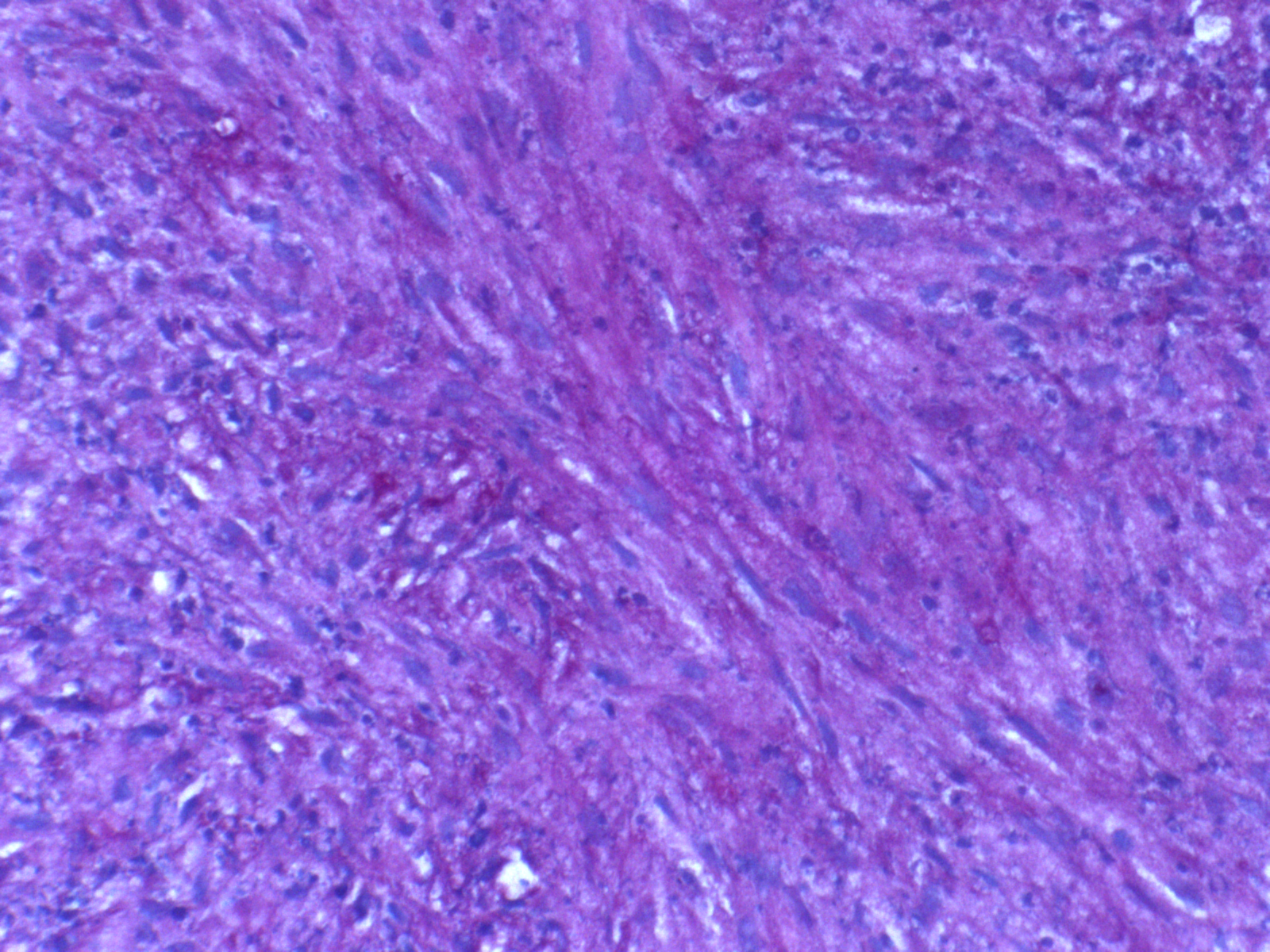

Supplement: S2 File — (ZIP) [file pone.0256484.s004.zip › S2 File/Figure 6K C Coste.tif]

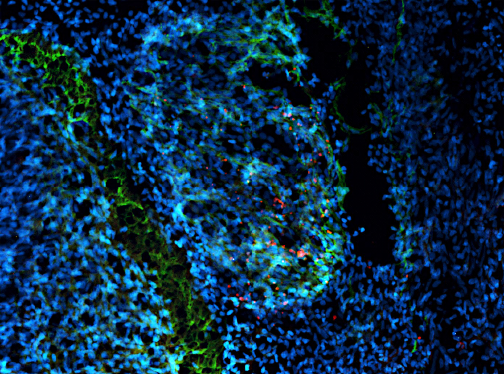

Supplement: S4 File — (ZIP) [file pone.0256484.s006.zip › S4 File/Figure S2A C Coste.tif]

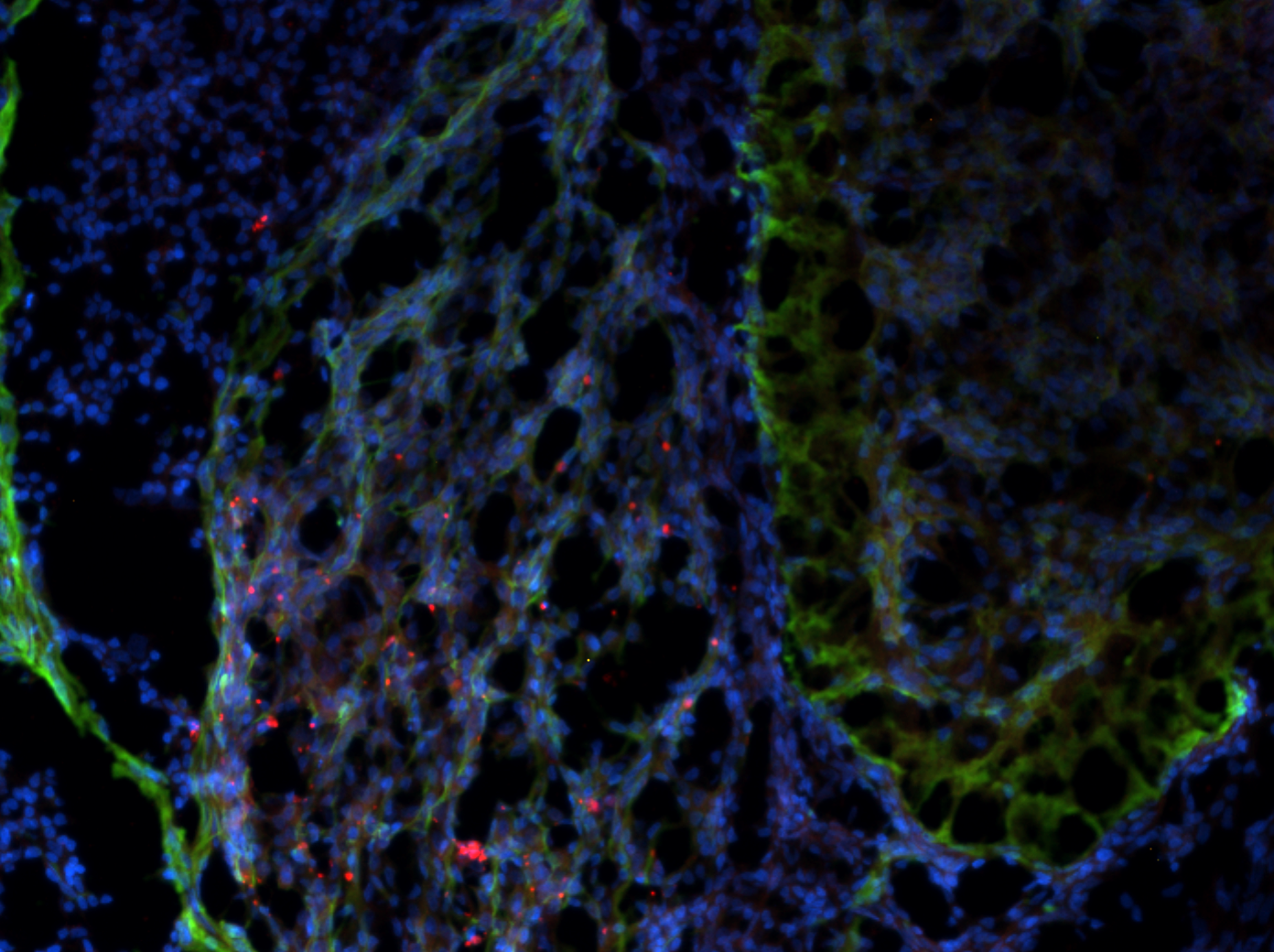

Supplement: S4 File — (ZIP) [file pone.0256484.s006.zip › S4 File/Figure S2B C Coste.tif]

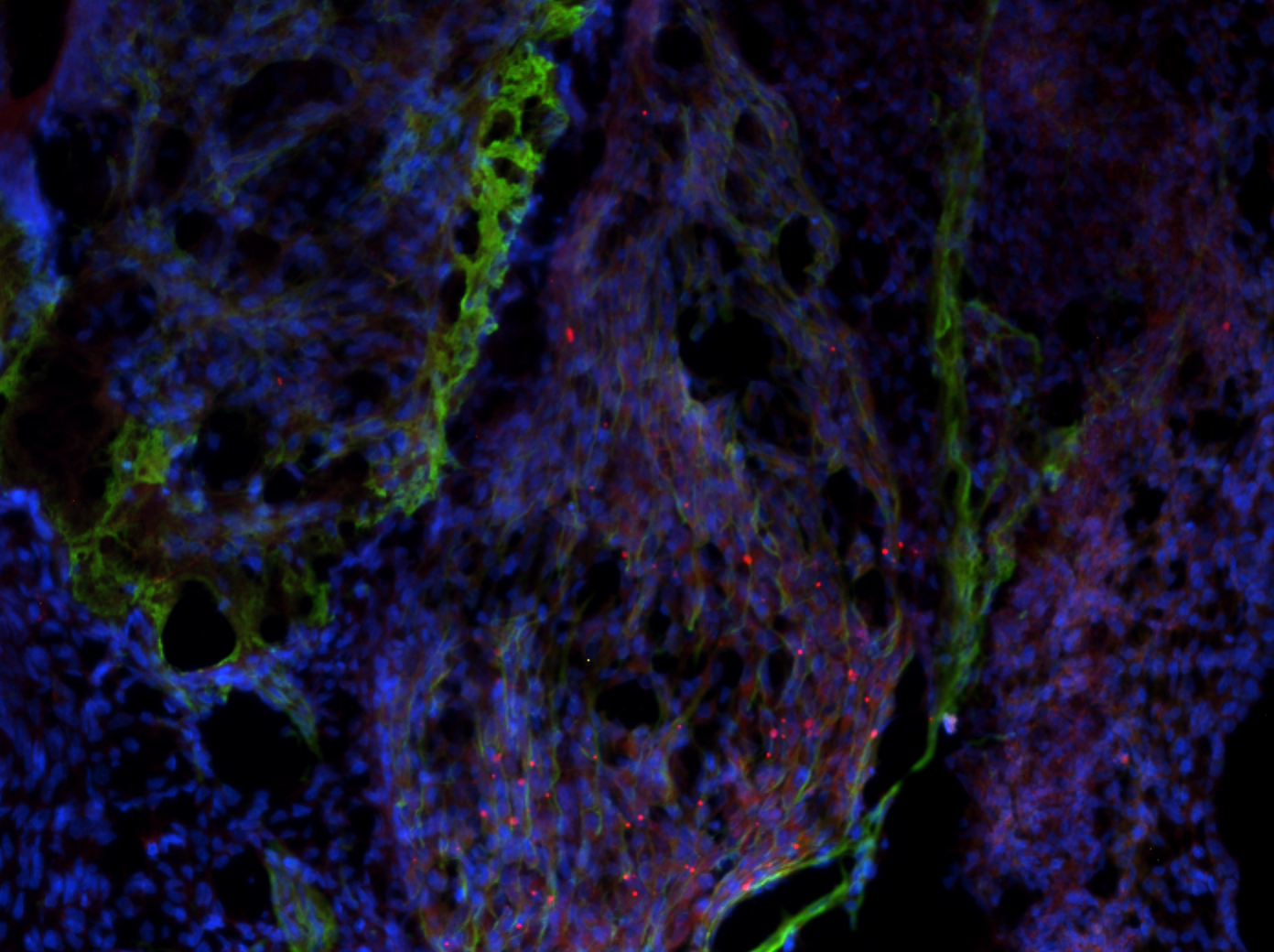

Supplement: S4 File — (ZIP) [file pone.0256484.s006.zip › S4 File/Figure S2C C Coste.tif]

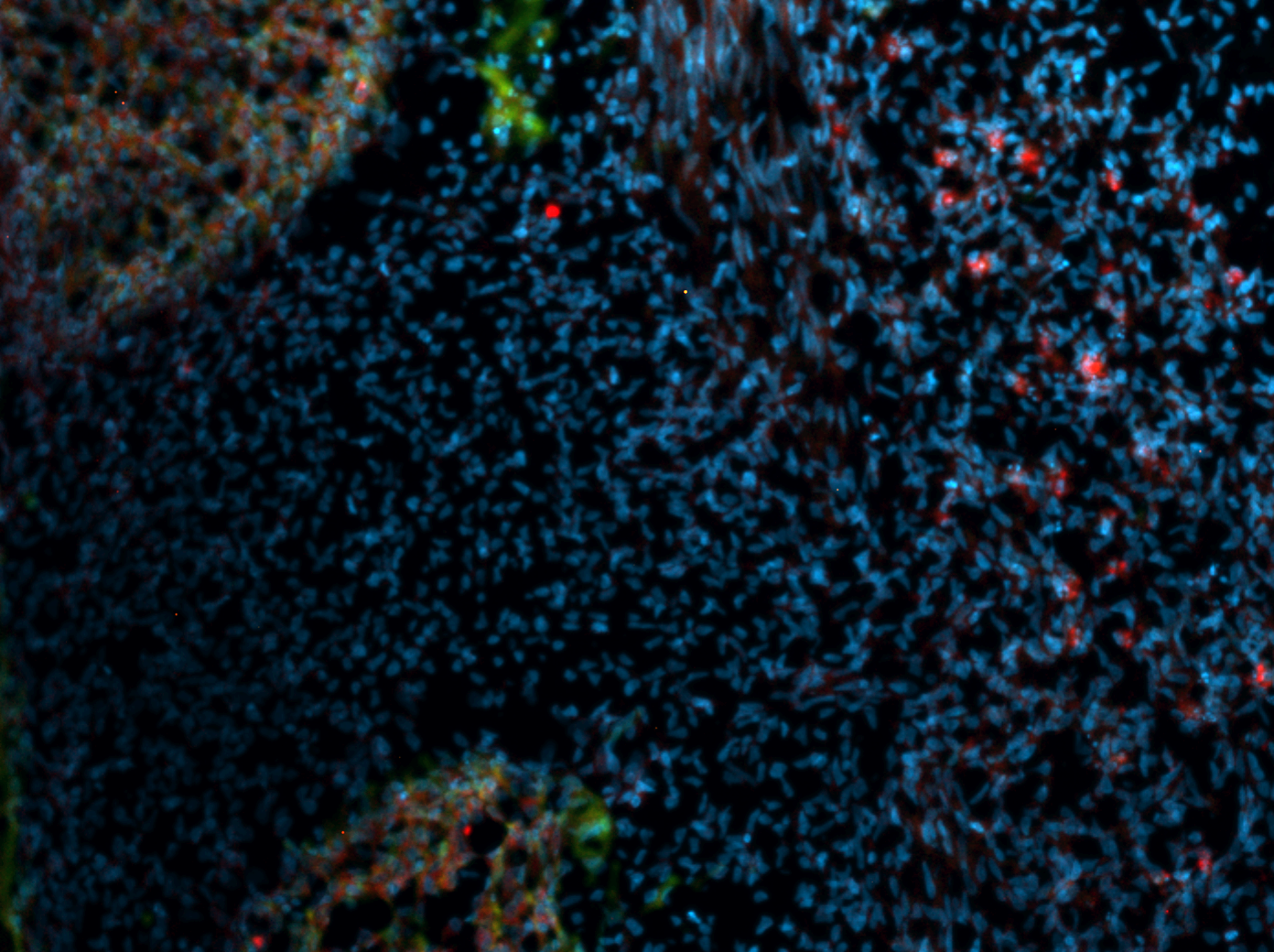

Supplement: S4 File — (ZIP) [file pone.0256484.s006.zip › S4 File/Figure S2D C Coste.tif]

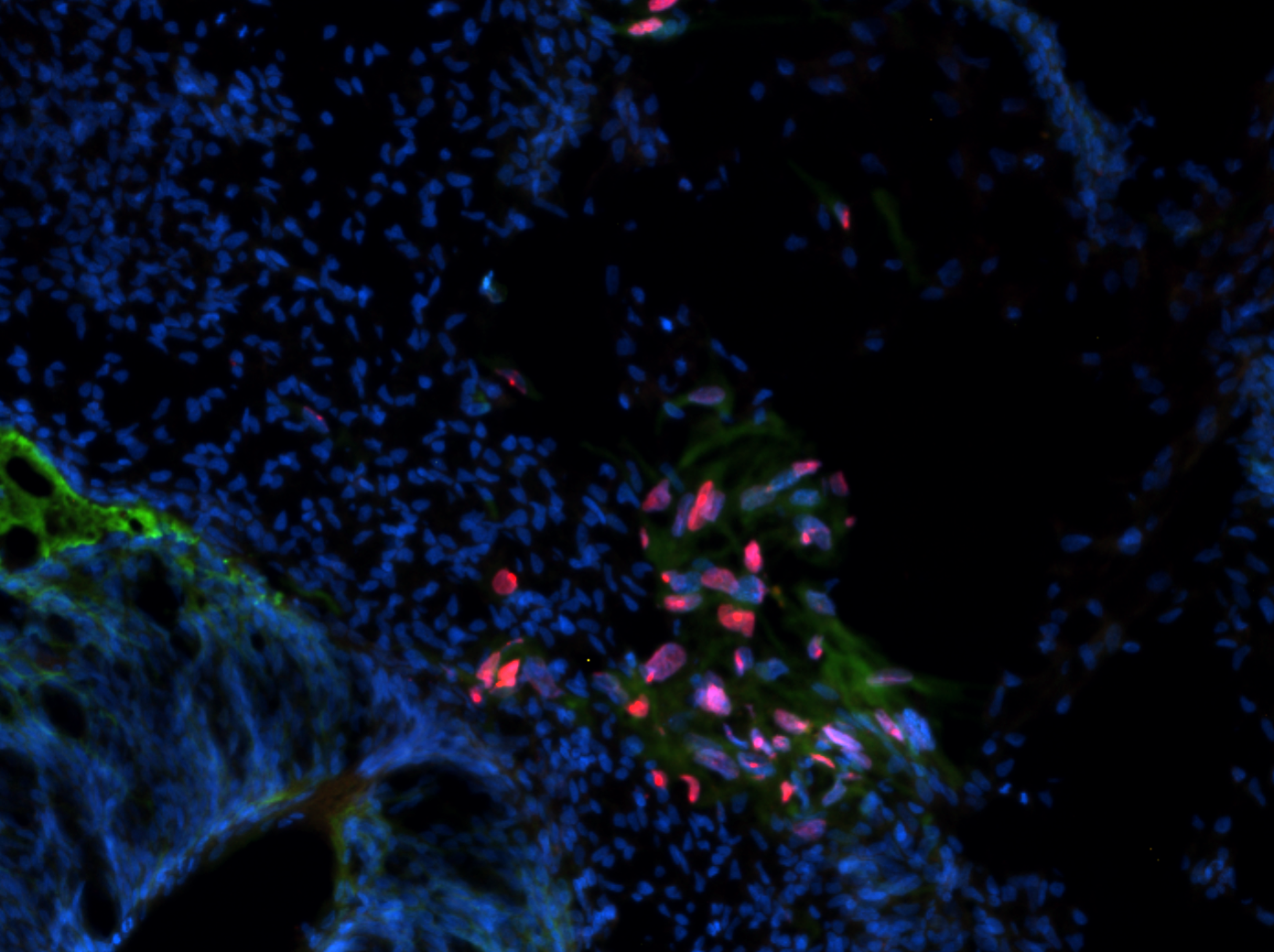

Supplement: S4 File — (ZIP) [file pone.0256484.s006.zip › S4 File/Figure S2E C Coste.tif]

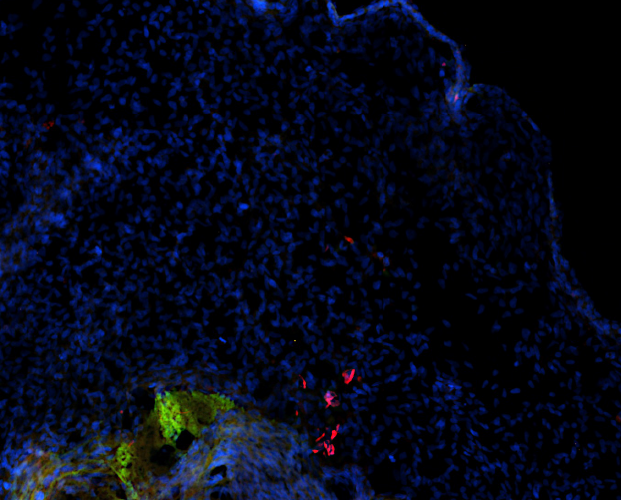

Supplement: S4 File — (ZIP) [file pone.0256484.s006.zip › S4 File/Figure S2F C Coste.tif]

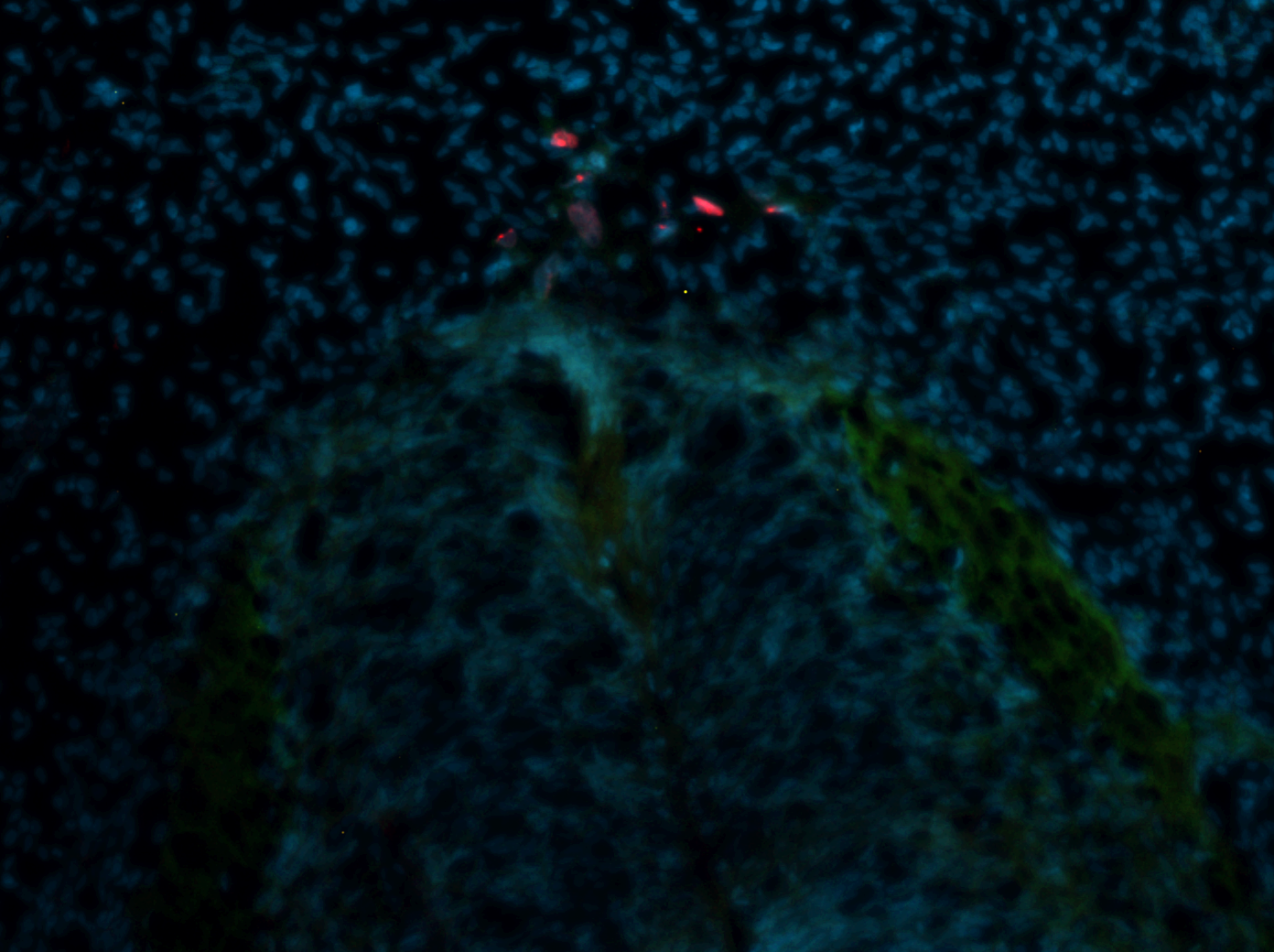

Supplement: S4 File — (ZIP) [file pone.0256484.s006.zip › S4 File/Figure S2G C Coste.tif]

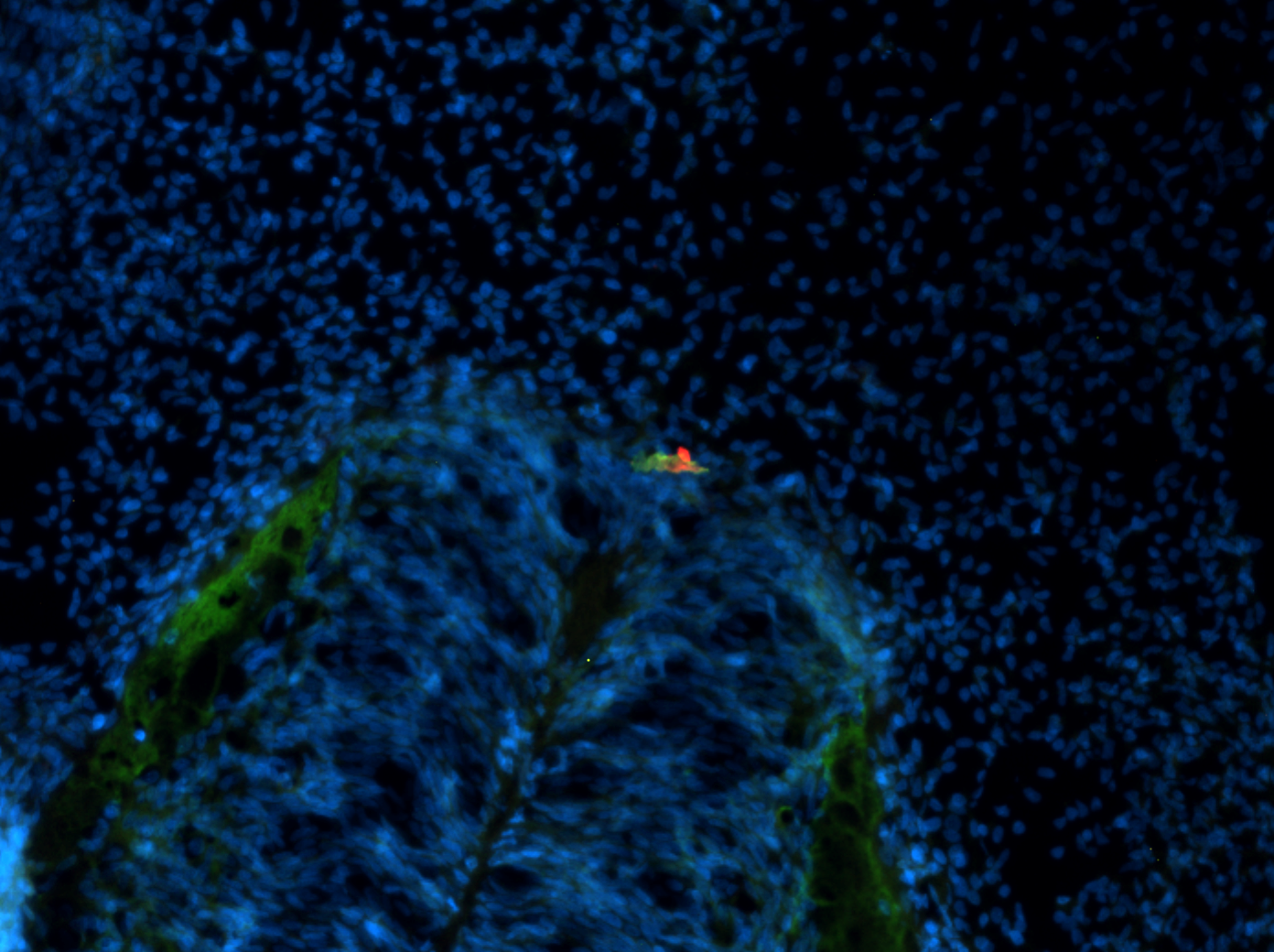

Supplement: S4 File — (ZIP) [file pone.0256484.s006.zip › S4 File/Figure S2H C Coste.tif]

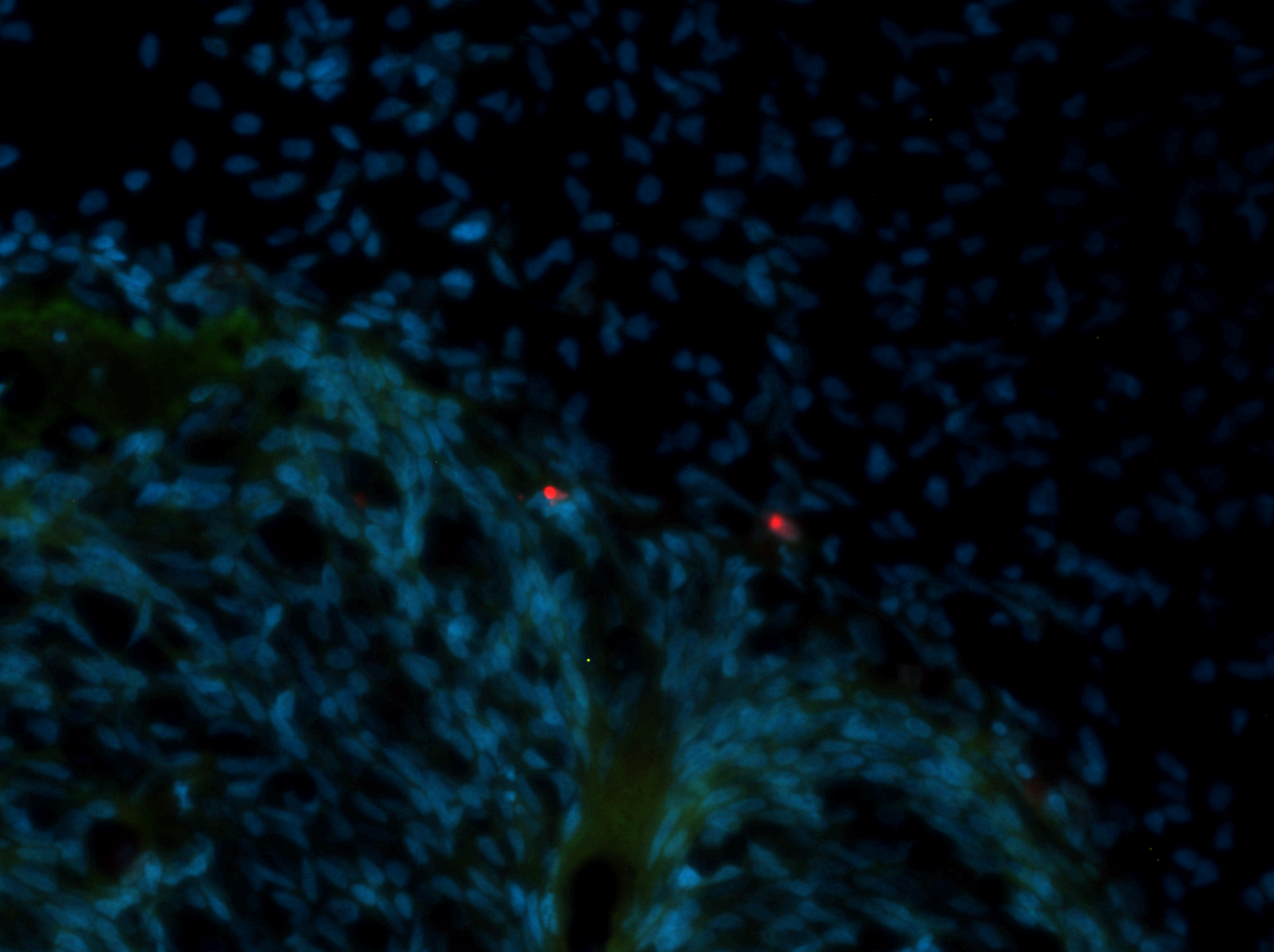

Supplement: S4 File — (ZIP) [file pone.0256484.s006.zip › S4 File/Figure S2I C Coste.tif]

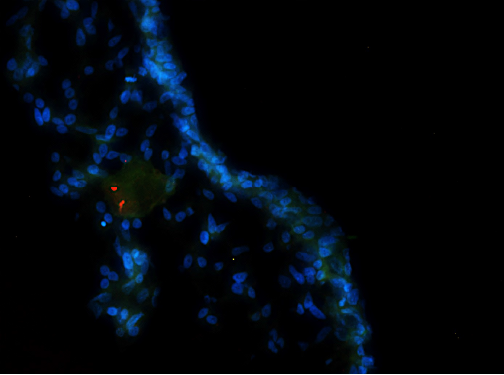

Supplement: S4 File — (ZIP) [file pone.0256484.s006.zip › S4 File/Figure S2J C Coste.tif]

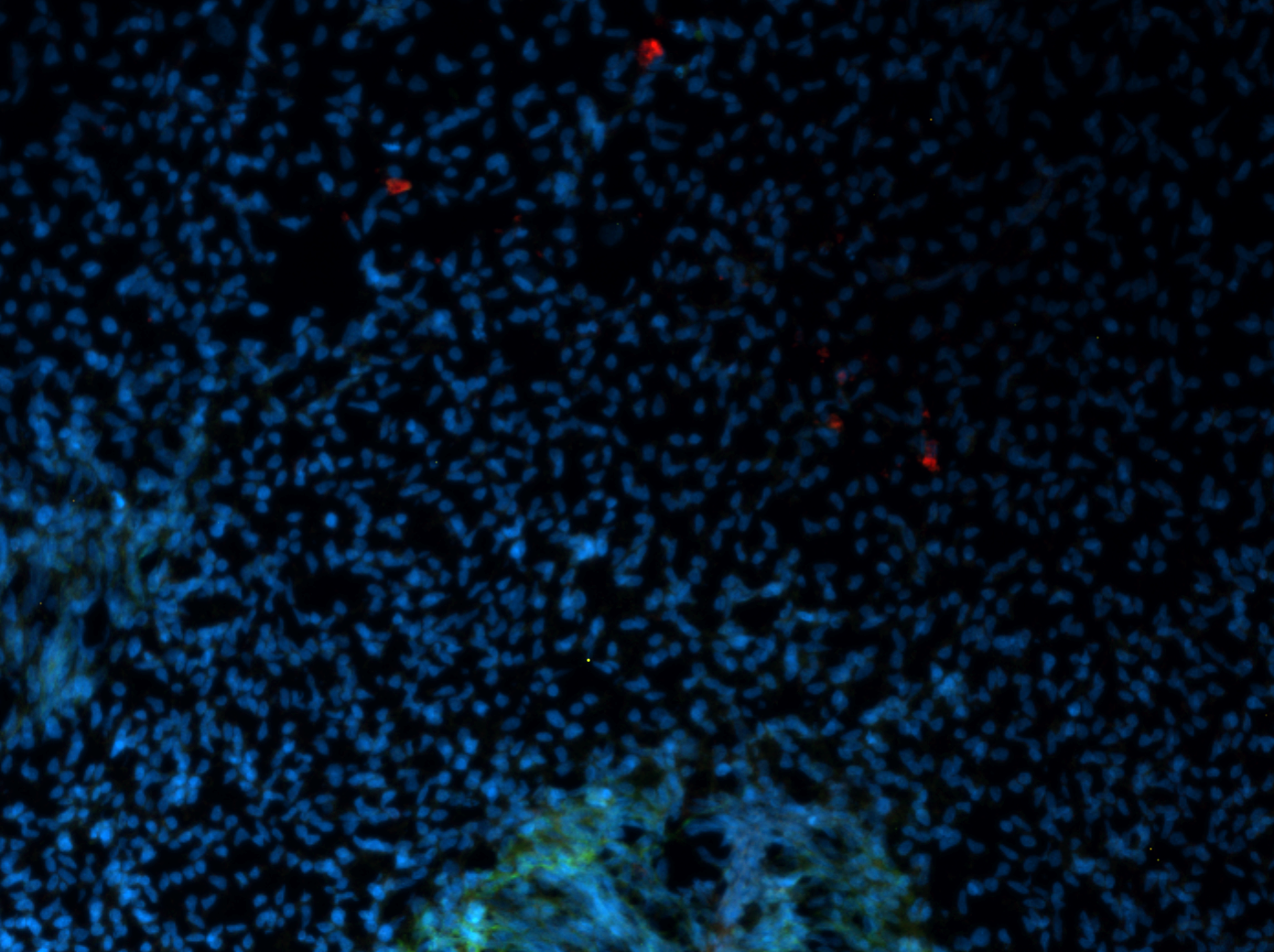

Supplement: S4 File — (ZIP) [file pone.0256484.s006.zip › S4 File/Figure S2K C Coste.tif]

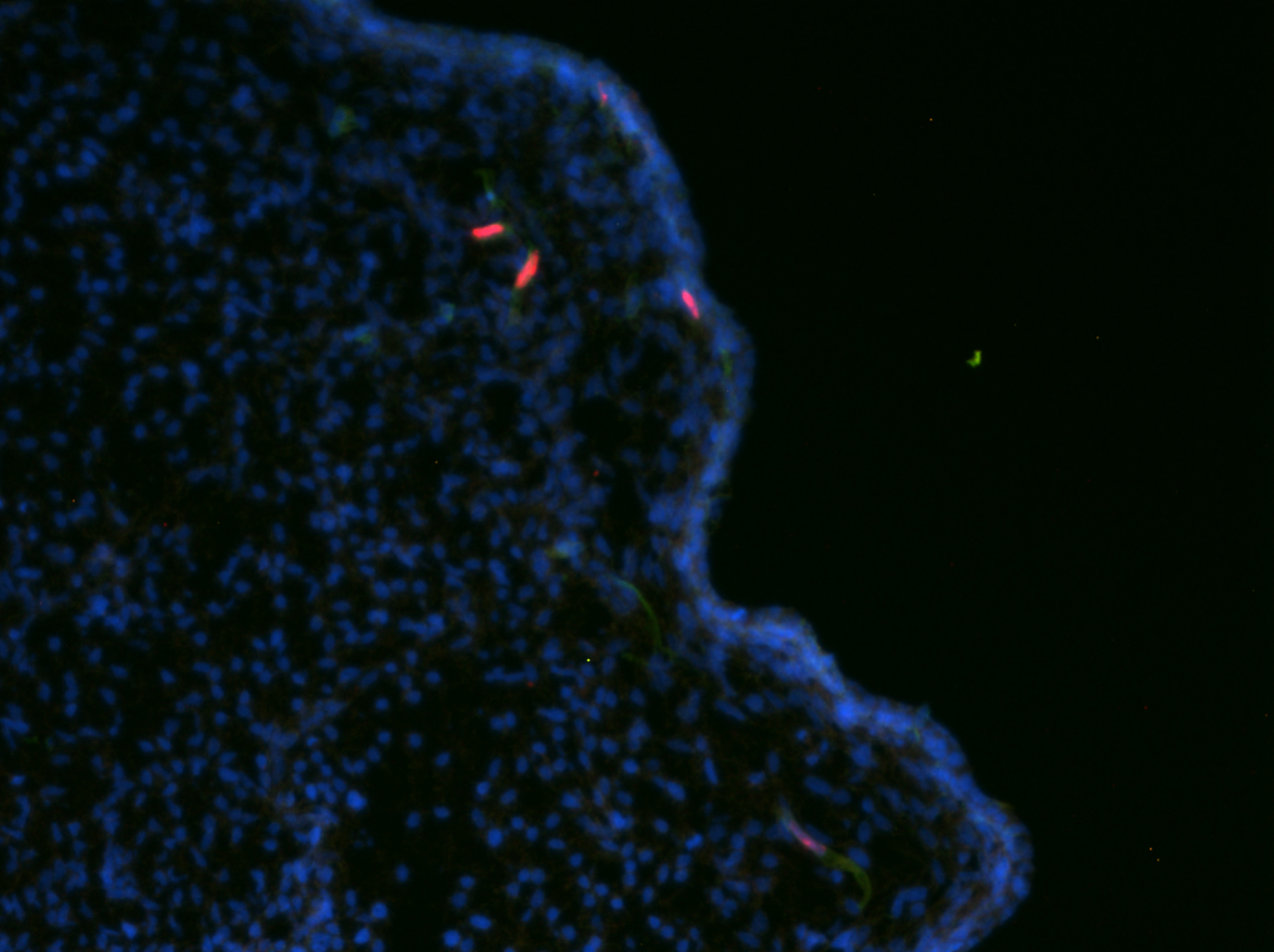

Supplement: S4 File — (ZIP) [file pone.0256484.s006.zip › S4 File/Figure S2L C Coste.tif]

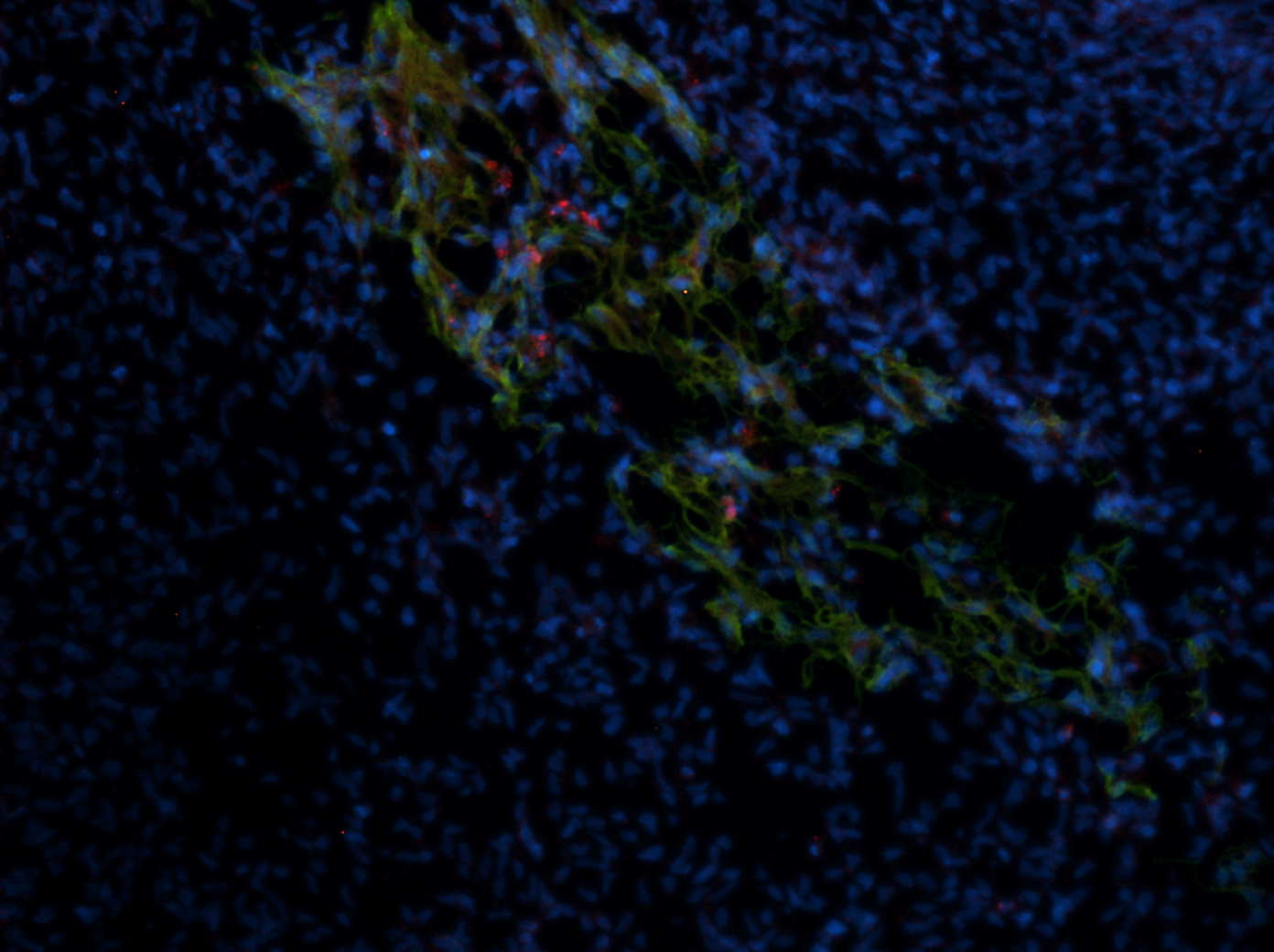

Supplement: S4 File — (ZIP) [file pone.0256484.s006.zip › S4 File/Figure S2M C Coste.tif]

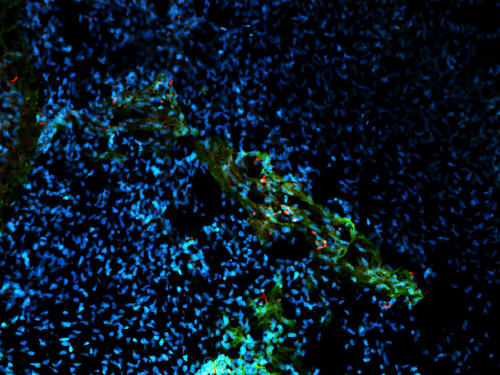

Supplement: S4 File — (ZIP) [file pone.0256484.s006.zip › S4 File/Figure S2N C Coste.tif]

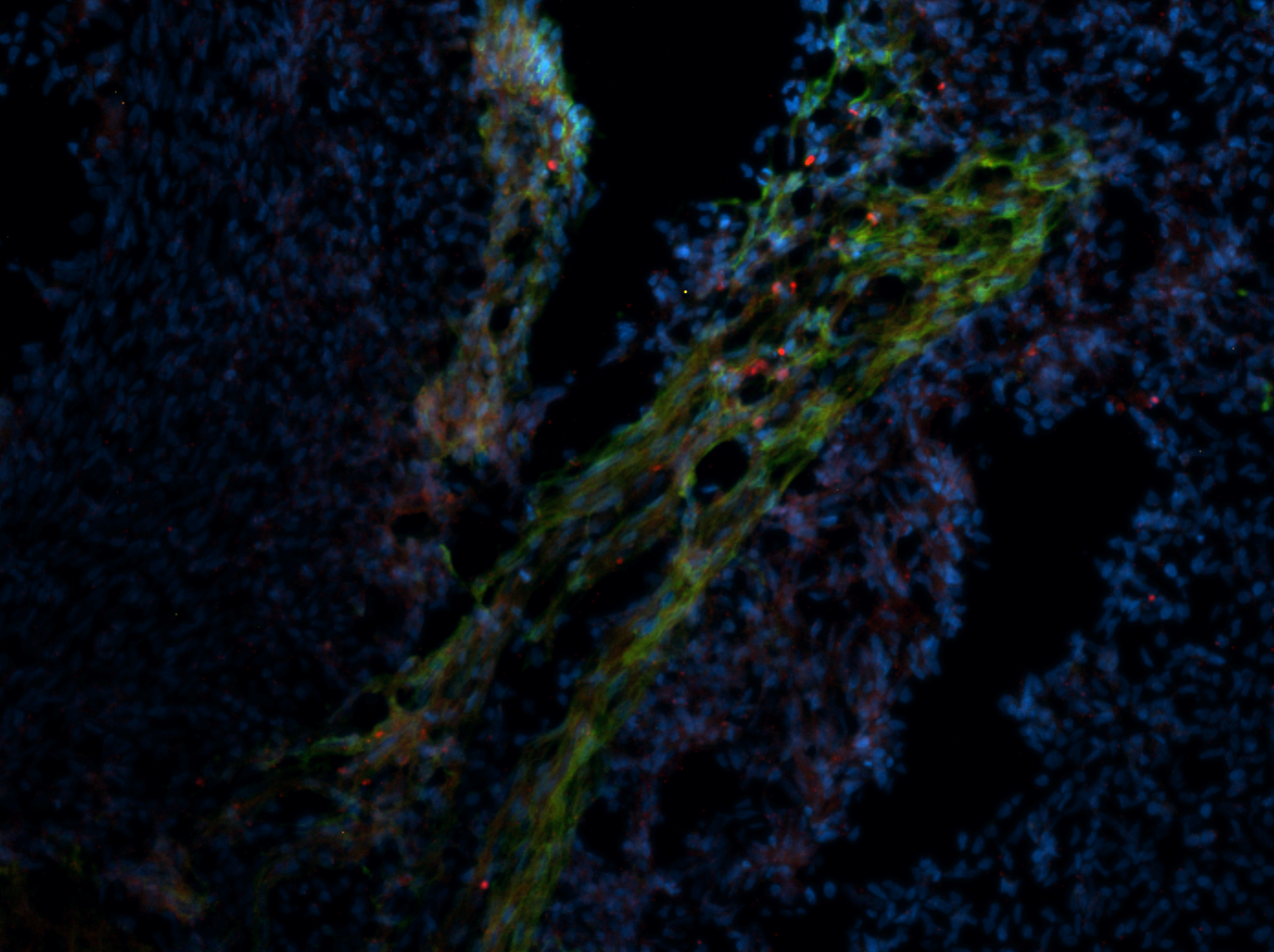

Supplement: S4 File — (ZIP) [file pone.0256484.s006.zip › S4 File/Figure S2O C Coste.tif]

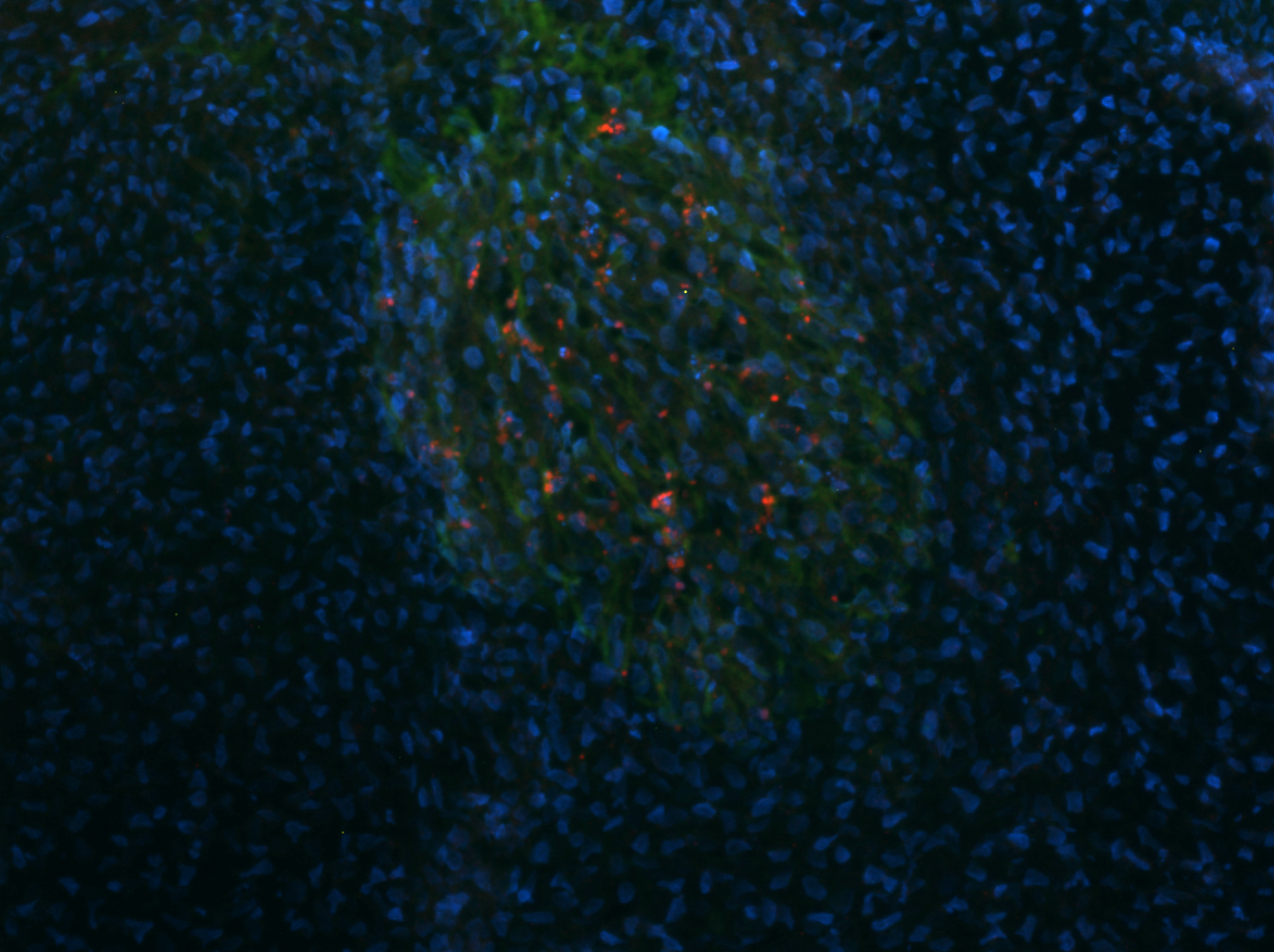

Supplement: S5 File — (ZIP) [file pone.0256484.s007.zip › S5 File/Figure S1A C Coste.tif]

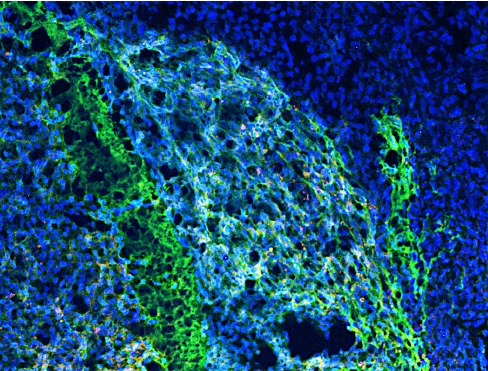

Supplement: S5 File — (ZIP) [file pone.0256484.s007.zip › S5 File/Figure S1B C Coste.tif]

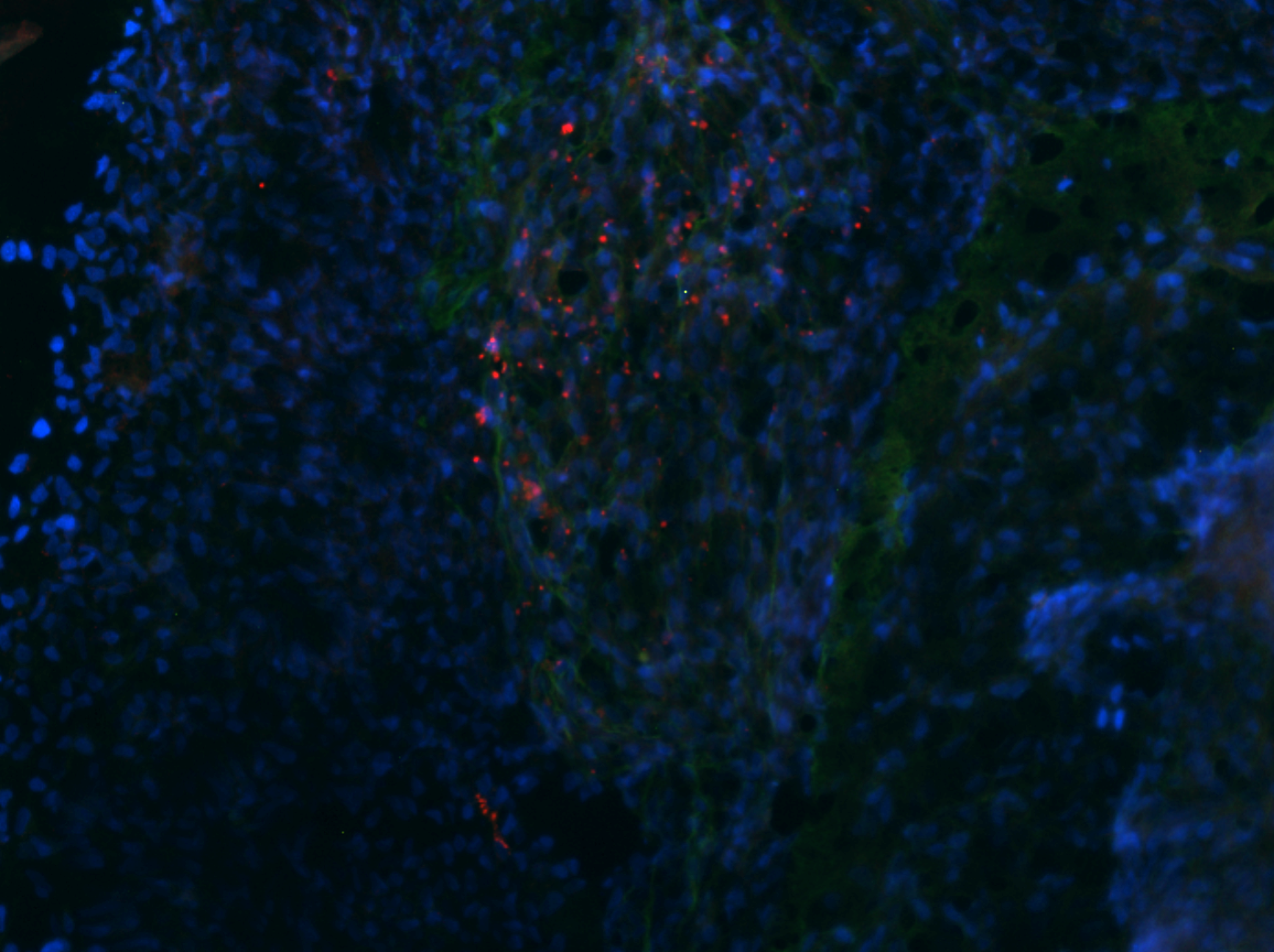

Supplement: S5 File — (ZIP) [file pone.0256484.s007.zip › S5 File/Figure S1C C Coste.tif]

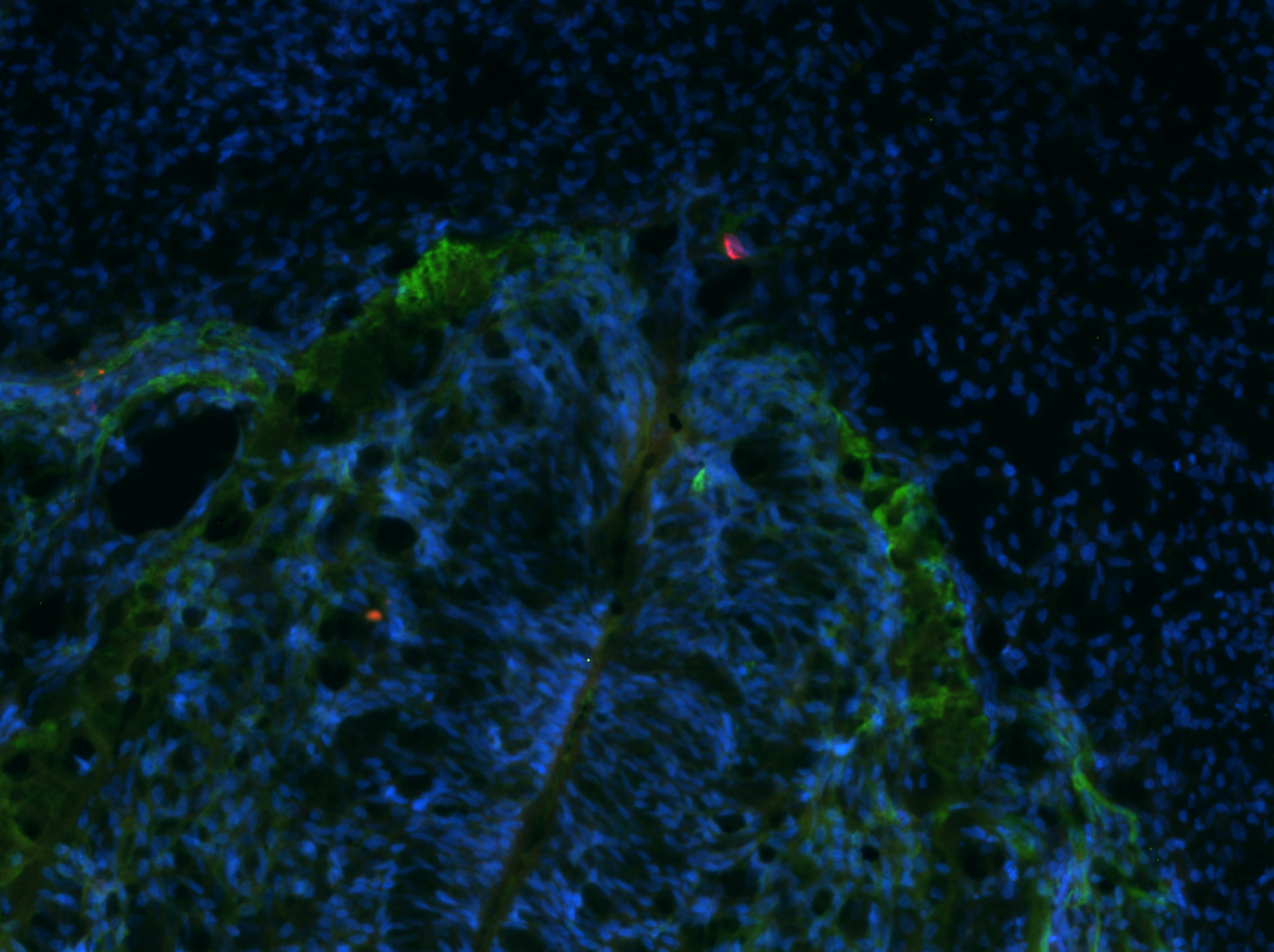

Supplement: S5 File — (ZIP) [file pone.0256484.s007.zip › S5 File/Figure S1D C Coste.tif]

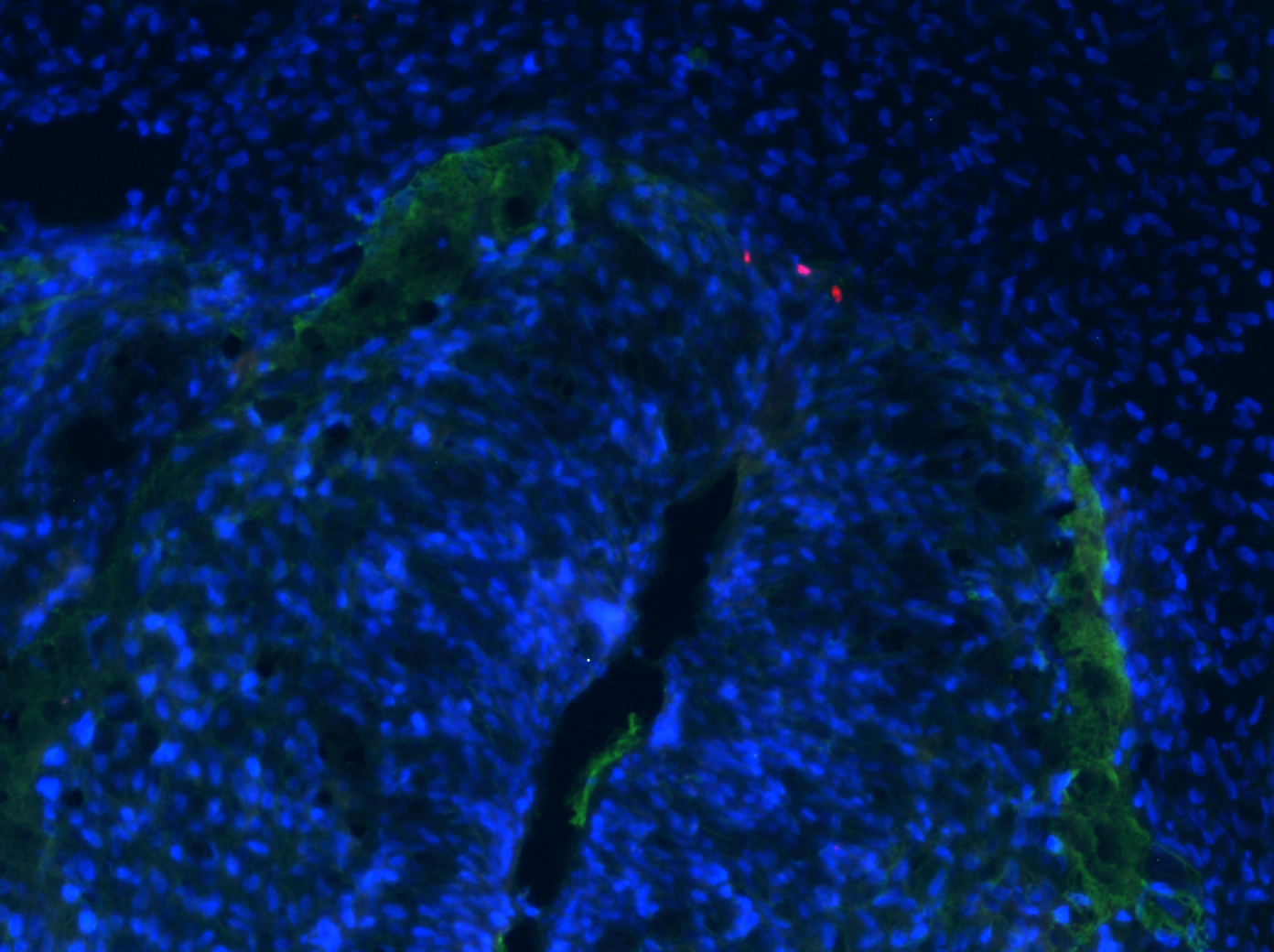

Supplement: S5 File — (ZIP) [file pone.0256484.s007.zip › S5 File/Figure S1F C Coste.tif]

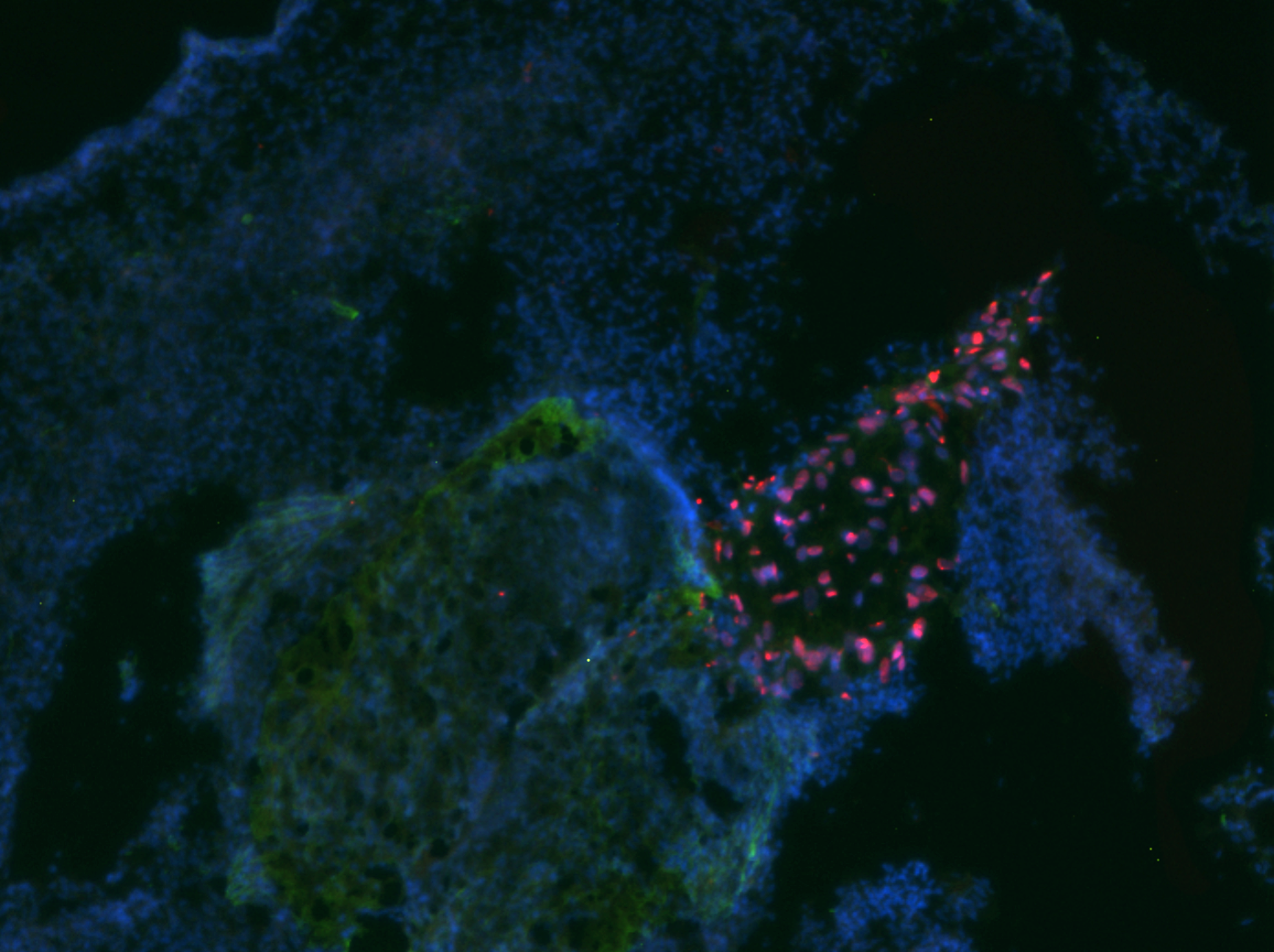

Supplement: S5 File — (ZIP) [file pone.0256484.s007.zip › S5 File/Figure S1G C Coste.tif]

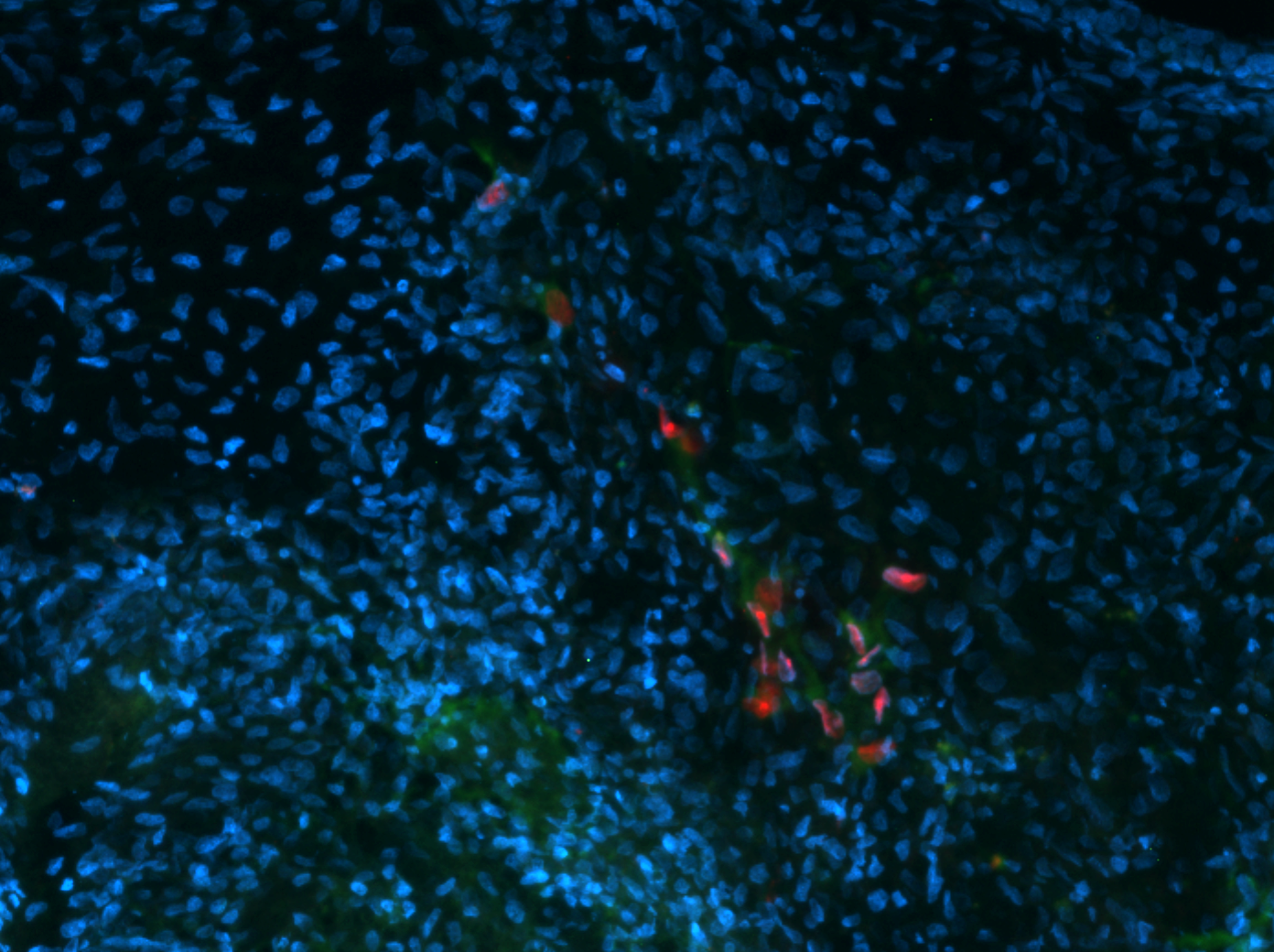

Supplement: S5 File — (ZIP) [file pone.0256484.s007.zip › S5 File/Figure S1H C Coste.tif]

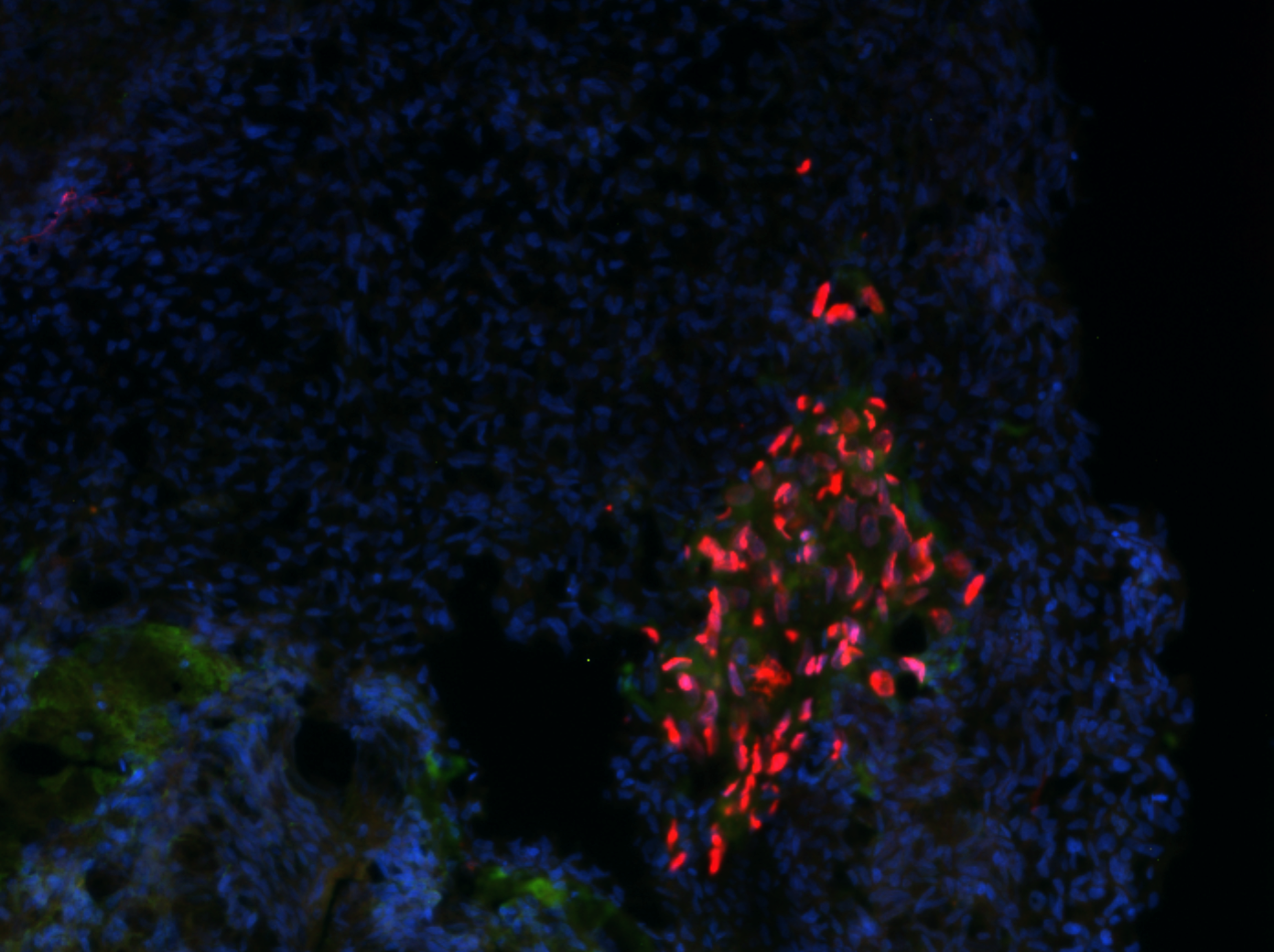

Supplement: S5 File — (ZIP) [file pone.0256484.s007.zip › S5 File/Figure S1I C Coste.tif]

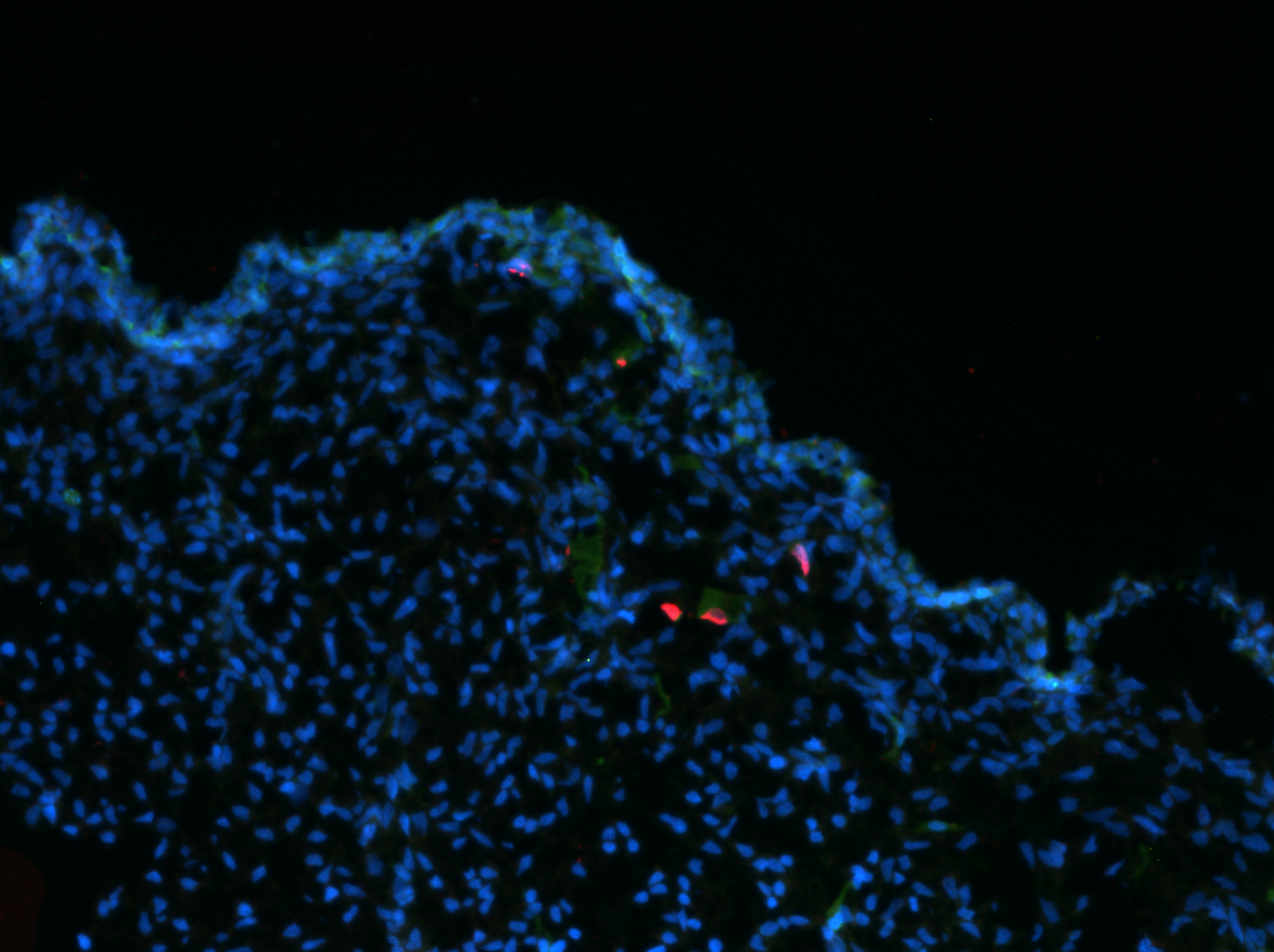

Supplement: S5 File — (ZIP) [file pone.0256484.s007.zip › S5 File/Figure S1J C Coste.tif]

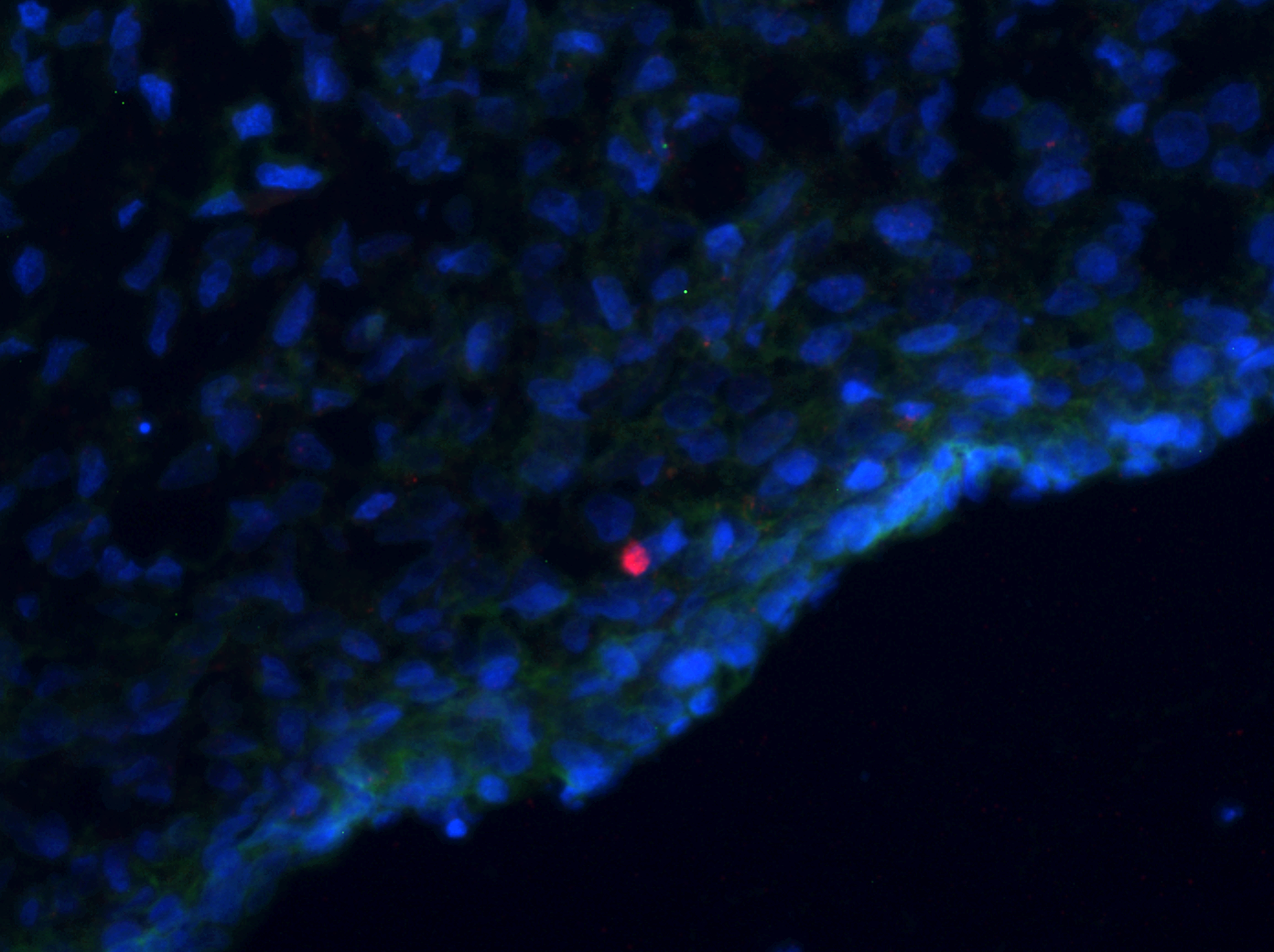

Supplement: S5 File — (ZIP) [file pone.0256484.s007.zip › S5 File/Figure S1K C Coste.tif]

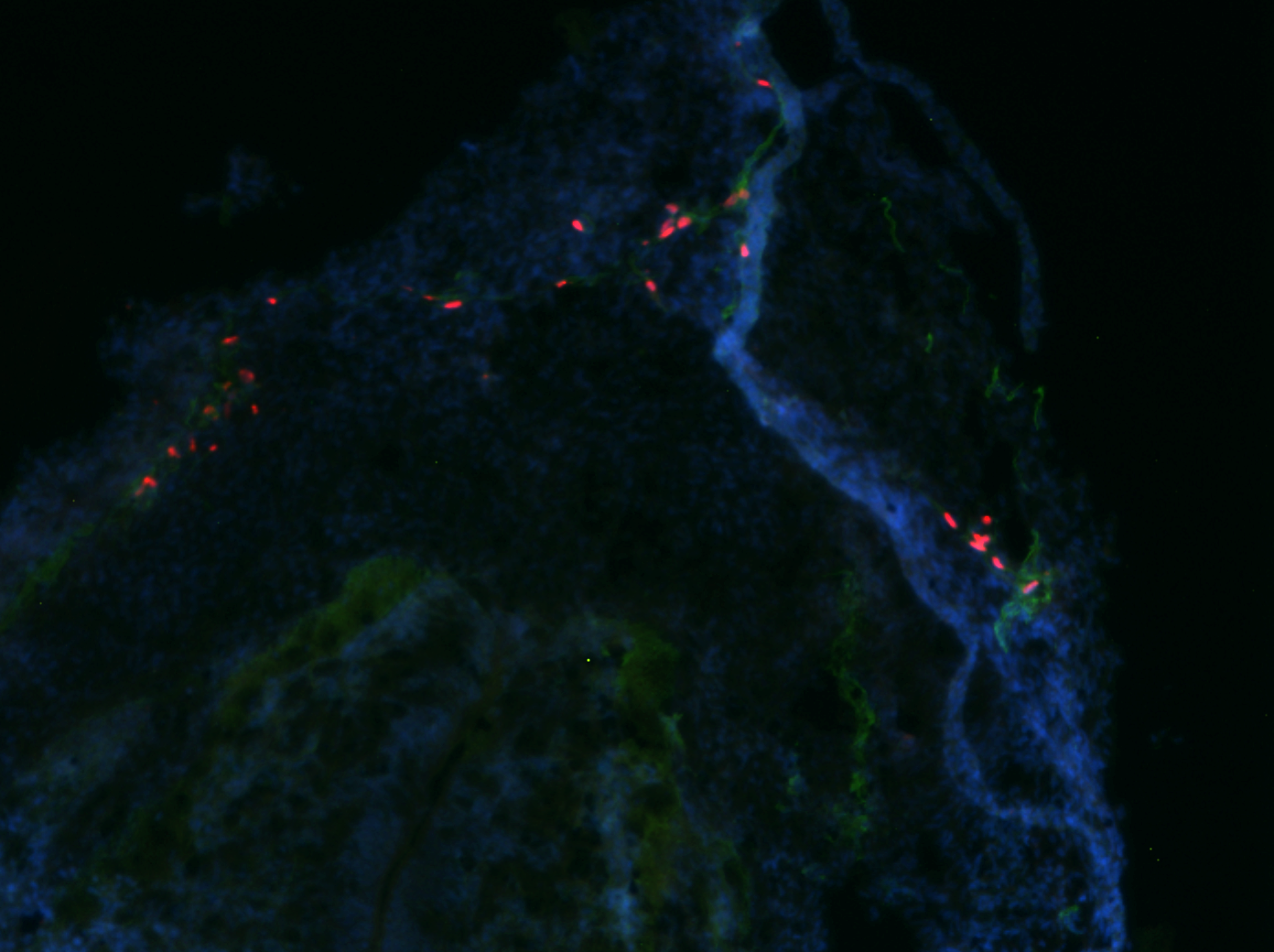

Supplement: S5 File — (ZIP) [file pone.0256484.s007.zip › S5 File/Figure S1L C Coste.tif]

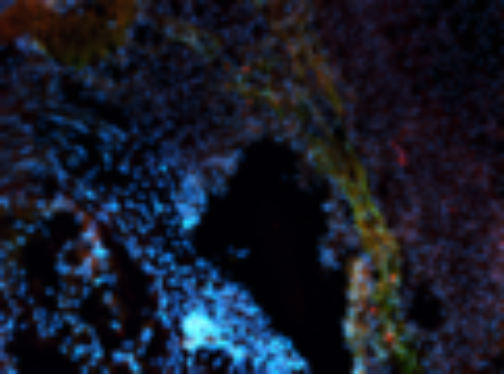

Supplement: S5 File — (ZIP) [file pone.0256484.s007.zip › S5 File/Figure S1M C Coste.tif]

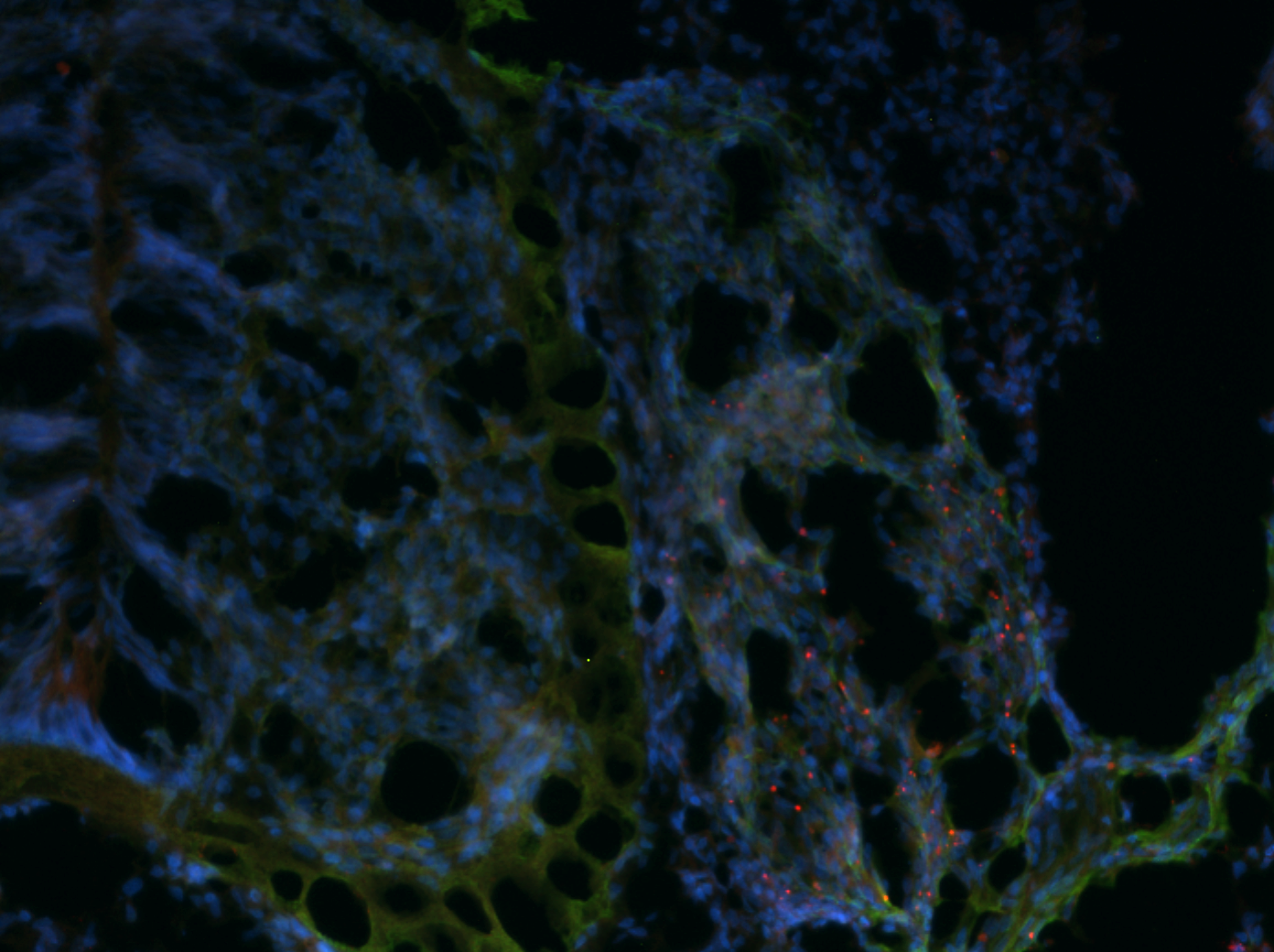

Supplement: S5 File — (ZIP) [file pone.0256484.s007.zip › S5 File/Figure S1N C Coste.tif]

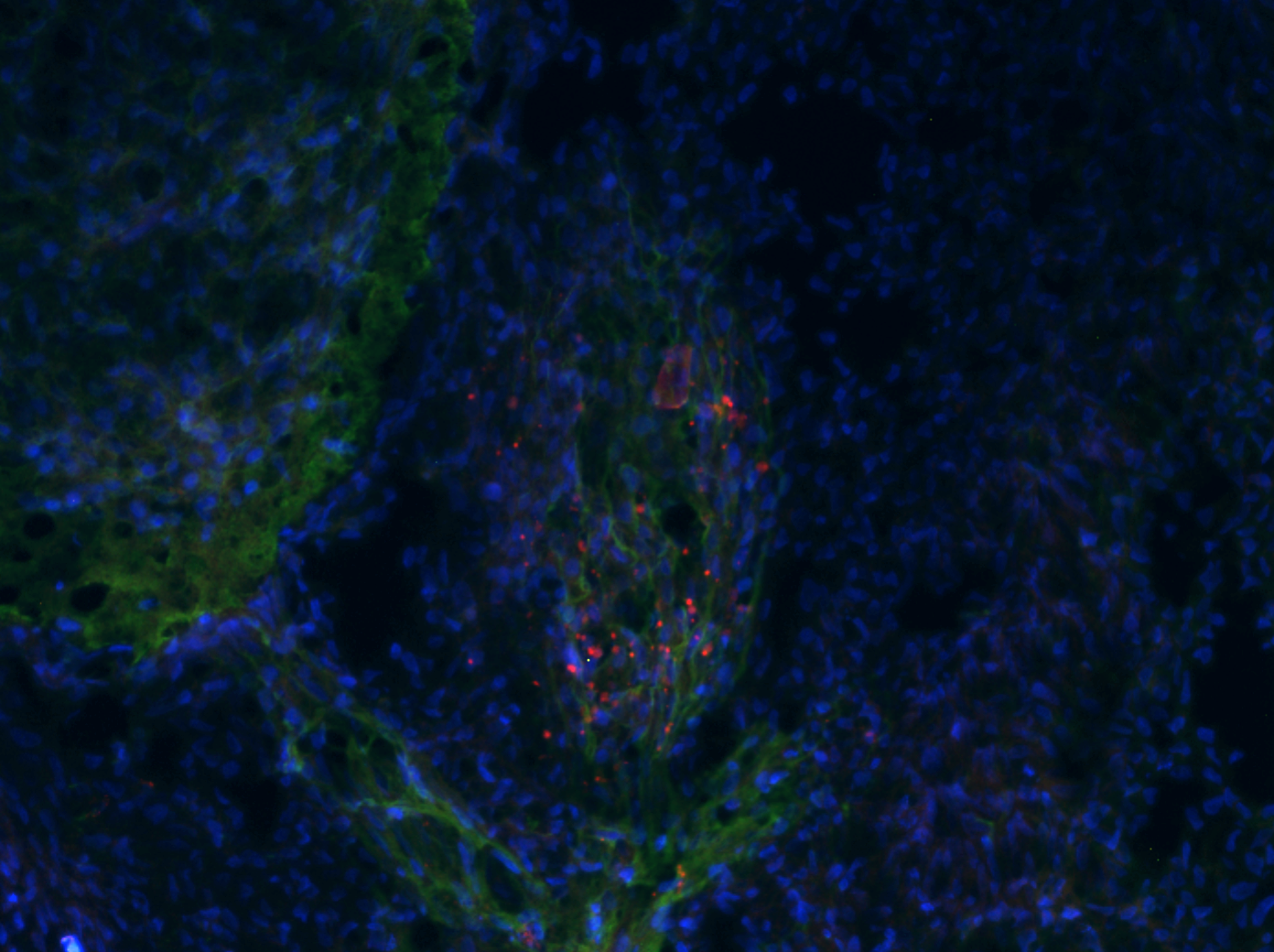

Supplement: S5 File — (ZIP) [file pone.0256484.s007.zip › S5 File/Figure S1O C Coste.tif]

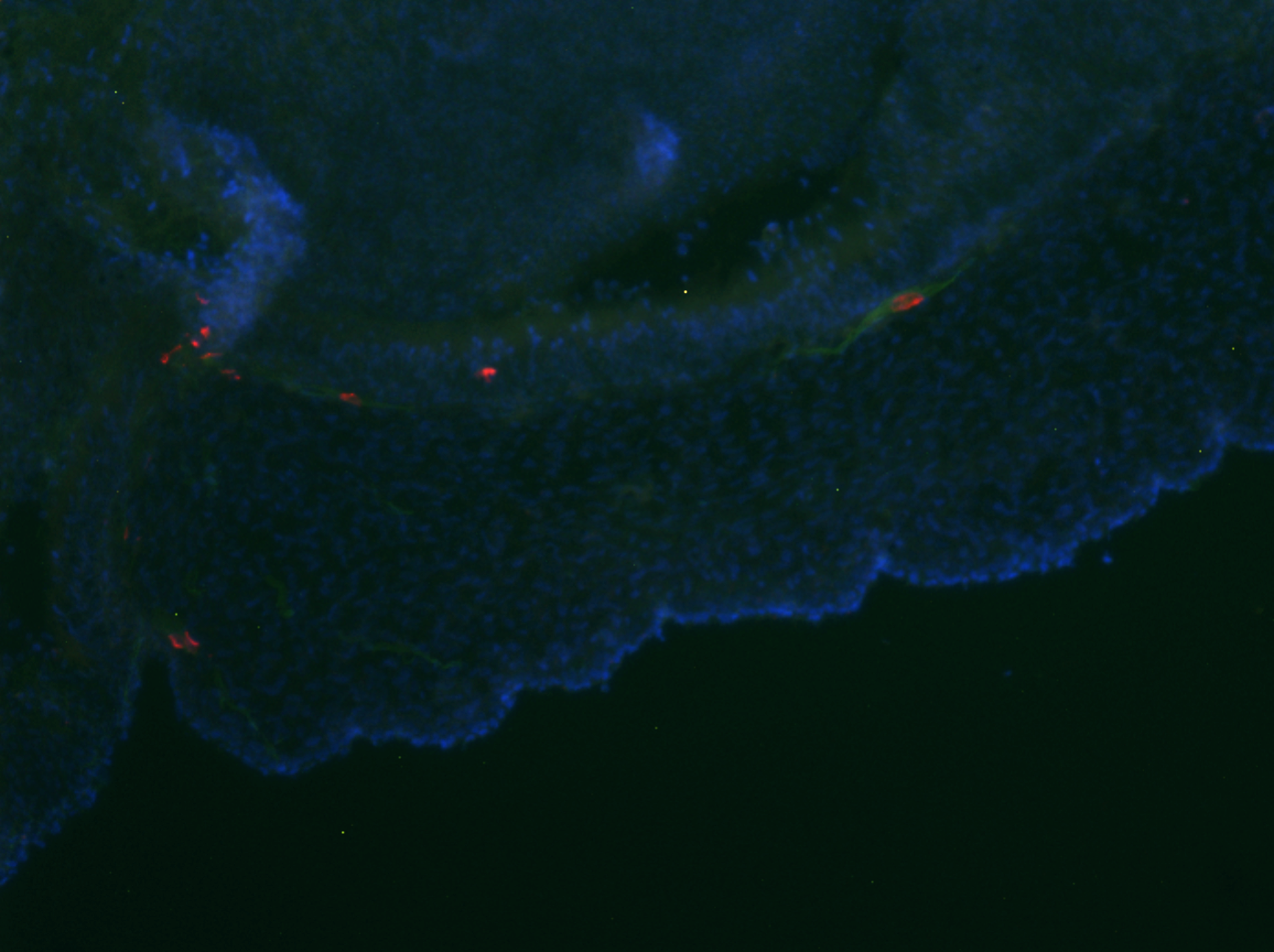

Supplement: S5 File — (ZIP) [file pone.0256484.s007.zip › S5 File/Figure S1P C Coste.tif]
